# Genome-wide association studies in the Japanese population identify seven novel loci for type 2 diabetes

**DOI:** 10.1038/ncomms10531

**Published:** 2016-01-28

**Authors:** Minako Imamura, Atsushi Takahashi, Toshimasa Yamauchi, Kazuo Hara, Kazuki Yasuda, Niels Grarup, Wei Zhao, Xu Wang, Alicia Huerta-Chagoya, Cheng Hu, Sanghoon Moon, Jirong Long, Soo Heon Kwak, Asif Rasheed, Richa Saxena, Ronald C. W. Ma, Yukinori Okada, Minoru Iwata, Jun Hosoe, Nobuhiro Shojima, Minaka Iwasaki, Hayato Fujita, Ken Suzuki, John Danesh, Torben Jørgensen, Marit E. Jørgensen, Daniel R. Witte, Ivan Brandslund, Cramer Christensen, Torben Hansen, Josep M. Mercader, Jason Flannick, Hortensia Moreno-Macías, Noël P. Burtt, Rong Zhang, Young Jin Kim, Wei Zheng, Jai Rup Singh, Claudia H. T. Tam, Hiroshi Hirose, Hiroshi Maegawa, Chikako Ito, Kohei Kaku, Hirotaka Watada, Yasushi Tanaka, Kazuyuki Tobe, Ryuzo Kawamori, Michiaki Kubo, Yoon Shin Cho, Juliana C. N. Chan, Dharambir Sanghera, Philippe Frossard, Kyong Soo Park, Xiao-Ou Shu, Bong-Jo Kim, Jose C. Florez, Teresa Tusié-Luna, Weiping Jia, E Shyong Tai, Oluf Pedersen, Danish Saleheen, Shiro Maeda, Takashi Kadowaki

**Affiliations:** 1 Laboratory for Endocrinology, Metabolism and Kidney Diseases, RIKEN Center for Integrative Medical Sciences, Yokohama 230-0045, Japan; 2 Laboratory for Statistical Analysis, RIKEN Center for Integrative Medical Sciences, Yokohama 230-0045, Japan; 3 Laboratory for Omics Informatics, Omics Research Center, National Cerebral And Cardiovascular Center, Suita 565-8565, Japan; 4 Department of Diabetes and Metabolic Diseases, Graduate School of Medicine, University of Tokyo, Tokyo 113-0033, Japan; 5 Department of Diabetes Endocrinology, Metabolism and Rheumatology, Tokyo Medical University, Tokyo 160-0023, Japan; 6 Department of Metabolic Disorder, Diabetes Research Center, Research Institute, National Center for Global Health and Medicine, Tokyo 162-8655, Japan; 7 The Novo Nordisk Foundation Center for Basic Metabolic Research, Faculty of Health and Medical Sciences, University of Copenhagen, Copenhagen DK-2200, Denmark; 8 Department of Biostatistics and Epidemiology, University of Pennsylvania, Philadelphia, Pennsylvania 19104-6021, USA; 9 Saw Swee Hock School of Public Health, National University of Singapore, Singapore 138672, Singapore; 10 Unidad de Biología Molecular y Medicina Genómica, Instituto de Investigaciones Biomédicas UNAM/Instituto Nacional de Ciencias Médicas y Nutrición Salvador Zubirán, Mexico City C.P.14000, Mexico; 11 Department of Endocrinology and Metabolism, Shanghai Key Laboratory of Diabetes Mellitus, Shanghai Diabetes Institute, Shanghai Jiao Tong University Affiliated Sixth People's Hospital, Shanghai 200233, China; 12 Division of Structural and Functional Genomics, Center for Genome Science, National Institute of Health, Chungcheongbuk-do 28159, Korea; 13 Division of Epidemiology, Department of Medicine, Vanderbilt Epidemiology Center, Vanderbilt-Ingram Cancer Center, Vanderbilt University School of Medicine, Nashville, Tennessee 37203-1738, USA; 14 Department of Internal Medicine, Seoul National University Hospital, Seoul 03080, Korea; 15 Center for Non-Communicable Diseases, Karachi, Pakistan; 16 Center for Human Genetic Research, Massachusetts General Hospital, Boston, Massachusetts 02114, USA; 17 Department of Medicine and Therapeutics, The Chinese University of Hong Kong, Hong Kong, China; 18 Hong Kong Institute of Diabetes and Obesity, The Chinese University of Hong Kong, Hong Kong, China; 19 Li Ka Shing Institute of Health Sciences, The Chinese University of Hong Kong, Hong Kong, China; 20 Department of Human Genetics and Disease Diversity, Graduate School of Medical and Dental Sciences, Tokyo Medical and Dental University, Tokyo 113-8510, Japan; 21 First Department of Internal Medicine, University of Toyama, Toyama 930-0194, Japan; 22 Health Administration Center, University of Toyama, Toyama 930-0194, Japan; 23 Department of Public Health and Primary Care, University of Cambridge CB1 8RN, UK; 24 Wellcome Trust Sanger Institute, Wellcome Trust Genome Campus Hinxton, Cambridge CB10 1RQ, UK; 25 NIHR Blood and Transplant Research Unit in Donor Health and Genomics, Department of Public Health and Primary Care, University of Cambridge, Cambridge CB1 8RN, UK; 26 Research Centre for Prevention and Health, Capital Region of Denmark, Glostrup DK-2600, Denmark; 27 Faculty of Health and Medical Sciences, Department of Public Health, University of Copenhagen, Copenhagen 2200, Denmark; 28 Faculty of Medicine, University of Aalborg, Aalborg 9220, Denmark; 29 Steno Diabetes Center, Gentofte 2820, Denmark; 30 Department of Public Health, Aarhus University, Aarhus 8000, Denmark; 31 Danish Diabetes Academy, Odense 5000, Denmark; 32 Department of Clinical Biochemistry, Lillebaelt Hospital, Vejle 7100, Denmark; 33 Institute of Regional Health Research, University of Southern Denmark, Odense 5230, Denmark; 34 Medical Department, Lillebaelt Hospital, Vejle 7100, Denmark; 35 Program in Medical and Population Genetics, Broad Institute of Harvard and MIT, Cambridge, Massachusetts 02142, USA; 36 Joint BSC-CRG-IRB Research Program in Computational Biology, Barcelona Supercomputing Center, Barcelona 08034, Spain; 37 Center for Human Genetic Research and Diabetes Research Center (Diabetes Unit), Massachusetts General Hospital, Boston, Massachusetts 02114, USA; 38 Department of Molecular Biology, Harvard Medical School, Boston, Massachusetts 02115, USA; 39 Universidad Autónomas Metropolitana, Mexico City 09340, Mexico; 40 Central University of Punjab, Bathinda, Punjab 151001, India; 41 Health Center, Keio University, Tokyo 160-8582, Japan; 42 Department of Medicine, Shiga University of Medical Science, Otsu 520-2192, Japan; 43 Grand Tower Medical Court Life Care Clinic, Hiroshima 730-0012, Japan; 44 Department of Internal Medicine, Kawasaki Medical School, Kurashiki 701-0192, Japan; 45 Department of Metabolism and Endocrinology, Juntendo University Graduate School of Medicine, Tokyo 113-8421, Japan; 46 Sportology Center, Juntendo University Graduate School of Medicine, Tokyo 113-0034, Japan; 47 Division of Metabolism and Endocrinology, Department of Internal Medicine, St Marianna University School of Medicine, Kawasaki 216-8511, Japan; 48 RIKEN Center for Integrative Medical Sciences, Yokohama 230-0045, Japan; 49 Department of Biomedical Science, Hallym University, Chunchon, Gangwon-do 24252, Republic of Korea; 50 Department of Pediatrics, University of Oklahoma Health Sciences Center, Oklahoma City, Oklahoma 73104, USA; 51 Department of Pharmaceutical Sciences, University of Oklahoma Health Sciences Center, Oklahoma City, Oklahoma 73104, n; 52 Department of Internal Medicine, Seoul National University College of Medicine, Seoul 03080, Korea; 53 Department of Molecular Medicine and Biopharmaceutical Sciences, Graduate School of Convergence Science and Technology, Seoul National University, Seoul 03080, Korea; 54 Department of Medicine, Harvard Medical School, Boston, Massachusetts 02115, USA; 55 Yong Loo Lin School of Medicine, National University of Singapore, Singapore 119228, Singapore; 56 Duke-National University of Singapore Graduate School, Singapore 169857, Singapore; 57 Department of Advanced Genomic and Laboratory Medicine, Graduate School of Medicine, University of the Ryukyus, Nishihara 903-0215, Japan; 58 Division of Clinical Laboratory and Blood Transfusion, University of the Ryukyus Hospital, Nishihara 903-0215, Japan

## Abstract

Genome-wide association studies (GWAS) have identified more than 80 susceptibility loci for type 2 diabetes (T2D), but most of its heritability still remains to be elucidated. In this study, we conducted a meta-analysis of GWAS for T2D in the Japanese population. Combined data from discovery and subsequent validation analyses (23,399 T2D cases and 31,722 controls) identify 7 new loci with genome-wide significance (*P*<5 × 10^−8^), rs1116357 near *CCDC85A*, rs147538848 in *FAM60A*, rs1575972 near *DMRTA1*, rs9309245 near *ASB3*, rs67156297 near *ATP8B2*, rs7107784 near *MIR4686* and rs67839313 near *INAFM2*. Of these, the association of 4 loci with T2D is replicated in multi-ethnic populations other than Japanese (up to 65,936 T2Ds and 158,030 controls, *P*<0.007). These results indicate that expansion of single ethnic GWAS is still useful to identify novel susceptibility loci to complex traits not only for ethnicity-specific loci but also for common loci across different ethnicities.

## Abstract (web summary)

Here, Imamura *et al*. conduct meta-analysis of genome-wide association studies to identify novel susceptibility loci for type 2 diabetes (T2D) in the Japanese population. By doing so, this study shows that both ethnicity-specific and ethnically-shared genetic loci can contribute to T2D risk.

## Graphical abstract

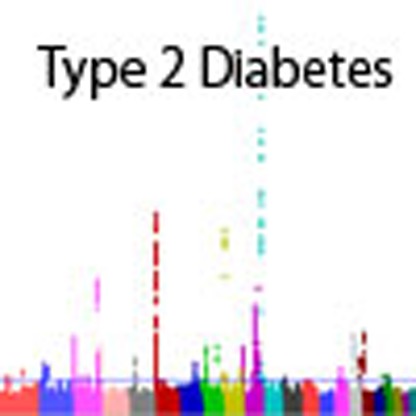

To date, more than 80 susceptibility loci for type 2 diabetes (T2D) have been identified through genome-wide association studies (GWAS)^1^,^2^,^3^,^4^,^5^,^6^,^7^,^8^,^9^,^10^,^11^,^12^,^13^,^14^,^15^,^16^,^17^,^18^. However, the joint effects of these variants account for <10% of the heritability for T2D^10^,^19^. GWAS for T2D have been extensively conducted in populations of European descent and, accordingly, the majority of established T2D susceptibility genetic loci were originally identified by European GWAS^1^,^2^,^8^,^9^,^10^,^11^,^16^. Cumulative evidence suggests that Asian populations may be more genetically susceptible to T2D than populations with European ancestry^20^. In addition, there are significant interethnic differences in the risk allele frequency or in effect sizes at several loci, which may affect the power to detect associations in these populations^2^. On the other hand, both overlap in T2D susceptibility loci among different ancestry groups and coincident risk alleles at lead single-nucleotide polymorphisms (SNPs) across diverse populations have been reported, suggesting that causal variants at many of these loci are shared across different ancestry groups^12^. Moreover, a recently published transethnic GWAS has successfully identified seven novel T2D susceptibility loci by combining the association data from European, South Asian, East Asian and Mexican/Latinos GWAS^12^. Therefore, it is valuable to perform GWAS for T2D using non-European and European populations, to facilitate identification of both ethnicity-specific and common-susceptibility loci among different ethnic groups.

Four T2D GWAS loci discovered in a Japanese population earlier have been shown to be significantly associated with T2D in the largest European GWAS meta-analysis^10^: *KCNQ1* (refs 3, 4), *UBE2E2* (ref. 5), *C2CD4A*–*C2CD4B*^5^ and *ANK1* (ref. 6), highlighting that there are common loci conferring susceptibility to T2D among the different ethnic groups studied. Three additional loci (*MIR129-LEP*, *GPSM1* and *SLC16A11-SLC16A13*) have been identified by a large-scale Japanese GWAS (*n*=∼25,000) based on the imputation of genotypes using the 1000 Genomes Project data as a reference^7^. One of the findings, the association in the *SLC16A11–SLC16A13* was also confirmed in the Mexican GWAS study^15^.

To identify novel loci for susceptibility to T2D, we have expanded the Japanese GWAS data set by incorporating new Japanese GWAS data (9,817 T2D cases and 6,763 controls) with GWAS data in previously reported case–control individuals (5,646 T2D cases and 19,420 controls)^7^ followed by a validation study using independent Japanese case–control individuals (7,936 T2D and 5,539 controls) and multi-ethnic replication studies (East Asians: 12,554 T2D and 17,383 controls; Europeans: 38,947 T2D and 121,903 controls; South Asians: 10,587 T2D and 14,378 controls; and Mexian/Latinos: 3,848 T2D and 4,366 controls). As a result, we identify seven novel loci for T2D and the result indicates that expansion of single ethnic GWAS is still useful to identify novel susceptibility loci to complex traits.

## Results

### GWAS meta-analysis and validation in the Japanese population

**Figure 1:**
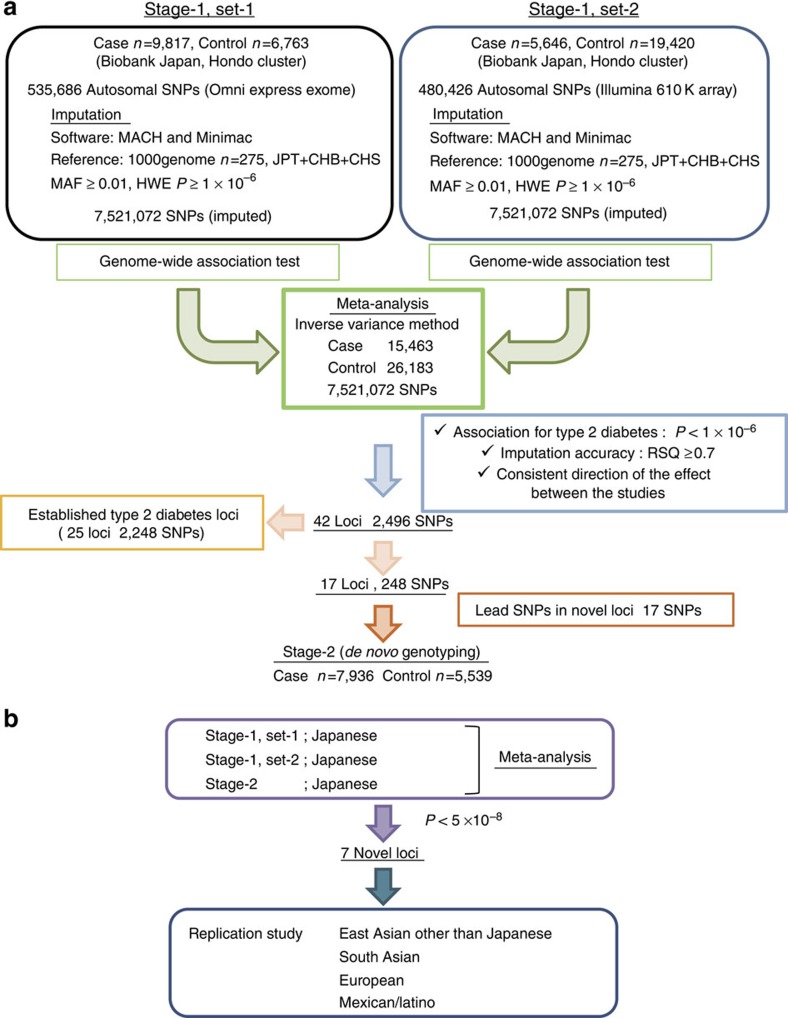
Outline of the present study. (**a**) GWAS meta-analysis and subsequent validation analysis. (**b**) Follow-up analyses for seven novel susceptibility loci for T2D in populations other than Japanese. SNP, single nucleotide polymorphism; MAF, minor allele frequency; HWE P, Hardy-Weinberg Equilibrium test P; RSQ, r square.

Imputed genotype dosage data for 9,817 T2D cases and 6,763 controls for 7,521,072 autosomal SNPs (Stage-1, set-1) were obtained and combined with an independent GWAS data of previously reported case–control individuals^7^ (Stage-1, set-2: 5,646 T2D cases and 19,420 controls; 7,521,072 autosomal SNPs), as shown in Fig. 1a. There was no obvious inflation in the quantile–quantile plots for each study (Stage-1: *λ*_GC_=1.13 and *λ*_GC_ adjusted for 1,000 cases and controls (*λ*_GC-1000_)^21^=1.012; Stage-2: *λ*_GC_=1.082 and *λ*_GC-1000_=1.009), as shown in Supplementary Fig. 1A,B. SNPs with a low imputation quality (*r*^2^<0.7 either in set-1 or set-2) or with an inconsistent direction of effect between the studies were excluded from the analysis. We obtained 42 loci exhibiting a suggestive association with T2D (*P*<1 × 10^−6^). The most significant association in this meta-analysis was rs2237896 located at intron 15 of *KCNQ1* (*P*=2.81 × 10^−70^), which was previously identified in Japanese GWAS^3^,^4^ (Supplementary Fig. 1C). Out of the 42 loci, 25 were previously established T2D susceptibility loci (Supplementary Table 1) and the remaining 17 were further evaluated using an independent Japanese case–control study (Stage-2: 7,936 T2D cases and 5,539 controls, multi-centre) and *de novo* genotyping (Supplementary Tables 2 and 3).

**Figure 2:**
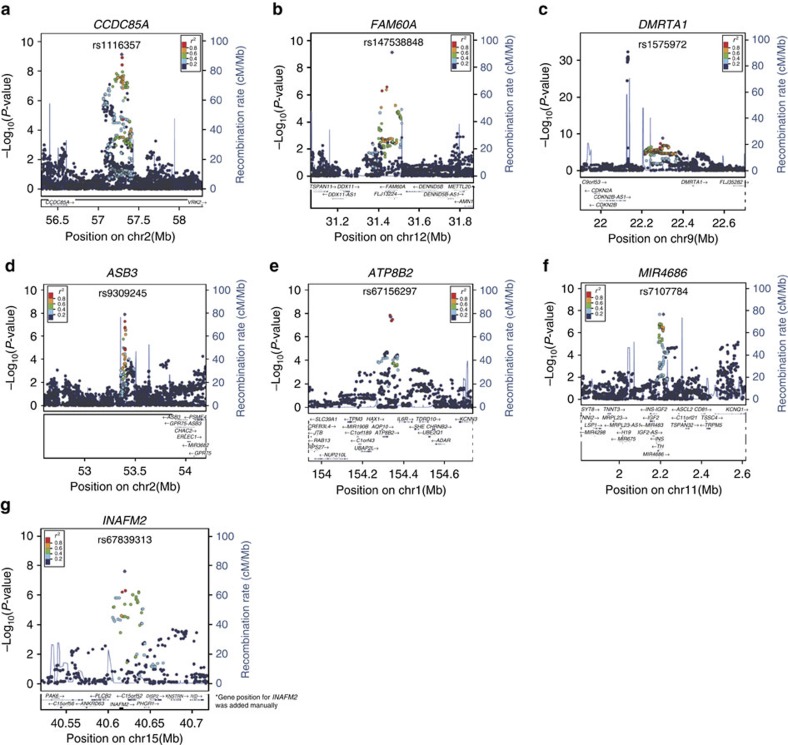
Regional association plots of the two-stage GWAS meta-analysis for seven novel T2D susceptibility loci in the Japanese. Each plot shows −log_10_ *P*-values against the chromosomal positions of SNPs in the specific region. The SNP with the strongest association signal (lead SNP) in each locus is represented as a purple diamond; the other SNPs are coloured according to the extent of LD with the lead SNP. Estimated recombination rates from the hg19/1000 Genomes Project March 2012 East Asian references are shown as light-blue lines. (**a**) *CCDC85A*, (**b**) *FAM60A*, (**c**) *DMRTA1*, (**d**) *ASB3*, (**e**) *ATP8B2*, (**f**) *MIR4686* and (**g**) *INAFM2.* *Gene position for *INAFM2* was added manually.

To explore the novel T2D susceptibility loci, we combined the association data for the candidate SNPs from all the Japanese case–control samples (Stage-1 set-1, Stage-1 set-2 and Stage-2) using a meta-analysis (Fig. 1b and Supplementary Table 3) and identified seven T2D susceptibility loci with a significant association (*P*<5 × 10^−8^; Table 1, Fig. 2 and Supplementary Table 3): rs1116357 near *CCDC85A* (*P*=6.97 × 10^−10^, odds ratio (OR)=1.09, 95% confidence interval (CI)=1.06–1.12), rs147538848 in *FAM60A* (*P*=7.83 × 10^−10^, OR=1.11, 95% CI=1.07–1.15), rs1575972 near *DMRTA1* (*P*=1.50 × 10^−9^, OR=1.19, 95% CI=1.13–1.26), rs9309245 near *ASB3* (*P*=1.25 × 10^−8^, OR=1.10, 95% CI=1.07–1.14), rs67156297 near *ATP8B2* (*P*=1.95 × 10^−8^, OR=1.14, 95% CI=1.09–1.19), rs7107784 near *MIR4686* (*P*=2.07 × 10^−8^, OR=1.14, 95% CI=1.09–1.20) and rs67839313 near *INAFM2* (*P*=2.42 × 10^−8^, OR=1.09, 95% CI=1.06–1.12). The effect sizes (OR) of these seven SNPs were similar before and after adjusting for age, sex and body mass index (BMI) (Supplementary Table 4). The rs1575972 locus in *DMRTA1* was located 170 kbp away from the T2D locus *CDKN2A/B* at 9p21 (refs 10, 22, 23, 24) and the rs7107784 locus near *MIR4686* was located ∼620 kbp upstream of *KCNQ1* (refs 3, 4). The linkage disequilibrium (LD) between rs1575972 and rs10811661, which was a lead SNP within the *CDKN2A/B* locus^10^, was weak (JPT: *r*^2^=0.01, CEU: *r*^2^=0.02). The association of rs1575972 was significant even after conditioning on rs10811661 (*P* for meta-analysis=2.45 × 10^−9^, OR=1.19, 95% CI=1.12–1.26; Supplementary Table 5); therefore, we considered the rs1575972 locus as a novel T2D susceptibility locus, independent of the *CDKN2A/B* locus. We also observed that the rs7107784 locus near *MIR4686* was not in LD with rs2237897 (JPT: *r*^2^<0.01, CEU: *r*^2^<0.01), which was a lead SNP within the *KCNQ1* locus^3^, and the association was similar after conditioning on rs2237897 (*P* for meta-analysis=2.75 × 10^−8^, OR=1.16, 95% CI=1.10–1.22; Supplementary Table 6).

We also examined the association of these seven SNPs with glycaemic traits in Stage-2 control individuals, including fasting plasma glucose, homeostasis model assessment (HOMA) of β-cell function (HOMA-β) and HOMA of insulin resistance (IR). However, we did not detect any significant associations between the T2D risk alleles and these glycaemic traits (*P*≥0.0024 Supplementary Table 7). We also searched the publicly available European GWAS data^11^,^25^,^26^ (MAGIC, http://www.magicinvestigators.org) and found that the T2D risk allele at the *DMRTA1* locus (rs11791293-C; proxy for rs1575972-T, CEU *r*^2^=1) and at the *MIR4686* locus (rs7111341-T; proxy for rs7107784-G, CEU *r*^2^=0.95) were associated with a decrease in fasting plasma insulin (FPI) (*P*=0.0039; Supplementary Table 8) and with an increase in FPI (*P*=0.0066; Supplementary Table 8), respectively, although these associations were not statistically significant (*P*≥0.0008=0.05/63 (7 SNPs × 9 traits)).

### Examination of seven novel loci in diverse ethnic groups

We analysed the association of these seven variants with disease susceptibility in populations other than Japanese. We obtained association data for four ethnic groups using *de novo* genotyping, *in silico* replication and by examining publicly available GWAS data^10^ including East Asian (*n*=up to 29,937: 12,554 T2Ds and 17,383 controls), South Asian (*n*=up to 24,965: 10,587 T2Ds and 14,378 controls), European (*n*=up to 160,850: 38,947 T2Ds and 121,903 controls) and Mexican (*n*=up to 8,214: 3,848 T2Ds and 4,366 controls) populations (Supplementary Table 9). Meta-analyses of the combined data from the four non-Japanese ethnicities indicated that four SNP loci, namely rs147538848 in *FAM60A*, rs1575972 near *DMRTA1*, rs7107784 near *MIR4686* and rs67839313 near *INAFM2* were associated with the disease after Bonferroni's correction (*P*<0.00714=0.05/7; Table 2). The disease association of these four SNPs was further corroborated by combining the Japanese data with the multi-ethnic replication data sets (Supplementary Table 10). The rs67156297 locus in *ATP8B2* was nominally associated with T2D in the combined meta-analysis for multi-ethnic groups other than the Japanese populations. We did not detect any disease association for the remaining two SNP loci other than in the Japanese population; however, the effect direction for each of the seven loci was consistent with that in the Japanese population.

### Sex- and BMI-stratified analyses in the Japanese population

We performed BMI-stratified (BMI<25 or≥25) and sex-stratified analyses in the novel and established GWAS loci, to determine whether significant heterogeneity in allelic effects existed between non-obese and obese individuals or males and females in the Japanese population. BMI-stratified analysis for 83 previously established loci revealed evidence of significant heterogeneity in the effect size between non-obese and obese individuals at *KCNQ1* (*P* for heterogeneity=8.89 × 10^−5^; Supplementary Table 11). The effect size of *KCNQ1* was greater in the non-obese group than in the obese group (Supplementary Table 11). In sex-stratified analyses, individual established loci did not show significant heterogeneity in effect sizes between men and women (*P*>6 × 10^−4^; Supplementary Table 12).

For the seven novel T2D-associated loci identified in this study, no significant heterogeneity was detected in BMI-stratified or sex-stratified analyses (Supplementary Tables 13 and 14).

### Fine mapping analyses for established T2D loci

We examined the association data of 83 previously identified T2D susceptibility loci in the Japanese GWAS meta-analysis data (Supplementary Data 1 and Supplementary Fig. 2). Variants at 19 loci were found associated with T2D at a genome-wide level of significance and additional 30 loci were determined to be significantly associated with T2D (*P*<6.02 × 10^−4^=0.05/83). Of the above 49 significant associations, *ADCY5*, *HNF1A* and *PRC1* were not previously evaluated in the Japanese population, because the lead SNPs within these loci in the European GWAS (rs11708067 and rs11717195 at the *ADCY5* locus^9^,^10^, rs12427353 and rs7957197 at the *HNF1A* locus^8^,^10^ and rs8042680 at the *PRC1* locus^8^) were monoallelic in the Japanese population. In this study, rs79223353 at the *ADCY5* locus, rs55783344 at the *HNF1A* locus and rs79548680 at the *PRC1* locus were determined to be significantly associated with T2D (*P*<6.02 × 10^−4^; Supplementary Data 1 and Supplementary Fig. 3). Meta-analysis combining the GWAS data with *de novo* genotyping data for Stage-2 individuals revealed that the association of rs79223353 within the *ADCY5* locus and rs79548680 within the *PRC1* locus reached genome-wide significance in the Japanese population (Supplementary Table 15). We did not detect any disease-associated SNPs within the 16 loci (9 derived from European GWAS, 3 from East Asian, 2 from trans-ethnic, 1 from South Asian and 1 from African American, *P*≥0.05) using fine mapping analyses (Supplementary Data 1 and Supplementary Fig. 2). We also identified a secondary association signal located at *EXOC6* near the *IDE-HHEX* locus^10^. The associations of rs78627331 and rs34773007 within the *EXOC6* locus were significant after conditioning on rs1111875 (*r*^2^=0.01 for rs78627331 and *r*^2^=0.04 for rs34773007 in *JPT*), which was a previously reported lead SNP within the *IDE-HHEX* locus (*P*=1.49 × 10^−8^ for rs78627331, *P*=2.20 × 10^−8^ for rs34773007; Supplementary Table 16).

### Drug targets search by a bioinformatics approach

**Figure 3:**
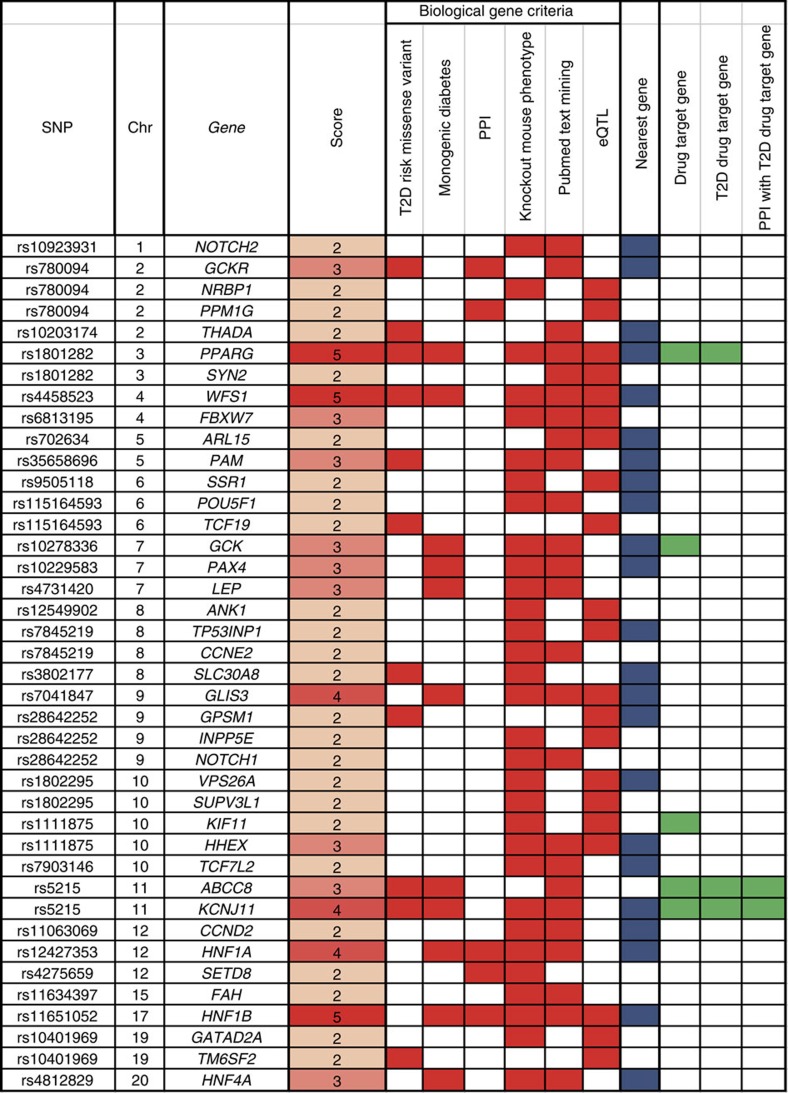
Prioritized biological T2D risk genes. The gene score for each gene was calculated by summing up the number of criteria satisfied (filled red box indicates criterion satisfied; 40 genes with a score ≥2 out of 286 genes included in the T2D risk loci were defined as ‘biological candidate genes'; see Supplementary Data 4). Filled blue boxes indicate the nearest gene to the T2D risk SNP. Filled green boxes indicate overlap with drug target genes.

We applied the genetic information from previously reported and the present GWAS, to investigate potential drug targets for the treatment of T2D. First, we defined 286 T2D potential risk genes located in any of the 90 T2D risk loci (7 novel T2D loci that were identified in the present study and 83 previously identified T2D loci; see Supplementary Note). Among the 286 genes, by using a previously described scoring system^27^, we selected 40 genes with a score of 2 or higher (Supplementary Note, Supplementary Tables 17–20, Supplementary Data 2 and 3, and Supplementary Figs 4 and 5) as ‘biological T2D risk genes' (Fig. 3 and Supplementary Data 4). In brief, we scored each of the 286 biological candidate genes by adopting the following six selection criteria and calculating the number of satisfied criteria as follows: (1) genes for which T2D risk SNPs or any of the SNPs in LD (*r*^2^≥0.80) with them were annotated as missense variants; (2) genes for which *cis*-eQTL genes of any of lymphoblastoid cell lines, adipose tissue or liver tissues were observed for T2D risk SNPs (*P*<0.05 for lymphoblastoid cell lines and adipose tissues, and *P*<0.004 for liver tissues); (3) monogenic diabetes genes; (4) genes for which at least three out of six associated phenotype labels (homeostasis/metabolism, liver/biliary system, endocrine/exocrine gland, growth/size/body, mortality/ageing and embryogenesis; *P*<9.2 × 10^−5^) were observed in knockout mouse^28^; (5) genes prioritized by PubMed text mining genes using GRAIL^29^ with gene-based *P*<0.05; and (6) genes prioritized by protein–protein interaction (PPI) network using DAPPLE^30^ with gene based *P*<0.05.

As these criteria exhibited weak correlations with each other (*r*^2^<0.34; Supplementary Fig. 5), each gene was given a score based on the number of criteria that were met (scores ranged from 0 to 6). Genes with a score of 2 or higher were defined as biological T2D risk genes.

We searched for overlapping genes between the 871 drug target genes corresponding to approved, in clinical trials or experimental drugs for various human diseases described in the previous report^27^, and the 40 biological T2D risk genes plus 712 genes that are known to have products that have direct PPI^30^ with the biological T2D risk gene products. We identified a total of 83 overlapping genes (Supplementary Fig. 6 and Supplementary Table 21). Fourteen drug target genes with approved T2D treatments demonstrated significant overlap with the 40 biological T2D risk genes and 712 genes with direct PPI (4 genes overlapped with 5.6-fold enrichment as determined using permutation analysis, *P*=0.0042; Supplementary Table 22 and Supplementary Fig. 6). The 871 drug target genes had overlap with the identified 83 genes, which is 1.8-fold more enrichment than would be expected by chance, but this is 3.1-fold less enrichment compared with overlap of the targets of T2D drugs (Supplementary Fig. 6).

**Figure 4:**
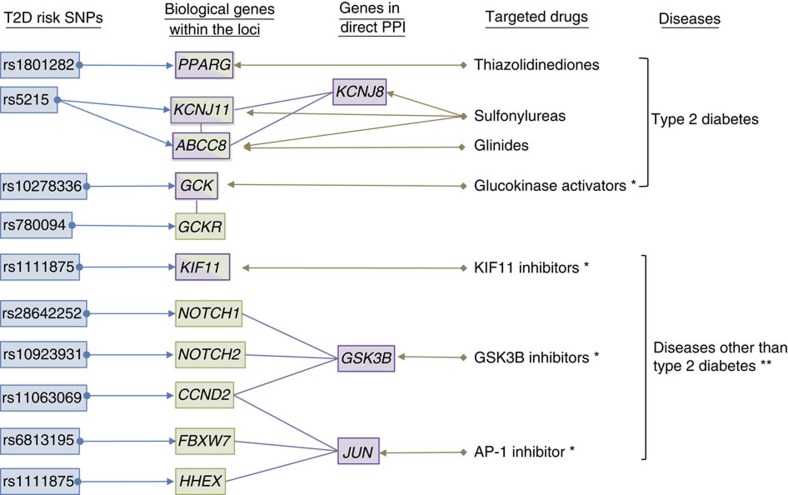
Connection of biological T2D risk genes to drug targets. Representative connections between T2D risk SNPs (blue), T2D biological genes (green), drug target genes (purple) and targeted drugs are shown (see Supplementary Table 21). *Compounds under clinical trial; **see Supplementary Table 23.

Of the 83 overlapping genes, 5 were biological T2D risk genes (*PPARG*, *KCNJ11*, *ABCC8*, *GCK* and *KIF11*; Fig. 4). Three of these are targets of approved T2D drug treatments: *PPARG*, thiazolidinediones; *KCNJ11*, sulfonylurea; *ABCC8*, sulfonylureas and glinide. *GCK* is a target gene of a GCK activator that was in clinical trials as of August 2014 (Supplementary Table 23). Of the remaining 78 genes, 2 genes exhibit PPI with 3 biological T2D risk gene products. *GSK3B* interacts with *NOTCH1*, *NOTCH2* and *CCND2*, whereas *JUN* interacts with *FBXW7*, *HHEX* and *CCND2*. Eight genes interact with 2 biological T2D risk gene products and 68 genes interact with a single biological T2D risk gene product (Supplementary Table 21). A list of therapeutic drugs that are currently under clinical trials targeting *GCK*, *KIF11*, *GSK3B* and *JUN* is shown in Supplementary Table 23.

## Discussion

In this study, we performed a GWAS meta-analysis in the Japanese population followed by validation using an independent Japanese sample. Integration of the results for ∼55,000 Japanese individuals identified 7 novel loci associated with T2D that reached genome-wide significance. In a subsequent transethnic meta-analysis, four loci were confirmed and one locus was suggested as common susceptibility loci for T2D in populations other than the Japanese population.

GWAS have been extensively performed in diverse ethnic groups, including populations of European, East Asian, South Asian and Mexican decent^1^,^2^,^3^,^4^,^5^,^6^,^7^,^8^,^9^,^10^,^11^,^12^,^13^,^14^,^15^,^16^,^17^,^18^. To this point, the sample size of GWAS for European populations has grown to over 100,000 (ref. 10) and these studies have identified nearly 50 loci associated with T2D. GWAS on populations of non-European origin and transethnic GWAS meta-analysis have identified more than 30 loci associated with T2D, which were not detected in earlier European GWAS^3^,^4^,^5^,^6^,^7^,^12^,^13^,^14^,^15^,^18^. Among these, several loci have been shown to be associated with T2D in larger European populations^10^, which suggests that further expansion of GWAS for non-European populations could prove useful in identifying additional susceptibility loci associated with T2D.

Among the seven novel loci identified in this study, rs147538848 in *FAM60A*, rs1575972 near *DMRTA1*, rs7107784 near *MIR4686* and rs67839313 near *INAFM2* were shown to be common susceptibility loci for T2D across different ethnicities, although the significance of the association differed among individual ethnic groups for several loci.

rs147538848 is located in the intron of *FAM60A*, which encodes a subunit of the Sin3 deacetylase complex (Sin3/HDAC1) that has been shown to be important for the repression of genes encoding components of the transforming growth factor-β signalling pathway^31^. Studies using a rat intrauterine growth retardation model have suggested that the Sin3/HDAC1 complex may negatively regulate the expression level of pancreatic and duodenal homeobox 1 (*PDX1*), which is known as an important transcription factor for the development of pancreas and β-cell maturation^32^ via histone modification of its proximal promoter^33^. A T2D risk allele at the *FAM60A* locus might contribute to disease susceptibility by impairing the transcriptional regulation of genes that are important for glucose metabolism.

*INAFM2* encodes InaF-motif containing 2 and has previously been known as Osteogenesis upregulated transcript 1 (*OGU1*) or long intergenic non-protein coding RNA 984 (*LINC00984*), which is a putative long non-coding RNA. Although the expression of *OGU1* has been shown to be upregulated during osteogenesis^34^, the function of *INAFM2* encoding protein is still unknown. Around rs67839313, there are two plausible genes for susceptibility to T2D: *PLCB2* and *DISP2*. *PLCB2* encodes phospholipase C isoform β-2 and phospholipase C is a known regulator of insulin secretion through hydrolysis of islet phosphoinositide pools^35^. Therefore, it is feasible that this locus is associated with impaired glucose-stimulated insulin secretion machinery. *DISP2* encodes dispatched homologue 2, which is a cell surface marker on insulin-positive cells^36^. Although the functional role of this molecule in glucose homeostasis is not well understood, it is potentially involved in the maturation of pancreatic β-cells or it might have a role in already matured pancreatic β-cells.

The effect size for the T2D association of rs1575972 near *DMRTA1* was similar among all populations in this study, except for the Mexican/Latino population (Table 2 and Supplementary Table 10). The risk allele of rs1575972 in the *DMRTA1* locus was nominally correlated with a decrease in FPI (Supplementary Table 8), which suggests that this locus might contribute to T2D susceptibility through affecting insulin secretion in pancreatic β-cells. The *DMRTA1* encodes doublesex and mab-3-related transcription factor-like family A1, which has been recently reported to be involved in neuronal development by regulating the Pax6-Neurog2 transcriptional cascade^37^. Although the relevance of *DMRTA1* to pancreatic development has not been established, *DMRTA1* might play a role in β-cell development, because Pax6 and Neurog3, other member of the neurogenin subfamily, are key transcriptional regulators of pancreatic endocrine cell differentiation^38^.

*INS*, *IGF-2* and *TH* are located at approximately rs7107784 near the *MIR4686* locus. *IGF-2* plays a key role in embryonic growth and may also influence body weight in adulthood^39^, and *TH* (tyrosine hydroxylase) has been shown to play a role in β-cell development^40^. This locus is known to be associated with risk of type-1 diabetes (rs1004446-C, 45 kbp from rs7107784; *r*^2^=0.003, *D*′=0.275)^41^. The risk allele of rs7107784-G is nominally associated with the increase of FPI levels in the European population (MAGIC data; Supplementary Table 8) and an increase of HOMA-IR in our Japanese data set (Supplementary Table 7). This suggests that the effects of rs7107784-G are probably not mediated by an impairment of insulin production or secretion, but rather by an impairment of insulin sensitivity.

rs67156297 near *ATP8B2* was nominally associated with T2D in the transethnic replication meta-analysis (Table 2 and Supplementary Table 10). *ATP8B2* encodes a member of the P4 family of ATPases (type 4P-type ATPase), which are multispan transmembrane proteins that have been implicated in phospholipid translocation from the exoplasmic to the cytoplasmic membrane leaflet^42^. The role of *ATP8B2* in the pathogenesis of T2D has not been established. However, another member of the P4 ATPase family, *atp10a*, has been shown to be important for the biogenesis and/or membrane-directed trafficking of Glut4 receptors, and loss-of-function of *atp10a* induces IR and obesity in mice^42^.

The remaining two loci, rs1116357 near *CCDC85A* and rs9309245 near *ASB3*, were not associated with T2D (*P*>0.05) in the replication meta-analysis for non-Japanese populations, which suggests that the effect of these loci might be specific to the Japanese population. As heterogeneity in effect sizes was observed for rs1116357 or rs9309245 between Japanese and other ethnic groups, including European, South Asian and Mexican (Supplementary Tables 25 and 27), two possibilities might exist for the two SNP loci: (1) the LD between the causal alleles and the Japanese lead SNPs are consistent across the populations, but the risk alleles have effects only in the Japanese, and (2) the causal alleles are in LD with these SNPs only in the Japanese. By a systematic evaluation for effect sizes and LDs within these loci, we did not identify any SNPs associated with T2D in European populations, which are in LD with our lead SNPs in the Japanese, whereas not in LD in European populations (Supplementary Fig. 8 and Supplementary Tables 26 and 27). Therefore, the causal allele in the two loci might have an effect only in Japanese populations; however, further evaluation is required to elucidate the precise mechanism how these loci contribute to T2D susceptibility in the Japanese.

While searching for potential drug targets for T2D using a systematic bioinformatics approach, 83 overlapping genes were identified from 752 genes (40 biological T2D GWAS genes and 712 genes that encode products in direct PPI with 40 biological T2D GWAS genes) and 871 drug target genes for various human diseases^27^. Of these, 5 were T2D GWAS genes: *PPARG*, *KCNJ11*, *ABCC8*, *GCK* and *KIF11*. *PPARG*, *KCNJ11* and *ABCC8* have approved T2D treatment options. In addition, a GCK activator is currently undergoing clinical trials for the treatment of T2D. *KIF11*, which encodes kinesin family member 11 (also known as EG5), has been shown to be involved in regulating cell mitosis and inhibitors targeting this gene product have been developed as chemotherapeutic agents in the treatment of cancer^43^. Although the role of *KIF11* in the regulation of glucose metabolism has not been well established, a recent study reported that knockdown of *KIF11* using small interfering RNA resulted in increased glycogenesis in human primary hepatocytes^44^. Thus, a *KIF11* inhibitor might ameliorate glucose homeostasis by suppressing gluconeogenesis from the liver.

We identified two genes, *GSK3B* and *JUN*, which directly interact with multiple biological T2D susceptibility genes. *GSK3B* encodes glycogen synthase kinase 3β, which is a constitutively active multifunctional serine/threonine kinase and is involved in diverse physiological pathways, including metabolism, cell cycle regulation, gene expression, development, oncogenesis and neuroprotection^45^. Several studies using *Gsk3b*-modified mouse models have suggested that inhibition of *GSK3B* function may have beneficial effects on glucose metabolism through pancreatic β-cell preservation or enhancement of insulin-stimulated glycogen synthase regulation and glycogen deposition^45^,^46^,^47^. Currently, *GSK3B* inhibitors are under clinical trial for the treatment of cancers (Supplementary Table 23), but these compounds could also be potential treatments for T2D.

*JUN* encodes the proto-oncogene c-Jun and the role of c-Jun in the pathogenesis of T2D is not well understood. However, c-Jun has been shown to decrease the expression of the human insulin gene by repressing insulin promoter activity^48^. c-Jun is a transactivation component of the heterodimeric transcription factor AP-1 and activated through phosphorylation of serines 63 and 73 by Jun N-terminal kinase 2 (ref. 49), and inhibition of JNK has been shown to ameliorate glucose intolerance in a mouse model for T2D^50^. Currently, *AP-1* inhibitor is under clinical trial for the treatment of rheumatoid arthritis (Supplementary Table 23) and might also be potential treatments for T2D.

Although these results suggest that these loci are potential therapeutic targets for treating T2D, the pipeline used to identify these genes has some limitations. As eQTL effects have often been observed for genes far from each locus, it is possible that some biological genes located outside of LD block in each locus were overlooked. In addition, the selection criteria for PubMed text-mining or knockout mouse studies were based on the known functions; therefore, T2D-associated genes whose functions have not been established may have been missed. The number of criteria that were met for individual genes were simply summed for scoring, although the relative impact of the six criteria used here on biological significance may not be equal. We used the previously described scoring method^27^, to prioritize genes in an objective manner; however, it would be worthwhile to refine the pipeline by modifying the selection criteria for genes in future studies. Finally, the potential therapeutic targets or treatments identified through the *in silico* pipeline have not yet been validated through an experimental approach. Furthermore, *in vivo* evaluation is essential to clarify the therapeutic effect of these potential T2D treatments.

In conclusion, we have identified seven novel T2D susceptibility loci using a large-scale Japanese GWAS meta-analysis. The T2D association for four of these was also observed in non-Japanese populations. In addition, we have proposed several new potential pharmacological targets for T2D treatment using a systematic bioinformatics approach. These results indicate that expansion of single ethnic GWAS is still useful to identify novel susceptibility loci to complex traits not only for ethnicity-specific but also for common loci across different ethnicities. Moreover, systematic approaches for integrating the findings of genetic, biological and pharmacological studies could be useful for developing new T2D treatments, although additional pipeline refinement would be required.

## Methods

### Subjects

*Discovery stage (Stage-1)*. We selected T2D cases from individuals registered in BioBank Japan as having T2D (set-1 cases, *n*=9,817). Control groups consisted of individuals registered in BioBank Japan as not having T2D but with diseases other than T2D (cerebral aneurysm, oesophageal cancer, endometrial cancer, chronic pulmonary emphysema or glaucoma) or volunteers from the Osaka-Midosuji Rotary Club and Pharma SNP consortium (set-1 controls, *n*=6,763; Supplementary Table 24). We also used case and control individuals registered in the BioBank Japan that were previously analysed and reported (set-2 cases, *n*=5,646 and set-2 controls, *n*=19,420)^7^. There was no overlap in individuals in set-1 and set-2.

*Validation analysis (Stage-2)*. We examined 7,936 T2D cases from the BioBank Japan that were not included in the discovery stage and from subjects with T2D, who visited outpatient clinics at The University of Tokyo, Juntendo University, National Center for Global Health and Medicine, Hiranuma Clinic, St Marianna University School of Medicine, The Hiroshima Atomic Bomb Casualty Council Health Management Center, Kawasaki Medical School, Toyama University Hospital or Shiga University of Medical Science. We also examined 5,539 controls from individuals that enrolled during an annual health check-up at six institutions: The Hiroshima Atomic Bomb Casualty Council Health Management Center, The National Center for Global Health and Medicine, Keio University, Hiranuma Clinic, St Marianna University School of Medicine and Toyama University Hospital. T2D was diagnosed according to World Health Organization criteria^51^. We excluded individuals who were positive for antibodies against glutamic acid decarboxylase and those with diabetes due to liver dysfunction, steroids and other drugs that might raise glucose levels, malignancy or a monogenic disorder known to cause diabetes.

Clinical characteristics of Stage-1and Stage-2 participants are shown in Supplementary Table 24. Genomic DNA was extracted from peripheral leukocytes using the standard procedure. All individuals provided written informed consent to participate in this study. The protocol of this study conformed to the provisions of the Declaration of Helsinki and was approved by the ethical committees at the RIKEN Yokohama Institute and all other institutions.

### Genotyping and quality control in the discovery stage

Set-1 samples were genotyped using the Human Omni Express Exome Bead Chip. There were 535,686 autosomal SNPs that passed quality control, with a call rate ≥0.99, for Hardy–Weinberg equilibrium test *P* ≥1 × 10^−6^ in controls and minor allele frequency (MAF) ≥0.01. Set-2 samples were genotyped using the Illumina Human 610K SNP array. There were 480,426 autosomal SNPs that passed quality control and were used for further analysis. For sample quality control, we evaluated cryptic relatedness for each sample using an identity-by-state method and removed samples that exhibited second-degree or closer relatedness. We further performed principal component analysis to select individuals within the major Japanese (Hondo) cluster as reported previously^5^,^6^,^7^,^52^, and data for 16,580 individuals (9,817 T2D cases and 6,763 controls) in set-1 and 25,066 individuals (5,646 T2D cases and 19,420 controls) in set-2 were used in subsequent analyses. To evaluate the potential effect of population stratification, we used a quantile–quantile plot of the observed *P*-values (Supplementary Fig. 1A,B).

### Imputation

We performed genotype imputation using MACH and Minimac^53^,^54^ with individuals from the 1000 Genomes Project (phased JPT, CHB and Han Chinese South data *n*=275, March 2012) as reference populations^55^. We selected SNPs with MAF ≥0.01 and a Minimac software quality score (*r*^2^)≥0.7. Individual genotype dosage data were used for association studies using mach2dat^53^,^54^.

### Genotyping and quality control in the Stage-2 analysis

We genotyped 7,936 individuals with T2D and 5,539 controls using a multiplex PCR-Invader assay, as described previously^3^,^5^,^6^,^7^. Genotyping success rates <95% or concordance rates <99.9% were excluded from further evaluation.

### Follow-up analyses

We obtained follow-up analysis data (*n*=up to 223,966: 65,936 T2Ds and 158,030 controls) from multiple cohorts or a publicly available database, as described below.

*East Asian populations*. We obtained genotype data for up to 29,937 individuals (12,554 T2Ds and 17,383 controls), *de novo* genotyping from 2 cohorts and *in silico* replication data from 9 cohorts (Supplementary Table 9).

*South Asian populations*. We obtained *in silico* genotype data for a total of up to 24,965 individuals (10,587 T2Ds and 14,378 controls) from 6 cohorts (Supplementary Table 9).

*European populations*. We obtained genotype data for up to 160,850 individuals (38,947 T2Ds and 121,903 controls), *de novo* genotyping data from the Danish case–control study and from a publicly available database (DIAGRAM3 http://diagram-consortium.org/downloads.html)^10^. The Danish case–control study consisted of individuals from the Inter99 cohort^56^, Health2006 cohort^57^, Vejle Biobank^58^, T2D cases from the Danish ADDITION screening cohort^59^ and a T2D case–control study obtained at Steno Diabetes Center (SDC). Two SNPs were genotyped by Illumina MetaboChip in 8,781 individuals from Inter99, Health2006 and SDC, whereas four SNPs were genotyped by LGC Genomics, UK, in individuals from Inter99, Vejle Biobank, ADDITION and SDC samples (Supplementary Table 9).

*Mexican/Latino population*. We obtained *in silico* genotype data for up to 8,214 individuals (3,848 T2Ds and 4,366 controls) from the SIGMA Type 2 Diabetes Consortium (Supplementary Table 9).

Ethnicity was self-reported by the enroled individuals. For each study, approval was obtained from the institutional review boards of the participating institutions and written informed consent was obtained from all participants. We excluded association data obtained by imputed genotyped data with a low quality of imputation (*r*^2^<0.7 or info <0.7). Details of the study samples are described in Supplementary Table 9.

### Statistical analysis

The association between each SNP and T2D was assessed using the logistic regression test with an additive model with or without adjusting for age, sex and log-transformed BMI. We combined data from the each GWAS and our validation analyses using an inverse variance method and examined heterogeneity with a Cochran's *Q* test using METAL^60^. Regional association plots were generated using LocusZoom^61^.

We also performed quantitative traits analysis for fasting plasma glucose, HOMA-β and HOMA-IR using multiple linear regression analysis in an additive association model with or without adjusting for age, sex and log-transformed BMI. The Japanese samples studied here show skewed distribution values for BMI, HOMA-IR and HOMA-β; therefore, we have analysed the quantitative traits using log-transformed BMI, HOMA-IR and HOMA-β.

### Drug discovery

We performed a search for potential drug targets using genetic information of confirmed T2D susceptibility loci and publicly available bioinformatics tools^29^,^30^ and databases^28^,^62^,^63^,^64^,^65^ using a method that has been previously described by Okada *et al*.^27^ (Supplementary Note).

## Additional information

**Data availability:** Summary statistics of 2 Japanese GWAS (study 1: 9,817 cases, 6,763 controls; study2: 5,646 cases, 19,420 controls) for directly genotyped data are available through a NBDC Human Database website (http://humandbs.biosciencedbc.jp/en/.

**How to cite this article:** Imamura, M. *et al*. Genome-wide association studies in the Japanese population identifies seven novel loci for type 2 diabetes. *Nat. Commun.* 7:10531 doi: 10.1038/ncomms10531 (2016).

## Supplementary Material

Supplementary Information Supplementary Figures 1-8, Supplementary Tables 1-27, Supplementary Note and Supplementary References

Supplementary Data 1 Summary of GWAS meta-analysis (Stage-1: set-1 + set-2) for 83 established T2D susceptibility loci.

Supplementary Data 2 Mouse knockout phenotype

Supplementary Data 3 cis-eQTL analyses

Supplementary Data 4 Biological scores of the genes in the T2D risk loci

## Figures and Tables

**Table 1:** Newly identified T2D susceptibility loci achieving genome-wide significance (combined meta-analysis *P* <5 × 10^−8^) in Japanese populations.

**SNP ID Chr. Position (Build 37)**	**Alleles**		**Study**	**RAF**		**OR (95% CI)**	***P*****-value**	***P*****het**
**nearby gene**	**Risk**	**Other**		**Case**	**Control**			
rs1116357	**G**	A	Stage-1, set-1	0.292	0.269	1.12 (1.07–1.17)	6.55 × 10^−6^	
2			Stage-1, set-2	0.299	0.279	1.10 (1.05–1.15)	2.61 × 10^−5^	
57287411			Stage-2	0.294	0.284	1.05 (0.99–1.11)	8.65 × 10^−2^	
***CCDC85A***			**Combined**			**1.09 (1.06–1.12)**	**6.97 × 10**^**−10**^	**0.21**
rs147538848	**A**	G	Stage-1, set-1	0.205	0.190	1.11 (1.05–1.18)	5.81 × 10^−4^	
12			Stage-1, set-2	0.199	0.184	1.12 (1.06–1.18)	1.15 × 10^−4^	
31466613			Stage-2	0.210	0.193	1.11 (1.04–1.18)	1.10 × 10^−3^	
***FAM60A***			**Combined**			**1.11 (1.07–1.15)**	**7.83 × 10**^**−10**^	**0.99**
rs1575972	**T**	A	Stage-1, set-1	0.947	0.935	1.24 (1.13–1.36)	8.64 × 10^−6^	
9			Stage-1, set-2	0.943	0.935	1.17 (1.07–1.29)	1.13 × 10^−3^	
22301092			Stage-2	0.950	0.942	1.16 (1.04–1.29)	7.60 × 10^−3^	
***DMRTA1***			**Combined**			**1.19 (1.13–1.26)**	**1.50 × 10**^**−9**^	**0.62**
rs9309245	**G**	C	Stage-1, set-1	0.178	0.164	1.10 (1.04–1.17)	1.57 × 10^−3^	
2			Stage-1, set-2	0.186	0.168	1.14 (1.08–1.20)	3.43 × 10^−6^	
53397048			Stage-2	0.181	0.172	1.06 (0.998–1.14)	5.95 × 10^−2^	
***ASB3***			**Combined**			**1.10 (1.07–1.14)**	**1.25 × 10**^**−8**^	**0.30**
rs67156297	**A**	G	Stage-1, set-1	0.102	0.086	1.21 (1.12–1.30)	1.28 × 10^−6^	
1			Stage-1, set-2	0.097	0.087	1.13 (1.05–1.21)	1.34 × 10^−3^	
154336716			Stage-2	0.092	0.087	1.07 (0.98–1.16)	1.33 × 10^−1^	
***ATP8B2***			**Combined**			**1.14 (1.09–1.19)**	**1.95 × 10**^**−8**^	**0.10**
rs7107784	**G**	A	Stage-1, set-1	0.099	0.089	1.16 (1.07–1.26)	4.91 × 10^−4^	
11			Stage-1, set-2	0.101	0.090	1.18 (1.09–1.27)	3.88 × 10^−5^	
2215089			Stage-2	0.093	0.086	1.09 (0.998–1.19)	5.58 × 10^−2^	
***MIR4686***			**Combined**			**1.14 (1.09–1.20)**	**2.07 × 10**^**−8**^	**0.42**
rs67839313	**C**	T	Stage-1, set-1	0.280	0.261	1.10 (1.05–1.16)	7.87 × 10^−5^	
15			Stage-1, set-2	0.278	0.264	1.08 (1.03–1.14)	1.75 × 10^−3^	
40619724			Stage-2	0.281	0.267	1.07 (1.01–1.13)	1.36 × 10^−2^	
***INAFM2***			**Combined**			**1.09 (1.06–1.12)**	**2.42 × 10**^**−8**^	**0.73**

CI, confidence interval; Chr., chromosome; OR, odds ratio; *P*het, *P*-value for Cochran's *Q*-test for heterogeneity; RAF, risk allele frequency; SNP, single-nucleotide polymorphism.

Alleles are aligned to the forward strand of NCBI Build 37.

**Table 2:** Association of novel seven SNP loci with type 2 diabetes risk in the population other than Japanese.

**SNP ID**	**Alleles**			**Population**	**OR (95% CI)**	***P*****-value**	***P*****het**	RAF*
	**Risk**	**Other**						
rs1116357	G	A	All replication set†		1.01 (0.99–1.03)	1.99 × 10^−1^	0.96	
*CCDC85A*				East Asian	1.03 (0.99–1.07)	2.06 × 10^−1^	0.98	0.28
				South Asian	1.02 (0.98–1.05)	4.18 × 10^−1^	0.34	0.57
				European	1.01 (0.98–1.04)	5.45 × 10^−1^	0.98	0.53
				Mexican/Latino	0.99 (0.93–1.05)	5.96 × 10^−1^	NA	0.48
			All Japanese		1.09 (1.06–1.12)	6.97 × 10^−10^	0.21	0.28
rs147538848	A	G	All replication set		1.10 (1.05–1.16)	2.25 × 10^−4^	0.82	
*FAM60A*				East Asian	1.13 (1.05–1.20)	3.58 × 10^−4^	0.88	0.19
				South Asian	1.07 (0.97–1.17)	1.59 × 10^−1^	0.35	0.06
				European	NA	NA	NA	0.01
				Mexican/Latino	1.01 (0.74–1.37)	8.98 × 10^−1^	NA	0.01
			All Japanese		1.11 (1.07–1.15)	7.83 × 10^−10^	0.99	0.23
rs1575972	T	A	All replication set		1.13 (1.08–1.18)	2.26 × 10^−7^	0.13	
*DMRTA1*				East Asian	1.14 (1.02–1.26)	1.62 × 10^−2^	0.03	0.96
				South Asian	1.13 (1.03–1.24)	1.21 × 10^−2^	0.45	0.94
				European	1.14 (1.07–1.21)	5.58 × 10^−5^	0.56	0.97
				Mexican/Latino	1.004 (0.83–1.21)	9.92 × 10^−1^	NA	0.98
			All Japanese		1.19 (1.13–1.26)	1.50 × 10^−9^	0.62	0.93
rs9309245	G	C	All replication set		1.01 (0.99–1.03)	5.50 × 10^−1^	0.58	
*ASB3*				East Asian	1.04 (0.99–1.09)	1.64 × 10^−1^	0.40	0.20
				South Asian	0.996 (0.96–1.04)	8.21 × 10^−1^	0.63	0.31
				European	1.01 (0.98–1.04)	4.74 × 10^−1^	0.80	0.35
				Mexican/Latino	0.96 (0.90–1.03)	2.31 × 10^−1^	NA	0.26
			All Japanese		1.10 (1.07–1.14)	1.25 × 10^−8^	0.30	0.17
rs67156297	A	G	All replication set		1.03 (1.001–1.05)	4.08 × 10^−2^	0.38	
*ATP8B2*				East Asian	1.05 (0.99–1.12)	1.22 × 10^−1^	0.26	0.09
				South Asian	0.98 (0.94–1.03)	4.79 × 10^−1^	0.99	0.20
				European	1.04 (0.999–1.08)	5.90 × 10^−2^	0.88	0.25
				Mexican/Latino	1.08 (0.995–1.18)	8.19 × 10^−2^	NA	0.16
			All Japanese		1.14 (1.09–1.19)	1.95 × 10^−8^	0.10	0.10
rs7107784	G	A	All replication set		1.05 (1.03–1.08)	7.05 × 10^−7^	0.0079	
*MIR4686*				East Asian	1.001 (0.91–1.10)	9.83 × 10^−1^	0.010	0.12
				South Asian	1.09 (1.03–1.15)	1.60 × 10^−3^	0.20	0.25
				European	1.04 (1.02–1.07)	4.39 × 10^−4^	0.43	0.28
				Mexican/Latino	1.15 (1.05–1.25)	6.00 × 10^−4^	NA	0.16
			All Japanese		1.14 (1.09–1.20)	2.07 × 10^−8^	0.42	0.08
rs67839313	C	T	All replication set		1.05 (1.02–1.07)	2.91 × 10^−4^	0.41	
*INAFM2*				East Asian	1.09 (1.04–1.15)	1.93 × 10^−4^	0.50	0.25
				South Asian	1.03 (0.99–1.08)	1.68 × 10^−1^	0.77	0.32
				European	1.02 (0.98–1.06)	3.61 × 10^−1^	0.18	0.11
				Mexican/Latino	1.06 (0.97–1.14)	1.16 × 10^−1^	NA	0.19
			All Japanese		1.09 (1.06–1.12)	2.42 × 10^−8^	0.73	0.27

CI, confidence interval; OR, odds ratio; *P*het, *P*-value for Cochran's *Q*-test for heterogeneity; RAF, risk allele frequency; SNP, single-nucleotide polymorphism.

Alleles are aligned to the forward strand of NCBI Build 37.

^*^Risk allele frequency in each populations. Data are from 1000 Genomes Project Phase 3 allele frequencies (ASN, SAS, EUR, MXL and JPT).

^†^Conbined association data from 21 studies (11 East Asian other than Japanese, 6 South Asian, 3 European and one Mexican/Latino).
