# Supplementary material for: Genome-wide association studies in the Japanese population identify seven novel loci for type 2 diabetes
Source: Nat Commun. 2016 Jan 28;7:10531. doi: 10.1038/ncomms10531 (PMC4738362; doi:10.1038/ncomms10531)
Supplement: Supplementary Information — Supplementary Figures 1-8, Supplementary Tables 1-27, Supplementary Note and Supplementary References [file ncomms10531-s1.pdf]

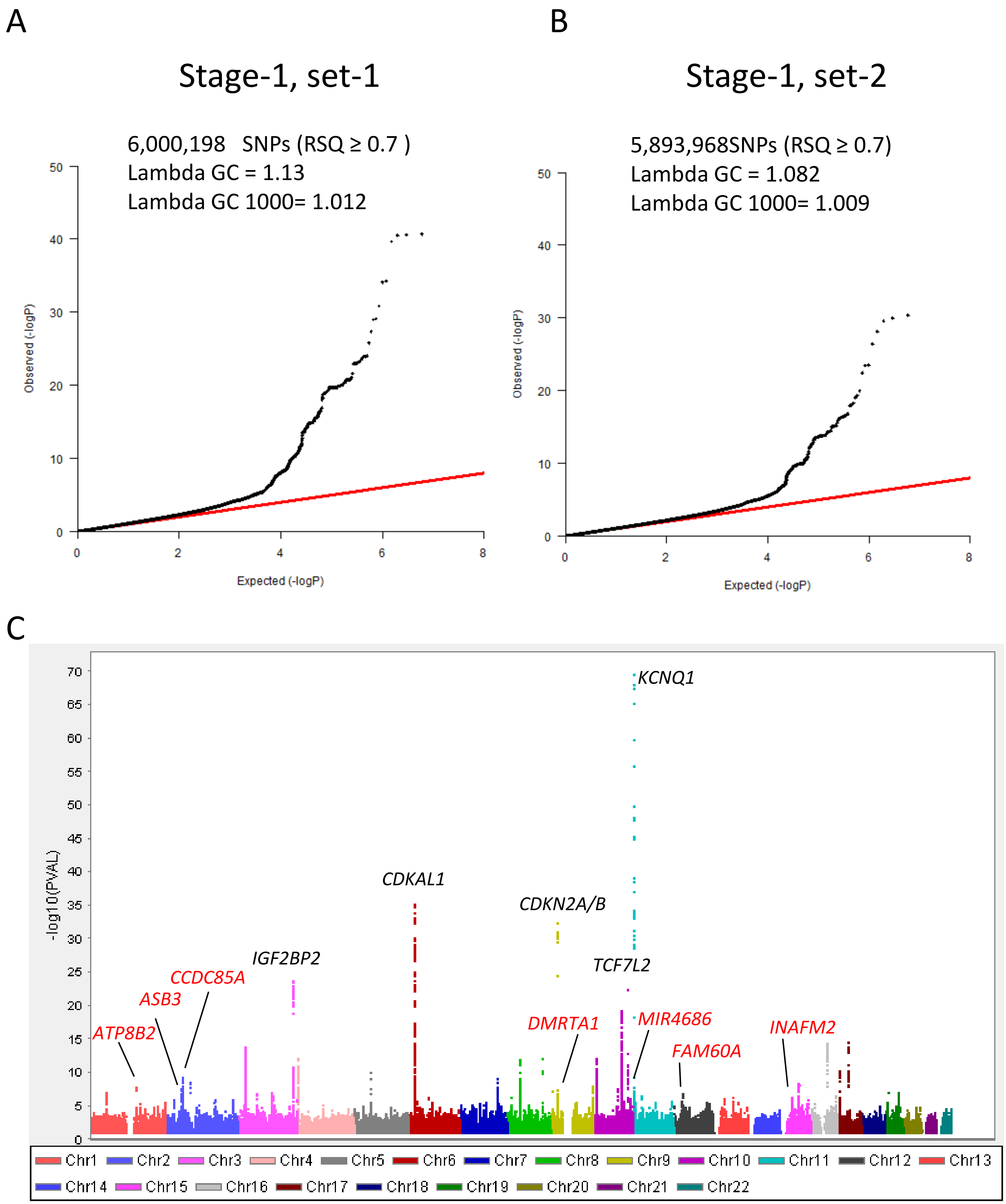

Supplementary Figure 1. QQ plots and Manhattan plot in the discovery stage.
A.QQ plot for the Stage-1, set-1 GWAS. Association p-values on imputed genotype data for 6,000,198 SNPs (RSQ ≥ 0.7) were plotted. **B. QQ plot for the Stage-1, set-2 GWAS**. Association p-values on imputed genotype data for 5,893,968 SNPs (RSQ ≥ 0.7) are plotted. **C. Manhattan plot from the GWAS meta-analysis (set-1 + set-2)**. The seven novel loci are indicated in red type.

Supplementary Figure 2

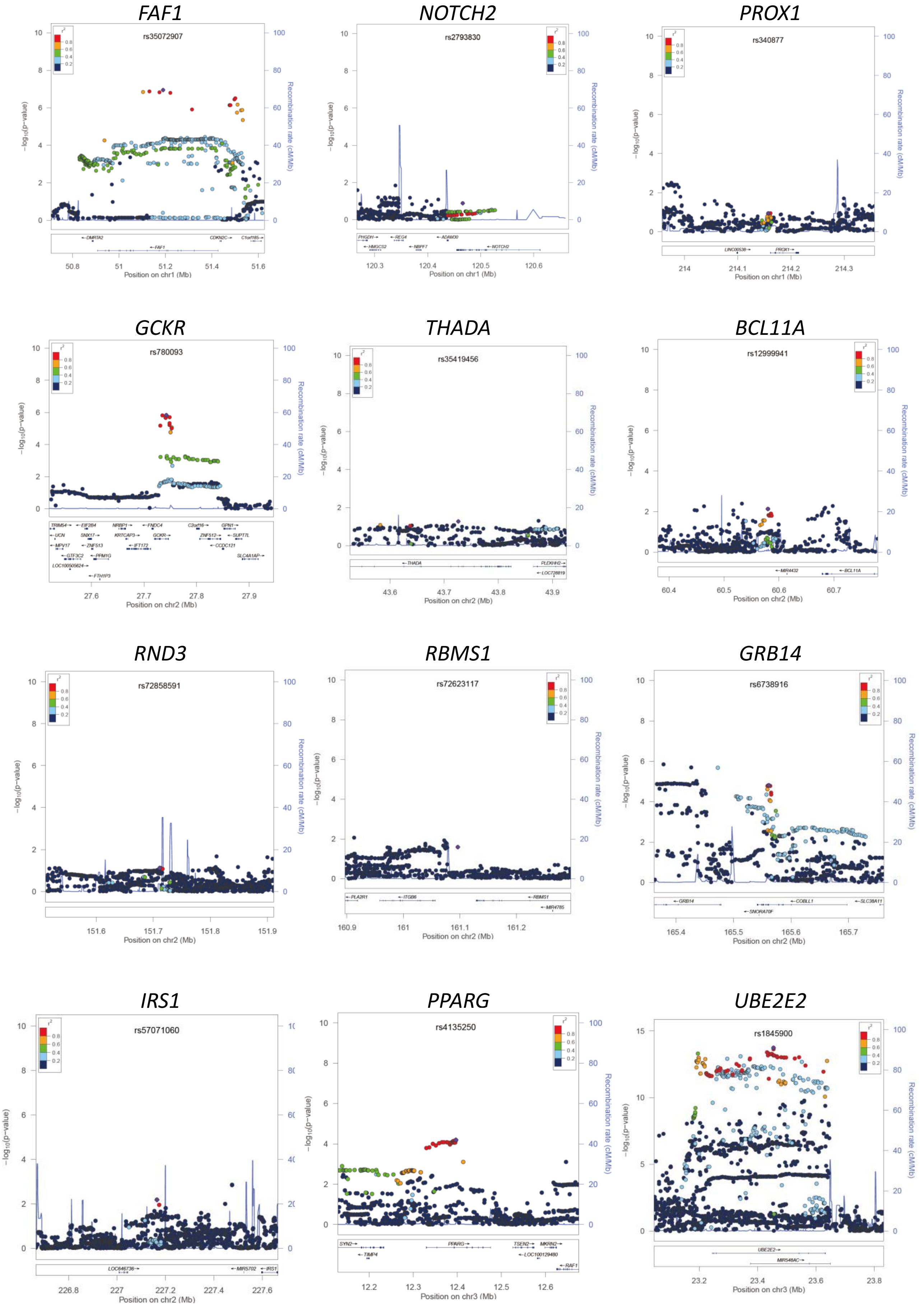

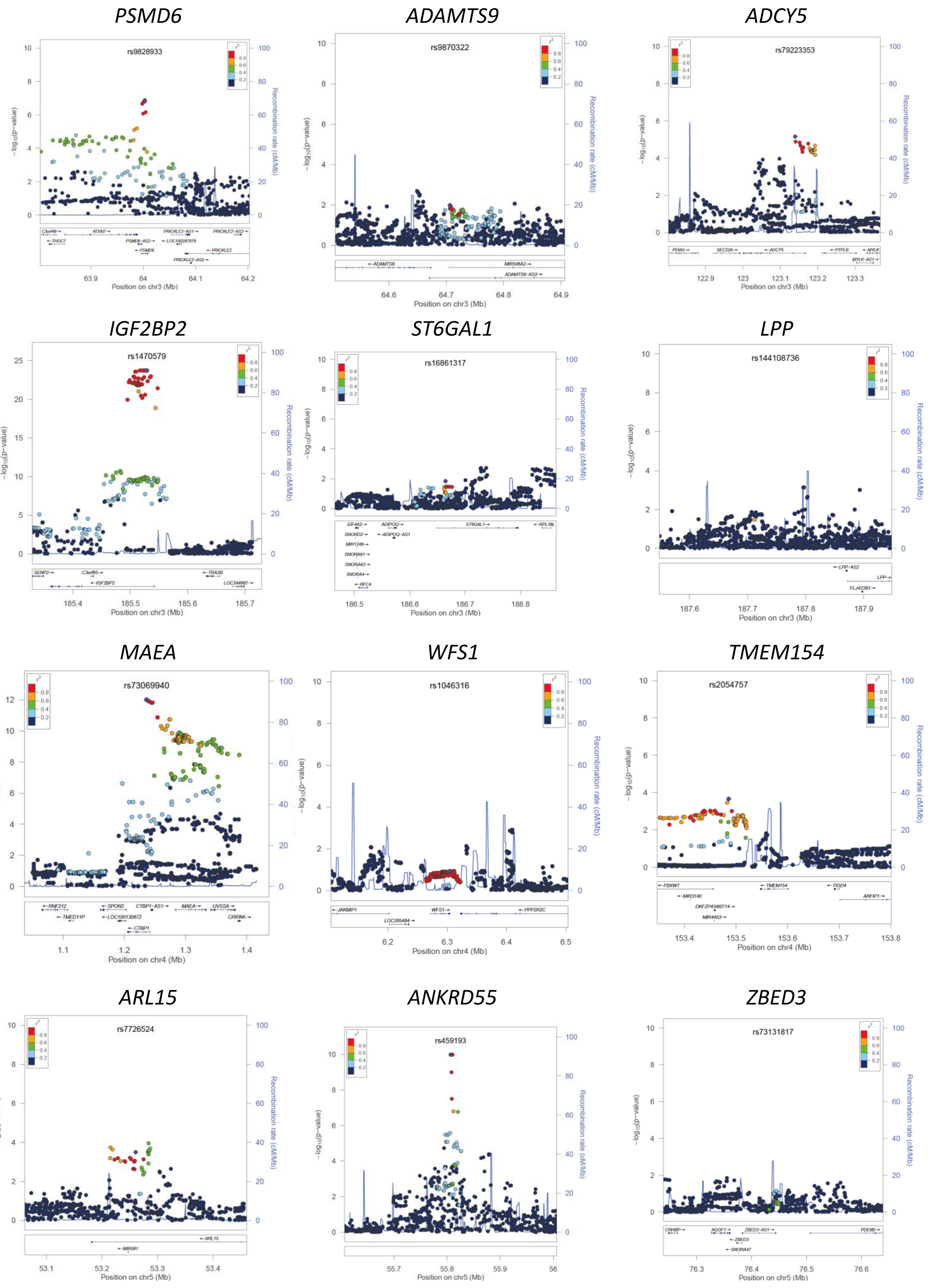
PSMD6
rs9828933
Position on chr3 (Mb)
ADAMTS9
rs9870322
Position on chr3 (Mb)
ADCY5
rs79223353
Position on chr3 (Mb)
IGF2BP2
rs1470579
Position on chr3 (Mb)
ST6GAL1
rs16861317
Position on chr3 (Mb)
LPP
rs144108736
Position on chr3 (Mb)
MAEA
rs73069940
Position on chr4 (Mb)
WFS1
rs1046316
Position on chr4 (Mb)
TMEM154
rs2054757
Position on chr4 (Mb)
ARL15
rs7726524
Position on chr5 (Mb)
ANKRD55
rs459193
Position on chr5 (Mb)
ZBED3
rs73131817
Position on chr5 (Mb)
−log10(p−value)
Recombination rate (cM/Mb)

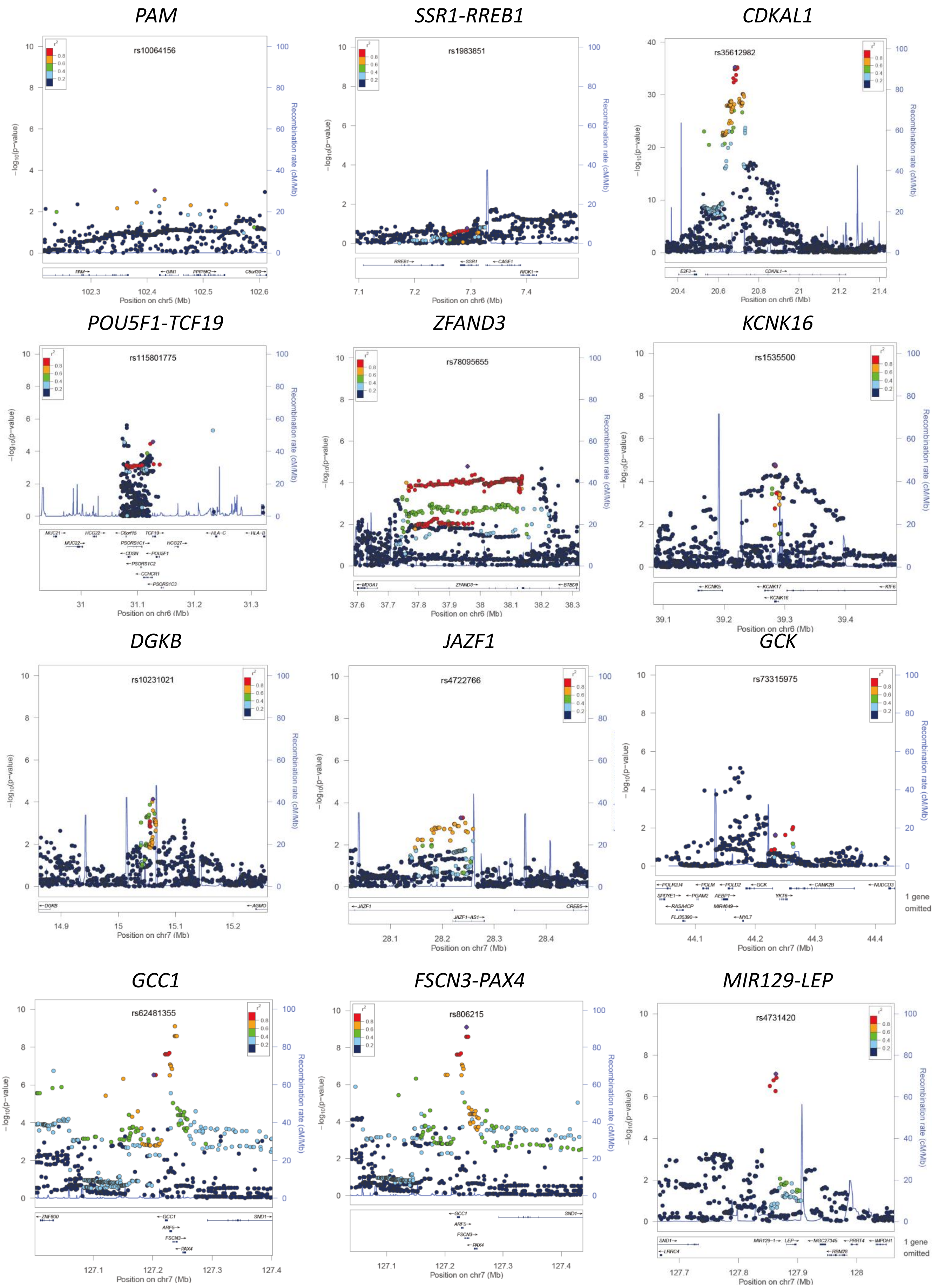
PAM
rs10064156
Position on chr5 (Mb)
SSR1-RREB1
rs1983851
Position on chr6 (Mb)
CDKAL1
rs35612982
Position on chr6 (Mb)
POU5F1-TCF19
rs115801775
Position on chr6 (Mb)
ZFAND3
rs78095655
Position on chr6 (Mb)
KCNK16
rs1535500
Position on chr6 (Mb)
DGKB
rs10231021
Position on chr7 (Mb)
JAZF1
rs4722766
Position on chr7 (Mb)
GCK
rs73315975
Position on chr7 (Mb)
1 gene omitted
GCC1
rs62481355
Position on chr7 (Mb)
FSCN3-PAX4
rs806215
Position on chr7 (Mb)
MIR129-LEP
rs4731420
Position on chr7 (Mb)
1 gene omitted
−log10(p−value)
Recombination rate (cM/Mb)
r2

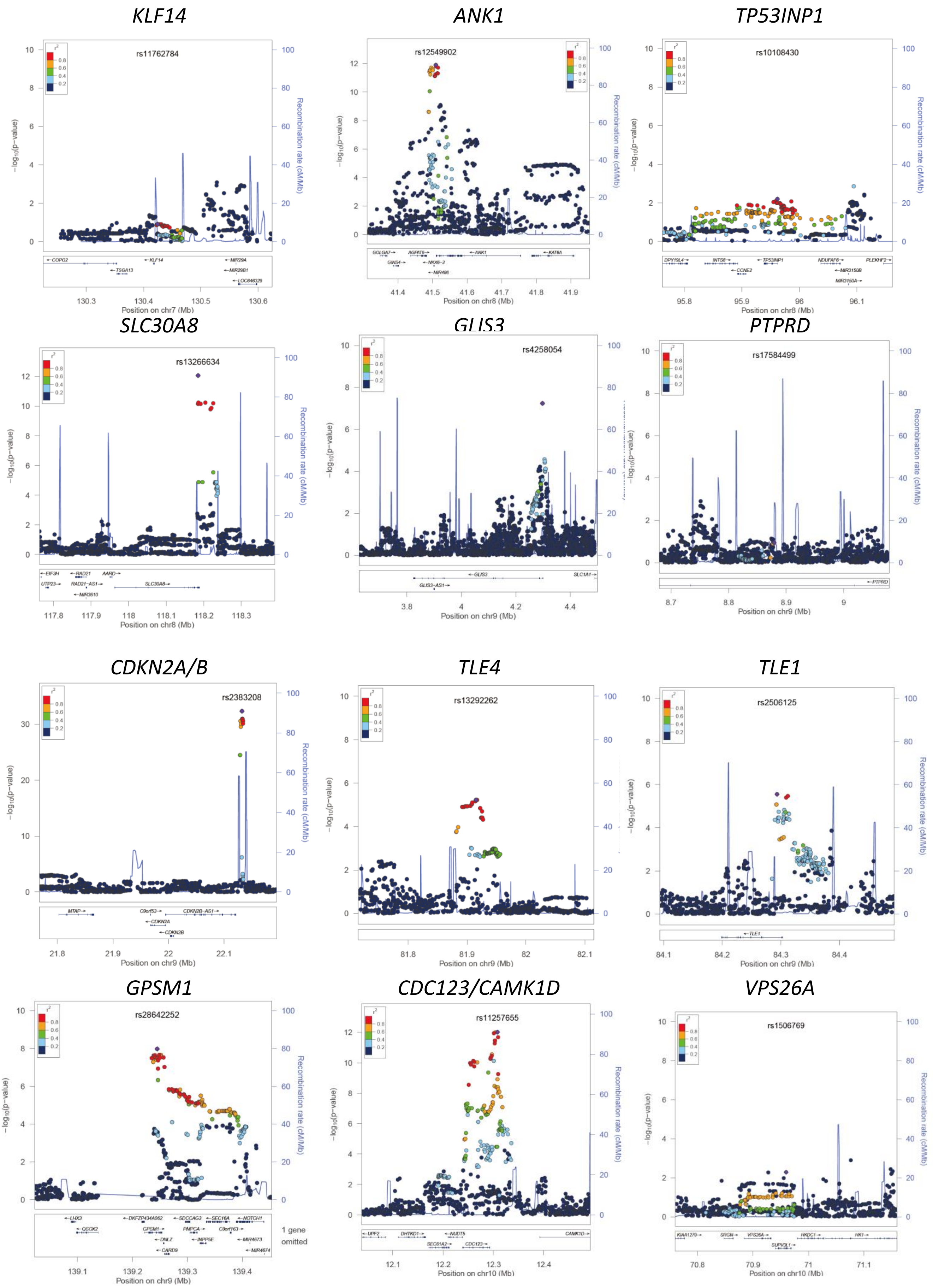
KLF14
rs11762784
ANK1
rs12549902
TP53INP1
rs10108430
SLC30A8
rs13266634
GLIS3
rs4258054
PTPRD
rs17584499
CDKN2A/B
rs2383208
TLE4
rs13292262
TLE1
rs2506125
GPSM1
rs28642252
CDC123/CAMK1D
rs11257655
VPS26A
rs1506769
−log10(p−value)
Recombination rate (cM/Mb)
Position on chr7 (Mb)
Position on chr8 (Mb)
Position on chr9 (Mb)
Position on chr10 (Mb)
1 gene omitted

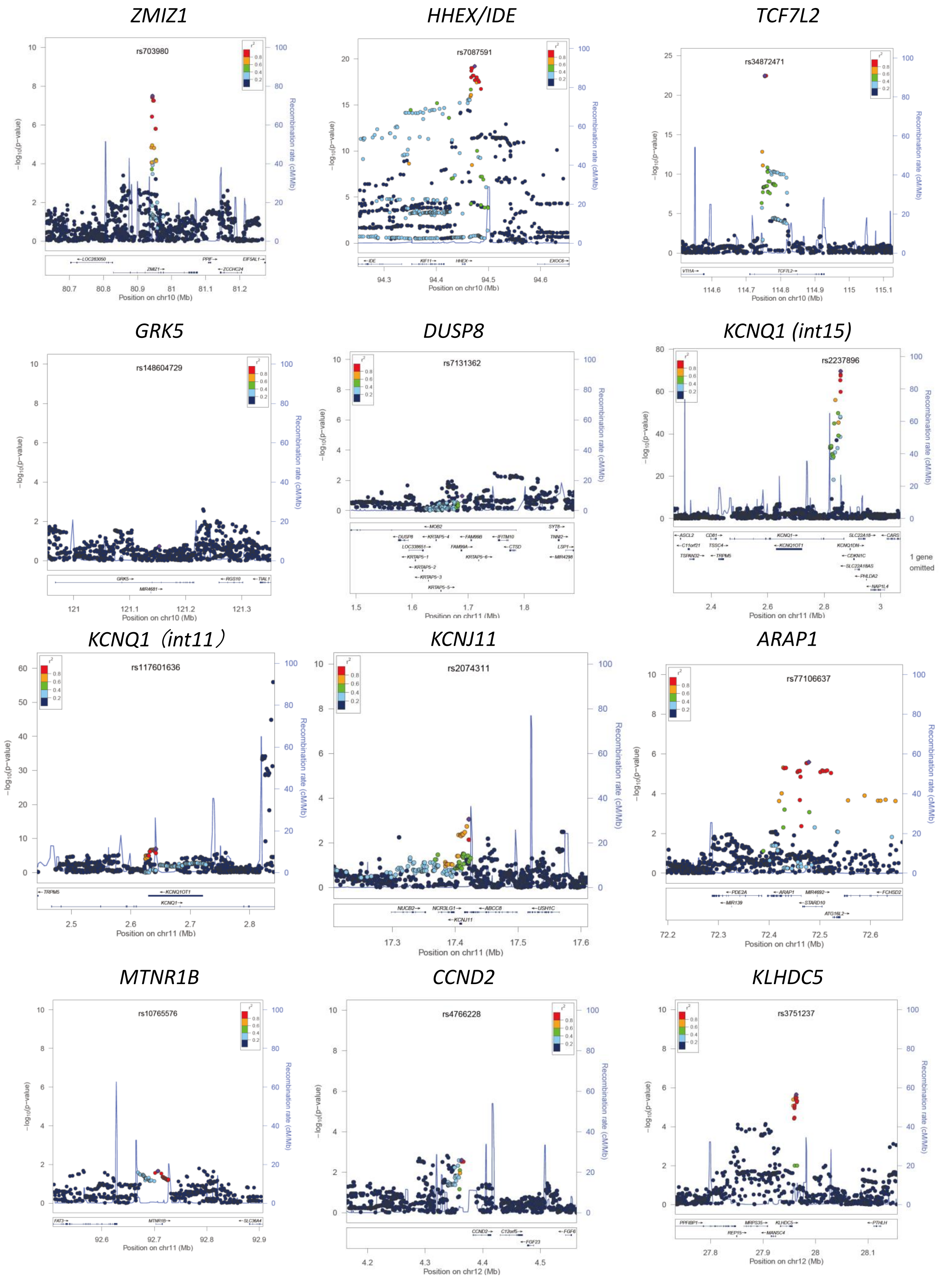

ZMIZ1
rs703980
Position on chr10 (Mb)
HHEX/IDE
rs7087591
Position on chr10 (Mb)
TCF7L2
rs34872471
Position on chr10 (Mb)
GRK5
rs148604729
Position on chr10 (Mb)
DUSP8
rs7131362
Position on chr11 (Mb)
KCNQ1 (int15)
rs2237896
1 gene omitted
Position on chr11 (Mb)
KCNQ1（int11）
rs117601636
Position on chr11 (Mb)
KCNJ11
rs2074311
Position on chr11 (Mb)
ARAP1
rs77106637
Position on chr11 (Mb)
MTNR1B
rs10765576
Position on chr11 (Mb)
CCND2
rs4766228
Position on chr12 (Mb)
KLHDC5
rs3751237
Position on chr12 (Mb)
−log10(p−value)
Recombination rate (cM/Mb)

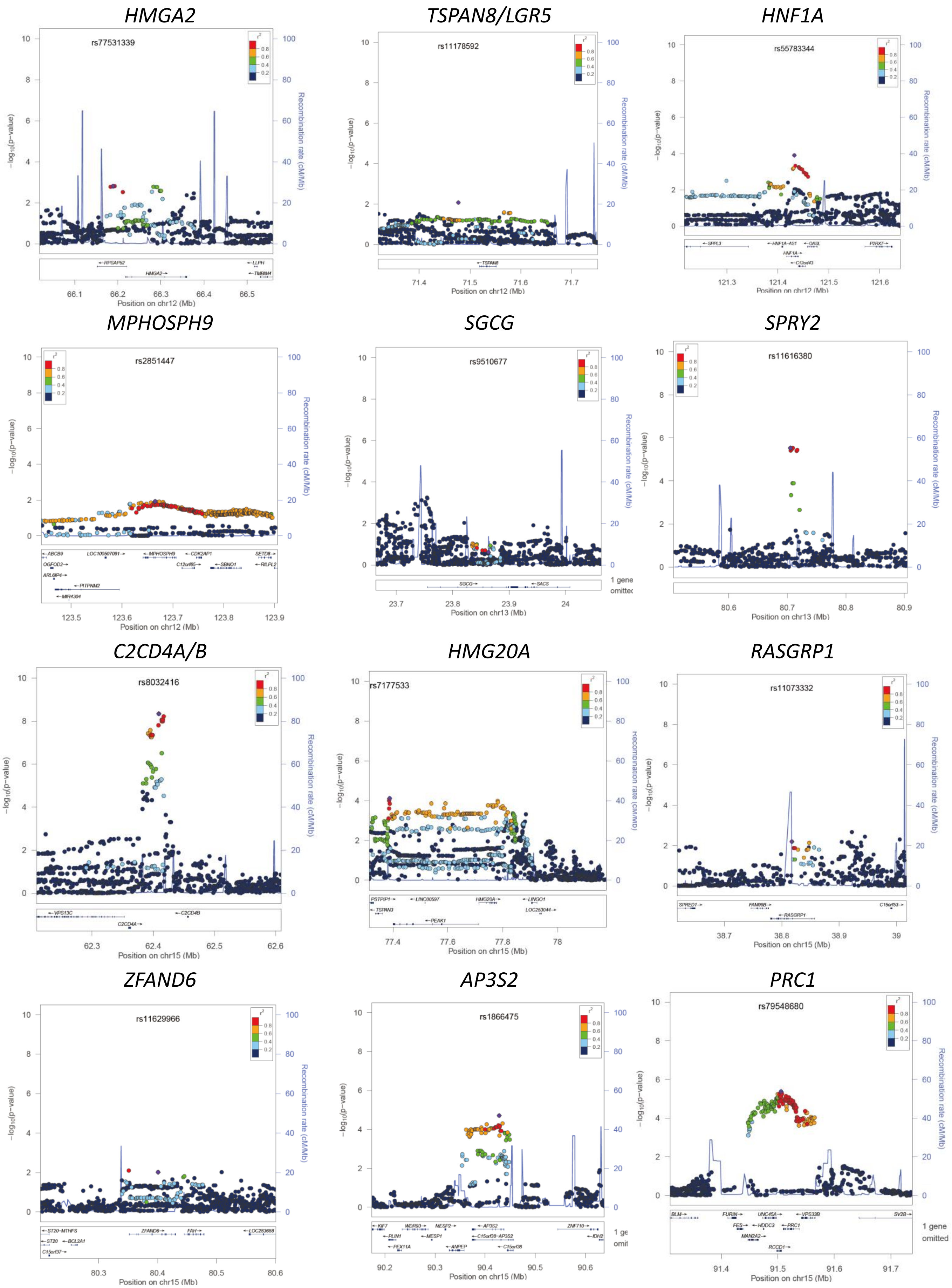
HMGA2
rs77531339
Position on chr12 (Mb)
TSPAN8/LGR5
rs11178592
Position on chr12 (Mb)
HNF1A
rs55783344
Position on chr12 (Mb)
MPHOSPH9
rs2851447
Position on chr12 (Mb)
SGCG
rs9510677
Position on chr13 (Mb)
SPRY2
rs11616380
Position on chr13 (Mb)
C2CD4A/B
rs8032416
Position on chr15 (Mb)
HMG20A
rs7177533
Position on chr15 (Mb)
RASGRP1
rs11073332
Position on chr15 (Mb)
ZFAND6
rs11629966
Position on chr15 (Mb)
AP3S2
rs1866475
Position on chr15 (Mb)
PRC1
rs79548680
Position on chr15 (Mb)
−log10(p−value)
Recombination rate (cM/Mb)

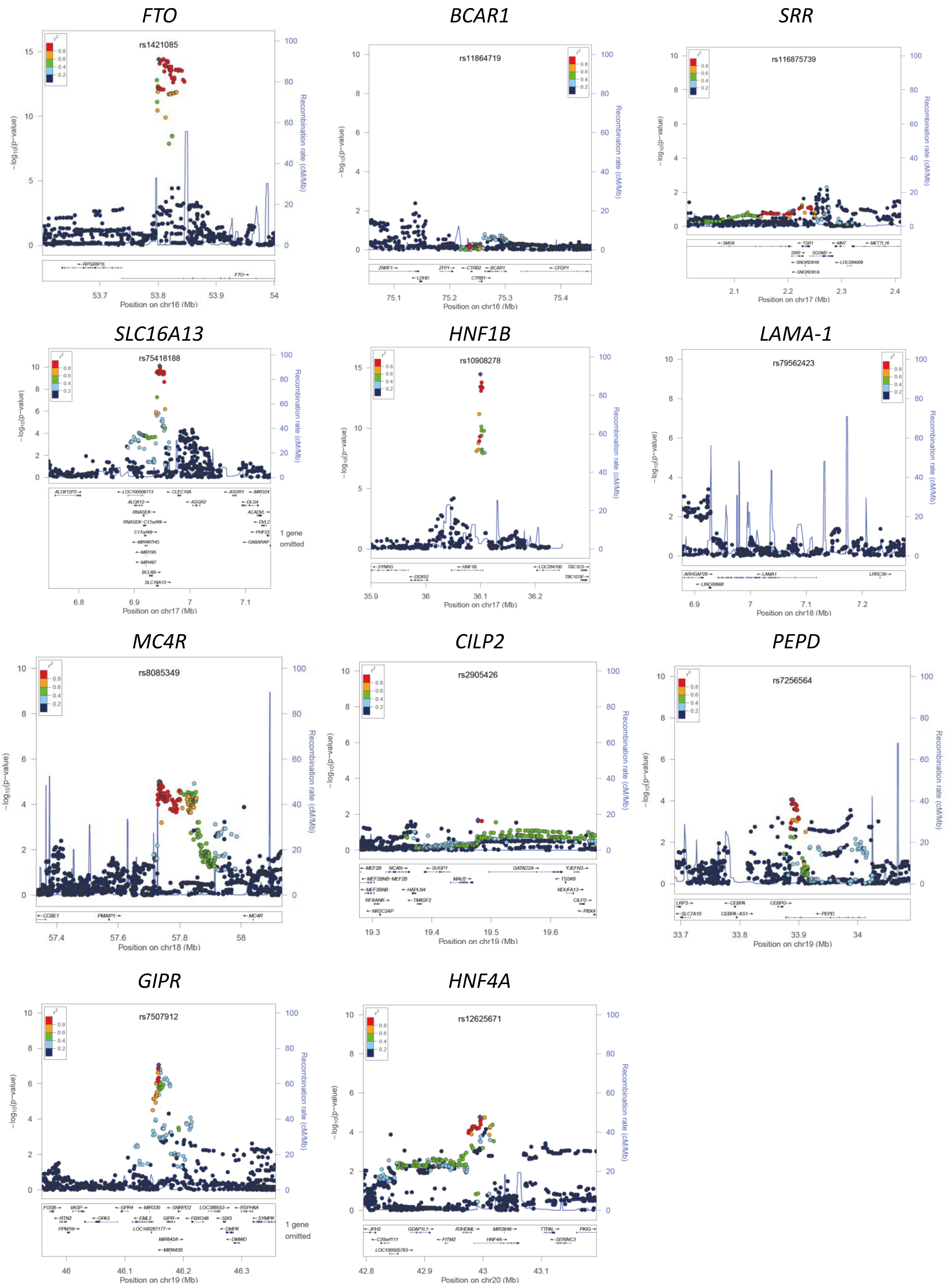

FTO
rs1421085
Position on chr16 (Mb)
BCAR1
rs11864719
Position on chr16 (Mb)
SRR
rs116875739
Position on chr17 (Mb)
SLC16A13
rs75418188
1 gene omitted
Position on chr17 (Mb)
HNF1B
rs10908278
Position on chr17 (Mb)
LAMA-1
rs79562423
Position on chr18 (Mb)
MC4R
rs8085349
Position on chr18 (Mb)
CILP2
rs2905426
Position on chr19 (Mb)
PEPD
rs7256564
Position on chr19 (Mb)
GIPR
rs7507912
1 gene omitted
Position on chr19 (Mb)
HNF4A
rs12625671
Position on chr20 (Mb)
−log10(p−value)
Recombination rate (cM/Mb)

Supplementary Figure 2. Regional association plots of the discovery stage GWAS meta-analysis (Stage-1: set-1 and set-2) for 83 established T2D susceptibility loci. Each plot shows $-\log_{10}$ *P* values against the chromosomal positions of SNPs in the specific region. The SNP with the strongest association signal (lead SNP) in each locus is represented as a purple diamond; the other SNPs are colored according to the extent of LD with the lead SNP. Estimated recombination rates from the hg19/1000 Genomes Project March 2012 East Asian reference are shown as light-blue lines

Supplementary Figure 3

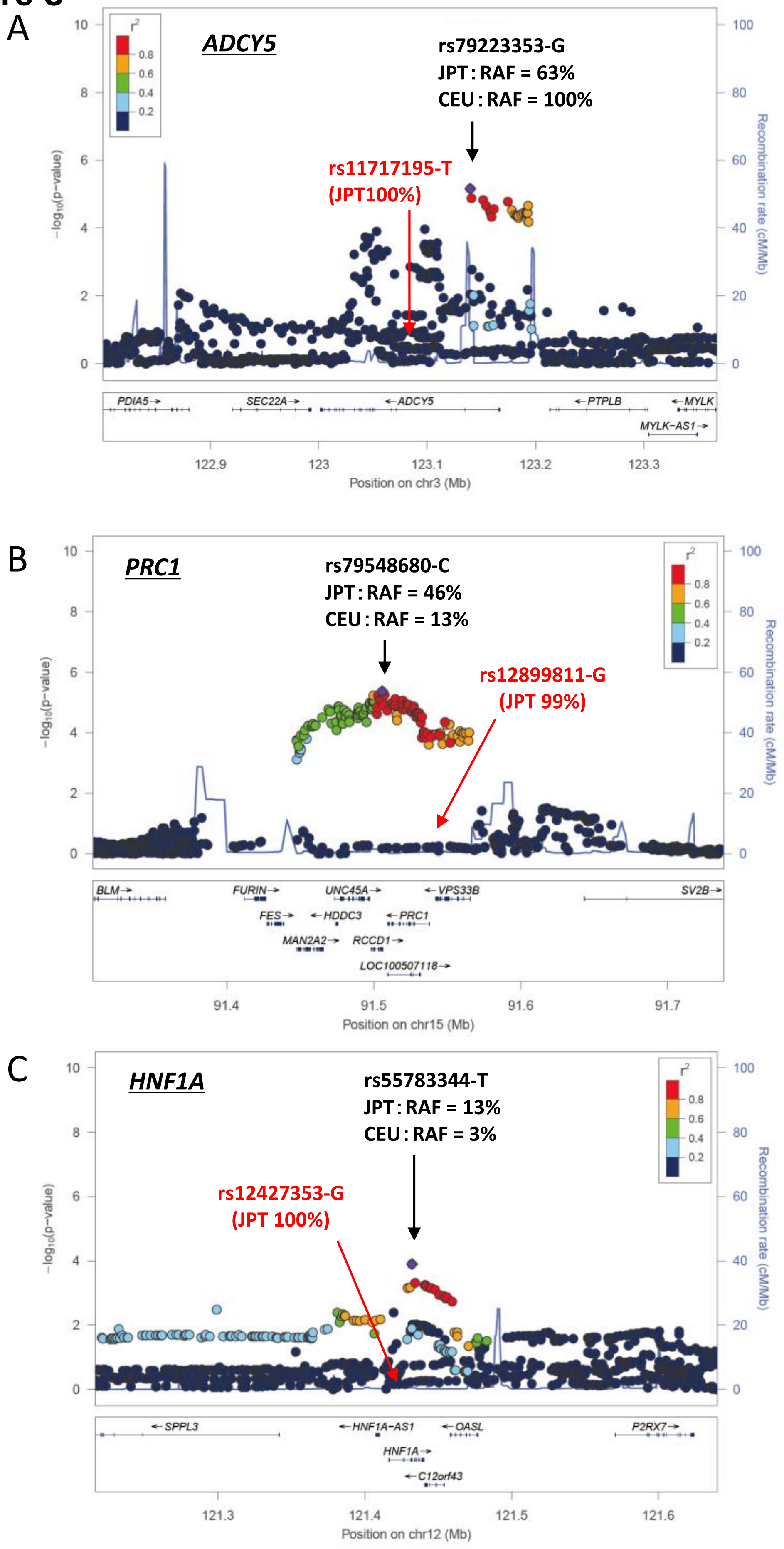

Supplementary Figure 3. Regional association plots for *ADCY5*, *HNF1A* and *PRC1* in the discovery stage GWAS meta-analysis (Stage-1:set-1 and set-2) Each plot shows $-\log_{10} P$ values against the chromosomal positions of SNPs in the specific region. The SNP with the strongest association signal in each locus is represented as a purple diamond; the other SNPs are colored according to the extent of LD with this SNP. Estimated recombination rates from the hg19/1000 Genomes Project March 2012 East Asian reference are shown as light-blue lines.

Supplementary Figure 4. Genes prioritized by PPI network

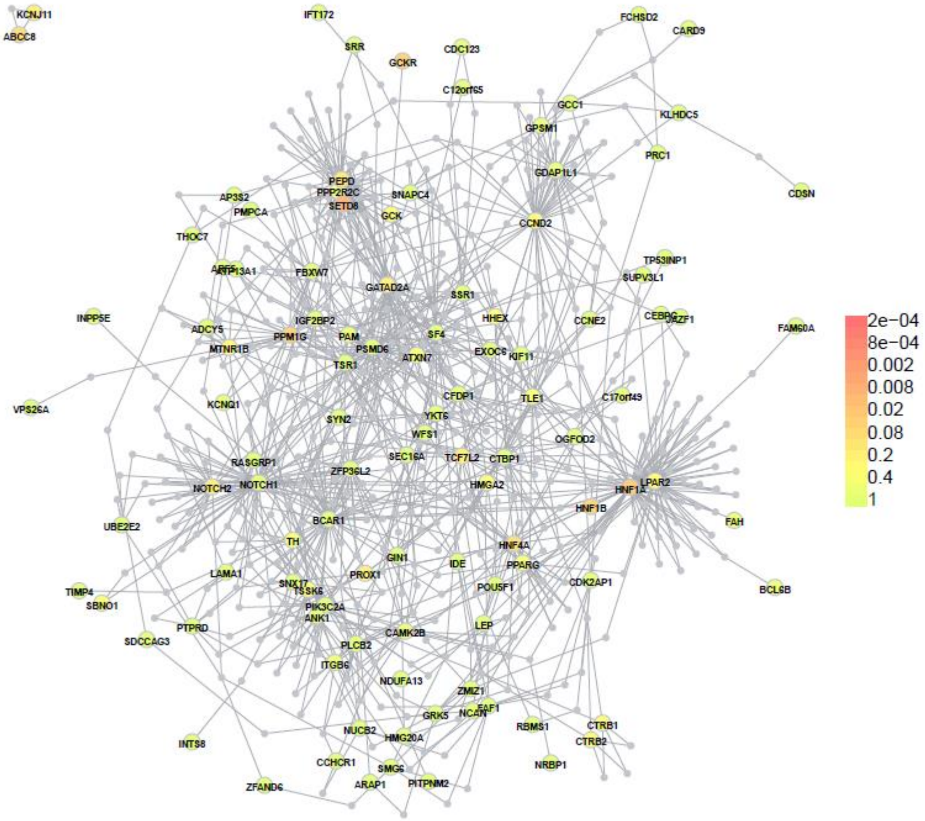

GENE	P_corrected
SETD8	**0.006299**
HNF1A	**0.014667**
GCKR	**0.025077**
HNF1B	**0.029226**
PPM1G	**0.043676**
HNF4A	0.066165
ABCC8	0.066165
PEPD	0.088387
GATAD2A	0.100395
KCNJ11	0.106369
PROX1	0.132027
TCF7L2	0.155379
NOTCH2	0.172684
PPP2R2C	0.203007
CCND2	0.20863
MTNR1B	0.20863
HMGA2	0.229075
GCK	0.236444
HHEX	0.252893
TSSK6	0.26376

We used DAPPLE ver2.0 (http://www.broadinstitute.org/mpg/dapple/dappleTMP.php) to identify genes prioritized by PPI network. We entered 90 T2D regions defined in the supplementary method section (±25 kb or r^2>0.50) as inputs and defined the number for cutoff of Common Interactor binding degree as 2. We defined the genes with corrected $p < 0.05$ as PPI network genes.

A

Biological T2D risk gene prioritization criteria

1) T2D risk missense variant (n = 20)
2) Monogenic diabetes (n = 11)
3) Protein-protein interaction (n = 5)
4) knockout mouse phenotype (n = 46)
5) Pubmed text mining (n = 30)
6) cis-eQTL (n = 55)

B

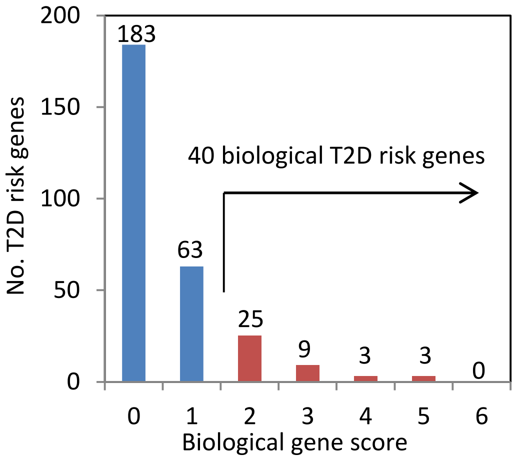

C

Correlation of prioritization criteria of biological genes from T2D risk loci (R^2)	T2D risk missense variant	Monogenic diabetes	Protein-protein interaction	knockout mouse phenotype	Pubmed text mining	cis-eQTL
T2D risk missense variant	-	0.0531	0.0046	0.0045	0.0482	0.0016
Monogenic diabetes	0.0531	-	0.0629	0.166	0.3414	0.0076
Protein-protein interaction	0.0046	0.0629	-	0.0255	0.0465	0.005
knockout mouse phenotype	0.0045	0.166	0.0255	-	0.2224	0.0223
Pubmed text mining	0.0482	0.3414	0.0465	0.2224	-	0.0042
cis-eQTL	0.0016	0.0076	0.005	0.0223	0.0042	-

Supplementary Figure 5. Prioritization of biological candidate genes from T2D risk loci. **A**, Prioritization criteria of biological candidate genes from T2D risk loci. **B**, Histogram distribution of gene scores. The 40 genes with Score ≥ 2 (red) were defined as 'biological T2D risk genes'. **C**, Correlations between every 2 biological candidate gene prioritization criteria.

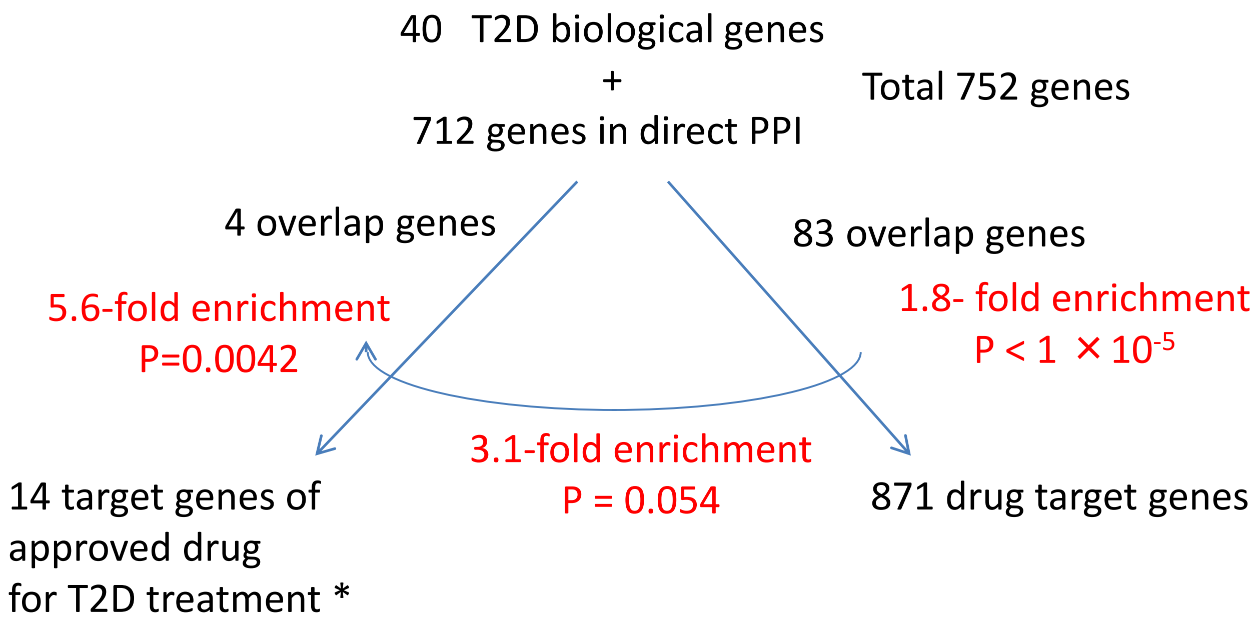

Supplementary Figure 6. Overlap enrichment analysis

Overlap and relative enrichment between the biological T2D risk genes or genes in direct PPI with the biological T2D risk genes and the drug target genes was assessed by permutation procedure, as previously reported by Okada Y et al (*Nature* **20**, 376-381,2014) .

* See supplementary Table 22

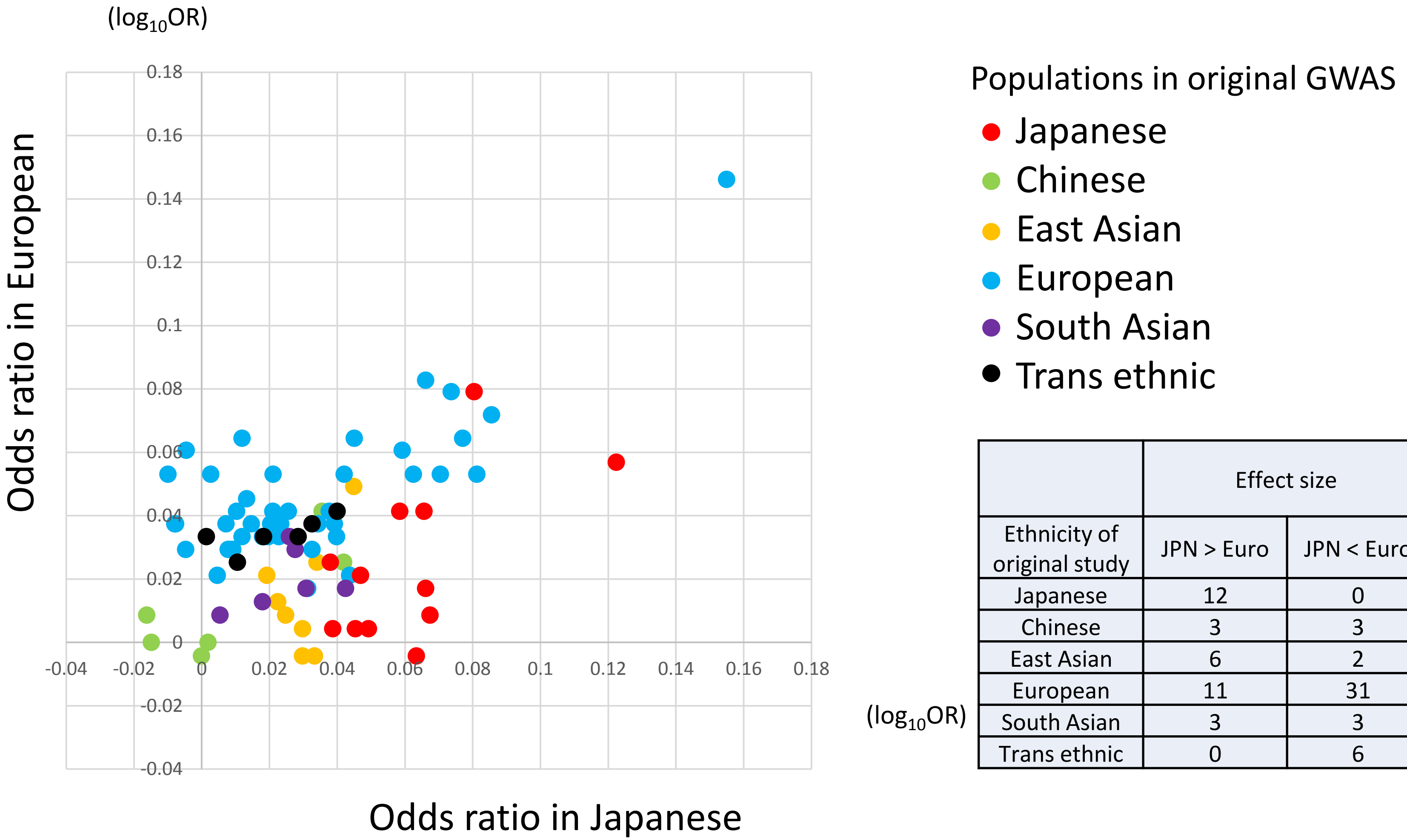

	Effect size	
Ethnicity of original study	JPN > Euro	JPN < Euro
Japanese	12	0
Chinese	3	3
East Asian	6	2
European	11	31
South Asian	3	3
Trans ethnic	0	6

Supplementary Figure 7 :Effect sizes for established and novel T2D loci; direct comparison between Japanese and European populations

Effect sizes in Japanese GWAS data (GWAS set1+set2: 14,463 cases and 26, 183 controls) and European data (DIAGRAM3 12,171 cases and 56,862 controls http://diagram-consortium.org/downloads.html) are shown across 6 novel and 74 established T2D loci of which both of the Japanese and European association data are available. Lead SNPs in each locus in the population of original reports were selected.

Supplementary Figure 8. Systematic evaluation for effect sizes and LD in 60 loci identified in Japanese or European GWAS

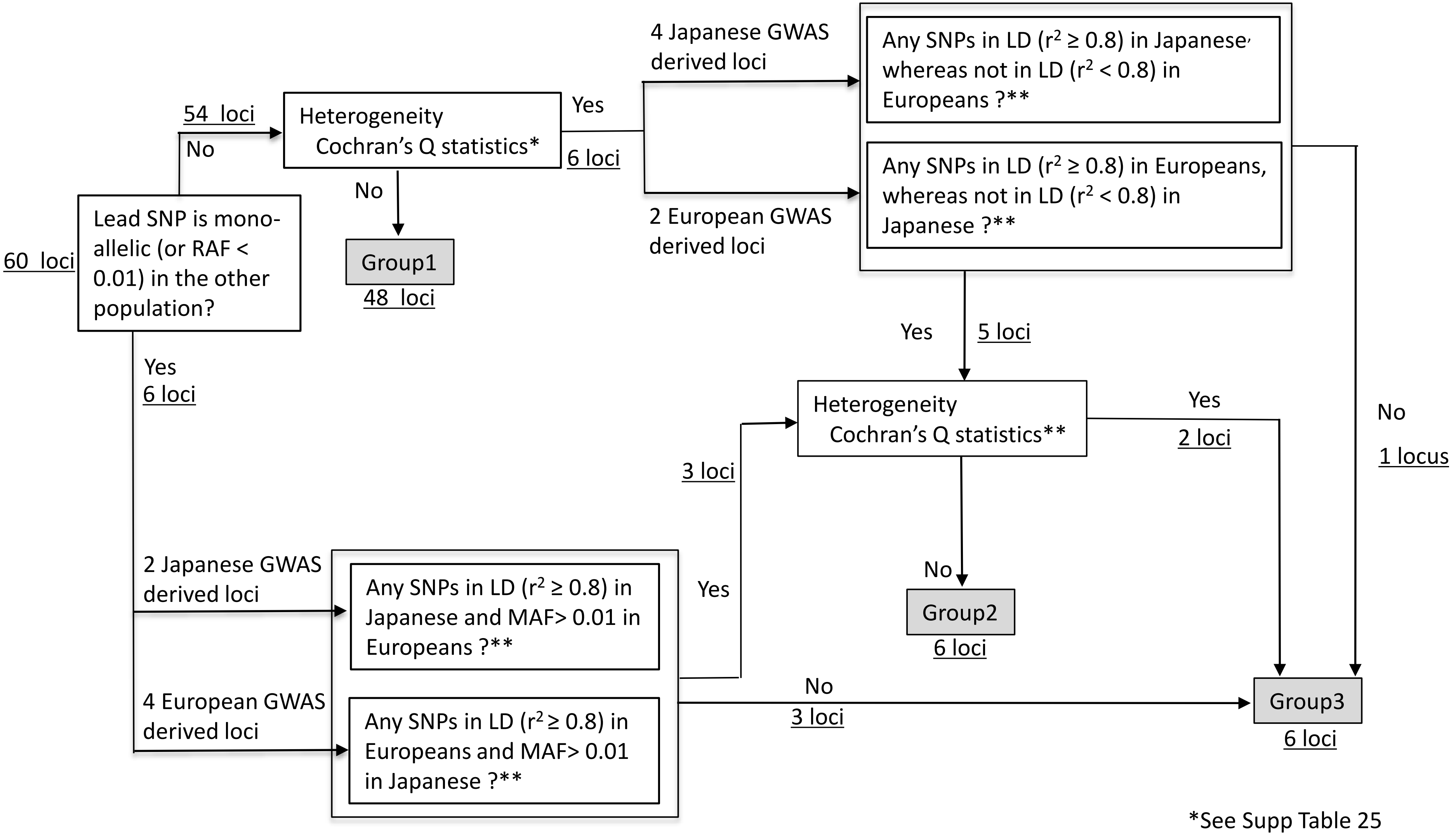

*See Supp Table 25

**See Supp Table 26

Supplementary Table 1. Twenty-five already established type 2 diabetes SNP loci with $p < 1 \times 10^{-6}$ in Stage-1 GWAS meta-analysis

locus	SNPID for lead SNP	chr	Chr.loc (Build 37 bp)	**Risk**	Non Risk	OR	95% CI	P for association	p for hetero
KCNQ1 (intron 15)	rs2237896	11	2,858,440	**G**	A	1.33	(1.28 - 1.37)	2.81.E-70	0.078
CDKAL1	rs35612982	6	20,682,622	**C**	T	1.21	(1.17 - 1.25)	5.91.E-36	0.237
CDKN2A/B	rs2383208	9	22,132,076	**A**	G	1.22	(1.18 - 1.26)	4.88.E-33	0.618
IGF2BP2	rs1470579	3	185,529,080	**C**	A	1.18	(1.14 - 1.21)	1.99.E-24	0.233
TCF7L2	rs34872471	10	114,754,071	**C**	T	1.43	(1.33 - 1.53)	3.47.E-23	0.901
HHEX	rs7087591	10	94,473,629	**G**	A	1.19	(1.14 - 1.23)	6.35.E-20	0.612
HNF1B	rs10908278	17	36,099,952	**T**	A	1.14	(1.1 - 1.18)	3.57.E-15	0.241
FTO	rs1421085	16	53,800,954	**C**	T	1.16	(1.12 - 1.21)	3.94.E-15	0.116
UBE2E2	rs1845900	3	23,454,565	**A**	G	1.18	(1.13 - 1.23)	1.87.E-14	0.594
SLC30A8	rs13266634	8	118,184,783	**C**	T	1.12	(1.09 - 1.16)	8.65.E-13	0.245
CDC123/CAMK1D	rs11257655	10	12,307,894	**T**	C	1.12	(1.08 - 1.15)	9.65.E-13	0.607
MAEA	rs73069940	4	1,236,502	**C**	G	1.13	(1.09 - 1.17)	1.00.E-12	0.86
ANK1	rs12549902	8	41,509,259	**A**	G	1.11	(1.08 - 1.15)	1.28.E-12	0.669
SLC16A13	rs75418188	17	6,945,483	**T**	C	1.2	(1.13 - 1.27)	7.89.E-11	0.9
ANKRD55	rs459193	5	55,806,751	**G**	A	1.11	(1.07 - 1.14)	1.04.E-10	0.115
FSCN3-PAX4	rs806215	7	127,237,312	**C**	T	1.11	(1.07 - 1.15)	7.79.E-10	0.444
C2CD4A/B	rs8032416	15	62,409,070	**G**	C	1.1	(1.06 - 1.13)	4.58.E-09	0.33
GPSM1	rs28642252	9	139,245,289	**G**	A	1.16	(1.1 - 1.22)	1.06.E-08	0.416
ZMIZ1	rs703980	10	80,943,841	**G**	A	1.09	(1.06 - 1.12)	3.23.E-08	0.139
GLIS3	rs4258054	9	4,297,892	**T**	C	1.1	(1.07 - 1.15)	5.76.E-08	0.978
MIR129-LEP	rs4731420	7	127,863,295	**C**	G	1.16	(1.1 - 1.22)	7.90.E-08	0.941
GIPR	rs7507912	19	46,157,916	**G**	A	1.11	(1.07 - 1.16)	8.61.E-08	0.266
KCNQ1 (intron 11)	rs117601636	11	2,642,037	**A**	G	1.16	(1.1 - 1.22)	1.14.E-07	0.421
FAF1	rs35072907	1	51,189,556	**C**	G	1.11	(1.07 - 1.15)	1.14.E-07	0.055
PSMD6	rs9828933	3	63,942,897	**T**	C	1.09	(1.06 - 1.13)	1.33.E-07	0.047

Chr:chromosome
Risk: risk allele in the present Japanese study.
non-risk: alternative allele in the present Japanese study.
OR: odds ratio
95%CI: 95% confidence interval
P-for hetero: p-values of Cochran's Q-test for heterogeneity

Supplementary Table 2. Seventeen suggestive SNP loci for type 2 diabetes with $p < 1 \times 10^{-6}$ in Stage-1 GWAS meta-analysis

nearby gene	chr	Chr,loc (Build 37 bp)	SNPID For lead SNP	**Risk**	non-risk	OR	95% CI	P-for association	P-for hetero	R^2 set-1	R^2 set-2
CCDC85A	2	57,287,411	rs1116357	**G**	A	1.11	(1.07 - 1.15)	6.88E-10	0.68	1.00	1.00
ATP8B2	1	154,336,716	rs67156297	**A**	G	1.16	(1.1 - 1.23)	1.44E-08	0.193	0.97	0.95
MIR4686	11	2,196,424	rs4930045	**T**	C	1.17	(1.11 - 1.23)	2.08E-08	0.734	0.78	0.76
ASB3	2	53,397,048	rs9309245	**G**	C	1.12	(1.08 - 1.17)	3.23E-08	0.407	1.00	1.00
DMRTA1	9	22,289,853	rs12000501	**A**	G	1.2	(1.13 - 1.29)	4.25E-08	0.361	0.92	0.88
SALL4P5	3	23,077,761	rs2688419	**T**	C	1.1	(1.06 - 1.14)	5.10E-08	0.795	0.91	0.92
FDX1L	19	10,423,876	rs187060802	**A**	G	1.4	(1.24 - 1.58)	8.10E-08	1.65E-08	0.97	0.93
ZBTB20	3	114,850,836	rs73230612	**T**	C	1.09	(1.06 - 1.12)	8.90E-08	0.148	0.99	1.00
FAM60A	12	31,466,613	rs147538848	**A**	G	1.11	(1.07 - 1.16)	1.88E-07	0.903	0.87	0.91
OR2BH1P	11	29,169,913	rs1965305	**T**	C	1.09	(1.05 - 1.12)	3.36E-07	0.306	0.98	0.99
C16orf74	16	85,735,652	rs377457	**A**	G	1.09	(1.05 - 1.13)	4.45E-07	0.564	0.99	0.89
MIR4704	13	66,805,708	rs4884660	**T**	C	1.1	(1.06 - 1.14)	4.65E-07	0.393	0.99	0.99
INAFM2	15	40,619,724	rs67839313	**C**	T	1.09	(1.06 - 1.13)	4.79E-07	0.572	1.00	0.88
KIAA1456	8	12,811,580	rs2946504	**T**	G	1.08	(1.05 - 1.12)	5.00E-07	0.9	0.99	0.94
ADH5P4	6	66,618,657	rs79976124	**A**	G	1.18	(1.1 - 1.26)	5.45E-07	4.03E-04	0.95	0.98
RPL19P16	10	122,849,667	rs35668226	**A**	G	1.1	(1.06 - 1.14)	9.86E-07	0.111	0.96	0.98
TOMM22P3	13	33,556,228	rs56252704	**A**	G	1.11	(1.07 - 1.16)	9.95E-07	0.264	0.98	0.96

Chr: chromosome
Risk: risk allele in the present Japanese study.
non-risk: alternative allele in the present Japanese study.
OR: odds ratio
95%CI: 95% confidence interval
P-for hetero: p-values of Cochran's Q-test for heterogeneity
R^2: r-square value for imputation accuracy

Supplementary Table 3. Association data of 17 suggestive loci for type 2 diabetes in Stage1+2 meta-analysis

			**Allele**			**Risk Allele**			**Association for type 2 diabetes**			**Meta analysis**	
nearby gene	**Chr.**	**SNP ID**	**Risk**	Non-risk	**Study**	**Case**	**Control**	**R^2**	**OR**	**95%CI**	***P* value**	**p-for associatio**	**p-for hetero**
CCDC85A	2	rs1116357	**G**	A	Stage-1, set-1	0.292	0.269	0.9999	1.12	(1.07 - 1.17)	6.55.E-06		
					Stage-1, set-2	0.299	0.279	0.9996	1.10	(1.05 - 1.15)	2.61.E-05		
					Stage-2	0.294	0.284		1.05	(0.99 - 1.11)	8.65.E-02		
					**combined**				**1.09**	**(1.06 - 1.12)**		**6.97.E-10**	**0.21**
FAM60A	12	rs147538848	**A**	G	Stage-1, set-1	0.205	0.19	0.867	1.11	(1.05 - 1.18)	5.81.E-04		
					Stage-1, set-2	0.199	0.184	0.912	1.12	(1.06 - 1.18)	1.15.E-04		
					Stage-2	0.21	0.193		1.11	(1.04 - 1.18)	1.10.E-03		
					**combined**				**1.11**	**(1.07 - 1.15)**		**7.83.E-10**	**0.99**
DMRTA1	9	rs1575972 *	**T**	A	Stage-1, set-1	0.947	0.935	0.979	1.24	(1.13 - 1.36)	8.64.E-06		
					Stage-1, set-2	0.943	0.935	0.883	1.17	(1.07 - 1.29)	1.13.E-03		
					Stage-2	0.95	0.942		1.16	(1.04 - 1.29)	7.60.E-03		
					**combined**				**1.19**	**(1.13 - 1.26)**		**1.50.E-09**	**0.62**
ASB3	2	rs9309245	**G**	C	Stage-1, set-1	0.178	0.164	0.997	1.10	(1.04 - 1.17)	1.57.E-03		
					Stage-1, set-2	0.186	0.168	0.998	1.14	(1.08 - 1.20)	3.43.E-06		
					Stage-2	0.181	0.172		1.06	(0.998 - 1.14)	5.95.E-02		
					**combined**				**1.10**	**(1.07 - 1.14)**		**1.25.E-08**	**0.30**
ATP8B2	1	rs67156297	**A**	G	Stage-1, set-1	0.102	0.086	0.966	1.21	(1.12 - 1.30)	1.28.E-06		
					Stage-1, set-2	0.097	0.087	0.955	1.13	(1.05 - 1.21)	1.34.E-03		
					Stage-2	0.092	0.087		1.07	(0.98 - 1.16)	1.33.E-01		
					**combined**				**1.14**	**(1.09 - 1.19)**		**1.95.E-08**	**0.10**
MIR4686	11	rs7107784 *	**G**	A	Stage-1, set-1	0.099	0.089	0.833	1.16	(1.07 - 1.26)	4.91.E-04		
					Stage-1, set-2	0.101	0.09	0.821	1.18	(1.09 - 1.27)	3.88.E-05		
					Stage-2	0.093	0.086		1.09	(0.998 - 1.19)	5.58.E-02		
					**combined**				**1.14**	**(1.09 - 1.20)**		**2.07.E-08**	**0.42**
INAFM2	15	rs67839313	**C**	T	Stage-1, set-1	0.28	0.261	0.999	1.10	(1.05 - 1.16)	7.87.E-05		
					Stage-1, set-2	0.278	0.264	0.883	1.08	(1.03 - 1.14)	1.75.E-03		
					Stage-2	0.281	0.267		1.07	(1.01 - 1.13)	1.36.E-02		
					**combined**				**1.09**	**(1.06 - 1.12)**		**2.42.E-08**	**0.73**
RPL19P16	10	rs35668226	**A**	G	Stage-1, set-1	0.805	0.795	0.962	1.07	(1.01 - 1.13)	2.55.E-02		
					Stage-1, set-2	0.812	0.792	0.977	1.13	(1.07 - 1.19)	4.15.E-06		
					Stage-2	0.814	0.805		1.06	(0.996 - 1.13)	6.43.E-02		
					**combined**				**1.09**	**(1.05 - 1.13)**		**2.67.E-07**	**0.18**
C16orf74	16	rs377457	**A**	G	Stage-1, set-1	0.708	0.687	0.987	1.10	(1.05 - 1.15)	7.48.E-05		
					Stage-1, set-2	0.711	0.697	0.894	1.08	(1.03 - 1.13)	1.93.E-03		
					Stage-2	0.705	0.697		1.04	(0.98 - 1.10)	1.80.E-01		
					**combined**				**1.08**	**(1.05 - 1.11)**		**6.11.E-07**	**0.25**
ADH5P4	6	rs79976124	**A**	G	Stage-1, set-1	0.068	0.053	0.953	1.34	(1.22 - 1.48)	1.53.E-09		
					Stage-1, set-2	0.066	0.063	0.976	1.06	(0.97 - 1.16)	1.70.E-01		
					Stage-2	0.066	0.062		1.05	(0.95 - 1.17)	3.17.E-01		
					**combined**				**1.14**	**(1.08 - 1.21)**		**1.78.E-06**	**3.68.E-04**
ZBTB20	3	rs73230612	**T**	C	Stage-1, set-1	0.644	0.631	0.99	1.06	(1.02 - 1.11)	9.63.E-03		
					Stage-1, set-2	0.658	0.634	0.999	1.11	(1.07 - 1.16)	1.86.E-06		
					Stage-2	0.642	0.642		1.003	(0.95 - 1.06)	9.01.E-01		
					**combined**				**1.07**	**(1.04 - 1.09)**		**3.25.E-06**	**0.01**
KIAA1456	8	rs2946504	**T**	G	Stage-1, set-1	0.413	0.393	0.986	1.09	(1.04 - 1.14)	3.68.E-04		
					Stage-1, set-2	0.418	0.401	0.938	1.08	(1.04 - 1.13)	5.54.E-04		
					Stage-2	0.397	0.393		1.02	(0.97 - 1.07)	5.39.E-01		
					**combined**				**1.06**	**(1.04 - 1.09)**		**3.97.E-06**	**0.11**
SALL4P5	3	rs2688419	**T**	C	Stage-1, set-1	0.667	0.647	0.914	1.10	(1.05 - 1.16)	5.43.E-05		
					Stage-1, set-2	0.671	0.652	0.923	1.09	(1.04 - 1.15)	1.32.E-04		
					Stage-2	0.662	0.662		1.0001	(0.95 - 1.05)	9.97.E-01		
					**combined**				**1.07**	**(1.04 - 1.10)**		**4.74.E-06**	**0.01**
OR2BH1P	11	rs2933170 *	**A**	G	Stage-1, set-1	0.677	0.662	0.9999	1.07	(1.02 - 1.12)	5.29.E-03		
					Stage-1, set-2	0.695	0.674	0.9998	1.11	(1.06 - 1.16)	1.47.E-05		
					Stage-2	0.683	0.682		1.01	(0.95 - 1.06)	8.35.E-01		
					**combined**				**1.06**	**(1.04 - 1.09)**		**9.83.E-06**	**0.031**
FDX1L	19	rs187060802 **	**A**	G	Stage-1, set-1	0.022	0.010	0.973	2.17	(1.79 - 2.64)	2.46.E-16		
					Stage-1, set-2	0.020	0.019	0.931	1.05	(0.90 - 1.23)	5.09.E-01		
					Stage-2	failed						N/A	N/A
MIR4704	13	rs4884660 **	**T**	C	Stage-1, set-1	0.225	0.206	0.991	1.12	(1.06 - 1.18)	4.43.E-05		
					Stage-1, set-2	0.227	0.214	0.991	1.08	(1.03 - 1.14)	2.15.E-03		
					Stage-2	failed						N/A	N/A
TOMM22P3	13	rs56252704 **	**A**	G	Stage-1, set-1	0.149	0.139	0.982	1.08	(1.02 - 1.15)	1.14.E-02		
					Stage-1, set-2	0.155	0.140	0.963	1.14	(1.07 - 1.21)	2.15.E-05		
					Stage-2	failed						N/A	N/A

Chr:chromosome

Risk: risk allele in the present Japanese study.

non-risk: alternative allele in the present Japanese study.

R^2 : r-square value for the imputation accuracy

OR: odds ratio

95%CI: 95% confidence interval

P-for hetero: p-values of Cochran's Q-test for heterogeneity

* proxy SNPs for the lead SNPs at Stage-1 GWAS meta-analysis

(rs1575972: proxy for rs12000501 *JPT* r^2 = 0.86, rs7107784: proxy for rs4930045 *JPT* r^2 = 0.86, rs2933170: proxy for rs1965305 *JPT* r^2 = 0.98)

** *de novo* typing was not successful

Supplementary Table 4. Association data of 7 novel type 2 diabetes susceptible loci with adjustment for age, sex and BMI

nearby gene	Chr.	SNP ID	Allele: Risk	Allele: Non-risk	Study	Risk Allele: Case	Risk Allele: Control	R^2	Association for type 2 diabetes: OR	95%CI	*P* value	Meta analysis: p-for association	Meta analysis: p-for hetero
CCDC85A	2	rs1116357	**G**	A	Stage-1, set-1	0.292	0.271	1	1.09	(1.03 - 1.15)	2.14.E-03		
					Stage-1, set-2	0.300	0.278	1	1.11	(1.06 - 1.16)	2.03.E-05		
					Stage-2	0.291	0.283		1.03	(0.97 - 1.09)	3.95.E-01		
					**combined**				**1.08**	**(1.05 - 1.12)**		**6.03.E-07**	**0.148**
FAM60A	12	rs147538848	**A**	G	Stage-1, set-1	0.205	0.192	0.867	1.11	(1.04 - 1.19)	1.89.E-03		
					Stage-1, set-2	0.199	0.184	0.912	1.13	(1.07 - 1.19)	4.66.E-05		
					Stage-2	0.209	0.193		1.16	(1.08 - 1.24)	3.88.E-05		
					**combined**				**1.13**	**(1.09 - 1.17)**		**6.73E-11**	**0.668**
DMRTA1	9	rs1575972	**T**	A	Stage-1, set-1	0.947	0.935	0.979	1.29	(1.16 - 1.43)	1.85.E-06		
					Stage-1, set-2	0.943	0.934	0.883	1.19	(1.08 - 1.31)	4.17.E-04		
					Stage-2	0.949	0.943		1.13	(0.995 - 1.28)	5.92.E-02		
					**combined**				**1.21**	**(1.14 - 1.29)**		**1.72E-09**	**0.237**
ASB3	2	rs9309245	**G**	C	Stage-1, set-1	0.178	0.166	0.997	1.09	(1.02 - 1.17)	8.73.E-03		
					Stage-1, set-2	0.186	0.168	0.998	1.14	(1.08 - 1.21)	7.08.E-06		
					Stage-2	0.181	0.173		1.06	(0.98 - 1.14)	1.22.E-01		
					**combined**				**1.10**	**(1.06 - 1.15)**		**2.10E-07**	**0.278**
ATP8B2	1	rs67156297	**A**	G	Stage-1, set-1	0.103	0.087	0.966	1.21	(1.11 - 1.32)	8.52.E-06		
					Stage-1, set-2	0.097	0.087	0.955	1.15	(1.06 - 1.24)	4.89.E-04		
					Stage-2	0.092	0.087		1.09	(0.99 - 1.20)	9.69.E-02		
					**combined**				**1.15**	**(1.10 - 1.21)**		**2.22E-08**	**0.239**
MIR4686	11	rs7107784	**G**	A	Stage-1, set-1	0.100	0.089	0.833	1.16	(1.05 - 1.27)	2.17.E-03		
					Stage-1, set-2	0.102	0.090	0.821	1.17	(1.08 - 1.27)	1.52.E-04		
					Stage-2	0.093	0.086		1.10	(0.99 - 1.21)	6.87.E-02		
					**combined**				**1.15**	**(1.09 - 1.21)**		**2.72E-07**	**0.590**
INAFM2	15	rs67839313	**C**	T	Stage-1, set-1	0.281	0.261	0.999	1.11	(1.05 - 1.17)	1.58.E-04		
					Stage-1, set-2	0.278	0.264	0.883	1.09	(1.03 - 1.14)	1.59.E-03		
					Stage-2	0.280	0.268		1.06	(0.998 - 1.13)	5.72.E-02		
					**combined**				**1.09**	**(1.05 - 1.12)**		**2.02E-07**	**0.570**

Chr:chromosome
Risk: risk allele in the present Japanese study.
non-risk: alternative allele in the present Japanese study.
R^2 : r-square value for the imputation accuracy
OR: odds ratio
95%CI: 95% confidence interval
P-for hetero: p-values of Cochran's Q-test for heterogeneity
Stage-1, set-1; Case n = 9,052, Control n = 5,677, whose clinical information for age, sex and BMI are complete
Stage-1, set-2; Case n = 5,582, Control n = 18,102, whose clinical information for age, sex and BMI are complete
Stage-2; Case n = 7,102, Control n = 5,253, whose clinical information for age, sex and BMI are complete
(total n = 50,768)

Supplementary Table 5 Conditional analysis for *DMRTA1* locus in the Japanese populations

SNP ID	Covariates	CHR	POS (Build37)	Risk	Non-risk		CASE RAF	CTRL RAF	R^2	OR	95%CI	P-value	P-het
rs1575972	-	9	22,301,092	**T**	A	Stage-1, set-1	0.947	0.935	0.979	1.24	(1.13 - 1.36)	8.64.E-06	
						Stage-1, set-2	0.943	0.935	0.883	1.17	(1.07 - 1.29)	1.13.E-03	
						Stage-2	0.950	0.942		1.16	(1.04 - 1.30)	7.37.E-03	
						**combined**				**1.19**	(**1.13 - 1.26**)	**1.43.E-09**	0.64
rs1575972		9	22,301,092	**T**	A	Stage-1, set-1	0.947	0.935	0.979	1.23	(1.12 - 1.35)	1.40.E-05	
	rs10811661	9	22,134,094	**T**	C	Stage-1, set-2	0.943	0.935	0.883	1.17	(1.07 - 1.29)	8.19.E-04	
						Stage-2	0.950	0.942		1.16	(1.04 - 1.30)	8.52.E-03	
						**combined**				**1.19**	(**1.12 - 1.26**)	**2.45.E-09**	0.68

RAF; risk allele freqiency , R^2; r-square value for imputaion accuracy, OR; odds ratio , 95%CI; 95% confidence interval

P-het; P-value of Cochran's Q-test for heterogeneity

Stage-1, set-1; 9,817 cases and 6,763 controls, imputed

Stage-1, set-2 ; 5,646 cases and 19,420 controls, imputed

Stage-2; 7,637 cases and 5,093 controls with complete *denovo* genotyped data for rs1575972 and rs10811661

Supplementary Table 6. Conditional analysis for *MIR4686* locus in the Japanese populations

SNP ID	Covariates	CHR	POS (Build37)	Risk	Non-risk		CASE RAF	CTRL RAF	R^2	OR	95%CI	P-value	P-het
rs7107784	-	11	2,215,089	**G**	A	Stage-1, set-1	0.099	0.089	0.833	1.16	(1.07 - 1.26)	4.91.E-04	
						Stage-1, set-2	0.101	0.090	0.821	1.18	(1.09 - 1.27)	3.88.E-05	
						Stage-2	0.095	0.084		1.14	(1.01 - 1.28)	3.11.E-02	
						**combined**				**1.16**	(**1.11** - **1.22**)	**5.77.E-09**	0.89
rs7107784		11	2,215,089	**G**	A	Stage-1, set-1	0.099	0.089	0.833	1.15	(1.06 - 1.25)	1.04.E-03	
	rs2237897	11	2,837,316	**C**	T	Stage-1, set-2	0.101	0.090	0.821	1.17	(1.08 - 1.26)	6.97.E-05	
						Stage-2	0.095	0.084		1.13	(1.01 - 1.27)	3.86.E-02	
						**combined**				**1.16**	(**1.10** - **1.22**)	**2.75.E-08**	0.88

RAF; risk allele freqiency , R^2; r-square value for imputaion accuracy, OR; odds ratio , 95%CI; 95% confidence interval
P-het; P-value of Cochran's Q-test for heterogeneity
Stage-1, set-1; 9,817 cases and 6,763 controls, imputed
Stage-1, set-2; 5,646 cases and 19,420 controls, imputed
Stage-2; 6,692cases and 2,516 controls with complete *denovo* genotyped data for rs7107784 and rs2237897

Supplementary Table 7. Association of 7 novel T2D loci with quantitative traits in non-diabetic controls

Locus	Chr	SNPID	Risk	FPG[a]			HOMA-β[a]			HOMA-IR[a]			covariates
				β	s.e.	p-value	β	s.e.	p-value	β	s.e.	p-value	
CCDC85A	2	rs1116357	**G**	0.031	0.020	0.113	0.032	0.025	0.197	0.041	0.025	0.093	-
				0.029	0.019	0.123	0.024	0.022	0.277	0.034	0.021	0.100	age, sex and logeBMI
FAM60A	12	rs147538848	**A**	0.016	0.022	0.459	0.019	0.029	0.518	0.018	0.028	0.522	-
				0.014	0.021	0.509	0.023	0.026	0.373	0.023	0.024	0.341	age, sex and logeBMI
DMRTA1	9	rs1575972	**T**	0.021	0.036	0.567	-0.023	0.045	0.616	0.020	0.045	0.650	-
				0.014	0.035	0.687	-0.012	0.041	0.770	0.027	0.038	0.483	age, sex and logeBMI
ASB3	2	rs9309245	**G**	-0.025	0.022	0.256	0.075	0.029	0.009	0.050	0.029	0.078	-
				-0.030	0.021	0.163	0.065	0.026	0.012	0.038	0.024	0.116	age, sex and logeBMI
ATP8B2	1	rs67156297	**A**	-0.004	0.031	0.910	-0.025	0.041	0.540	-0.027	0.041	0.507	-
				0.007	0.030	0.821	0.001	0.037	0.972	0.014	0.034	0.679	age, sex and logeBMI
MIR4686	11	rs7107784	**G**	0.083	0.032	0.009	0.024	0.041	0.555	0.087	0.040	0.031	-
				0.065	0.030	0.032	0.030	0.036	0.409	0.082	0.034	0.015	age, sex and logeBMI
INAFM2	15	rs67839313	**C**	0.018	0.019	0.360	-0.008	0.025	0.756	-0.002	0.025	0.925	-
				0.019	0.018	0.312	-0.014	0.023	0.548	-0.006	0.021	0.766	age, sex and logeBMI

FPG: Fasting Plasma Glucose, HOMA-β:homeostatic model assessment for beta cell function, HOMA-IR: HOMA for insulin resistance

Risk: risk allele for type 2 diabetes in the present study

a values are log-transformed for the analyses

β: β-coefficient , s.e.: standard error, p-value: p-value for the association

n = 1,744 for FPG, n = 1,324 for HOMA-β, n = 1,324 for HOMA-IR,

Supplementary Table 8. Association look-up data for novel 7 T2D loci in a meta analysis of quantitative glycaemic traits in non-diabetic individuals of Europeam decent from MAGIC Investigators

1) Fasting Plasma Glucose(FPG) and 2h-glucose in up to 133,010 and 42,854 non-diabetic individuals from Metabochip replication data sets*

						FPG			2h glucose		
SNPID	Risk	Other	Proxy SNP	Risk	Other	β	s.e.	P-value	β	s.e.	P-value
rs1116357	**G**	A	N/A								
rs147538848	**A**	G	N/A								
rs1575972	**T**	A	rs11791293	**C**	T	1.30.E-02	6.50.E-03	0.052	-4.20.E-03	3.60.E-02	0.91
rs9309245	**G**	C	N/A								
rs67156297	**A**	G	N/A								
rs7107784	**G**	A	rs7111341	**T**	C	2.20.E-03	2.50.E-03	0.373	4.00.E-04	1.40.E-02	0.98
rs67839313	**C**	T	N/A								

2) Fasting Plasma Insulin (FPI) and FPI adjusted for BMI in up to 108,557 individuals from Metabochip replication data sets*

						FPI			FPI adjusted for BMI		
SNPID	Risk	Other	Proxy SNP	Risk	Other	β	s.e.	P-value	β	s.e.	P-value
rs1116357	**G**	A	N/A								
rs147538848	**A**	G	N/A								
rs1575972	**T**	A	rs11791293	**C**	T	-2.20.E-02	7.70.E-03	**0.0039**	-2.00.E-02	6.80.E-03	**0.0027**
rs9309245	**G**	C	N/A								
rs67156297	**A**	G	N/A								
rs7107784	**G**	A	rs7111341	**T**	C	7.80.E-03	2.90.E-03	**0.0066**	4.10.E-03	2.40.E-03	0.0888
rs67839313	**C**	T	N/A								

3) Fasting Plasma Glucose(FPG) and FPG adjusted for BMI in up to 58,074 individuals **

						FPG			FPG adjusted for BMI		
SNPID	Risk	Other	Proxy SNP	Risk	Other	β	s.e.	P-value	β	s.e.	P-value
rs1116357	**G**	A				1.80.E-03	3.10.E-03	0.56	1.00.E-03	3.20.E-03	0.74
rs147538848	**A**	G	N/A								
rs1575972	**T**	A				1.20.E-02	9.90.E-03	0.21	1.10.E-02	1.00.E-02	0.28
rs9309245	**G**	C				-2.40.E-03	3.20.E-03	0.45	5.00.E-04	3.30.E-03	0.89
rs67156297	**A**	G	rs12025518	**c**	a	-8.00.E-04	3.70.E-03	0.84	-2.40.E-03	3.80.E-03	0.53
rs7107784	**G**	A	rs7111341	**t**	c	-2.00.E-03	3.90.E-03	0.61	-4.90.E-03	3.90.E-03	0.21
rs67839313	**C**	T	rs4924456	**g**	c	3.00.E-04	3.80.E-03	0.95	8.00.E-04	3.90.E-03	0.84

4) Fasting Plasma Insulin (FPI) and FPI adjusted for BMI in up to 51,750 individuals**

						FPI			FPI adjusted for BMI		
SNPID	Risk	Other	Proxy SNP	Risk	Other	β	s.e.	P-value	β	s.e.	P-value
rs1116357	**G**	A				5.40.E-03	3.10.E-03	0.084	6.00.E-03	2.70.E-03	**0.02436**
rs147538848	**A**	G	N/A								
rs1575972	**T**	A				-2.50.E-02	1.10.E-02	**0.019**	-2.60.E-02	9.30.E-03	**0.004821**
rs9309245	**G**	C				-3.00.E-04	3.30.E-03	0.94	-2.90.E-03	2.80.E-03	0.2943
rs67156297	**A**	G	rs12025518	**c**	a	3.90.E-03	3.70.E-03	0.30	3.40.E-03	3.20.E-03	0.2869
rs7107784	**G**	A	rs7111341	**t**	c	5.40.E-03	3.90.E-03	0.17	1.20.E-03	3.30.E-03	0.7157
rs67839313	**C**	T	rs4924456	**g**	c	4.00.E-04	3.80.E-03	0.92	1.00.E-04	3.30.E-03	0.9739

5) HbA1c association results in up to 46,368 individuals***

						HbA1c		
SNPID	Risk	Other	Proxy SNP	Risk	Other	β	s.e.	P-value
rs1116357	**G**	A				3.00.E-04	3.40.E-03	0.93
rs147538848	**A**	G	N/A					
rs1575972	**T**	A				1.35.E-02	1.04.E-02	0.19
rs9309245	**G**	C				8.10.E-03	3.50.E-03	**0.022**
rs67156297	**A**	G	rs12025518	**c**	a	4.10.E-03	4.00.E-03	0.31
rs7107784	**G**	A	rs7111341	**t**	c	2.00.E-03	4.70.E-03	0.67
rs67839313	**C**	T	rs4924456	**g**	c	-1.60.E-03	4.80.E-03	0.73

FPG; Fasting plasma glucose, FPI; Fasting plasma insulin
β;beta-coefficient, s.e.; standard error
Data on glycaemic traits have been contributed by MAGIC investigators and have been downloaded from www.magicinvestigators.org
* Scott RA et al. Nature genetics 2012;44;9;991-1005
**Manning AK et al. Nature genetics 2012;44;6;659-69
***Soranzo N et al.Diabetes 2010;59;12;3229-39
Best proxy SNPs used in the analysis
rs1575972-T ; rs11791293-C (*CEU* r^2 = 1)
rs67156297-A; rs12025518-C (*CEU* r^2 = 1)
rs7107784-G; rs7111341-T (*CEU* r^2 = 0.84)
rs67839313-C; rs4924456-G (*JPT* r^2 = 0.84)

Supplementary Table 9.Information on each analysis and Sample

Sample	Ethnicity	Sample size			Genotyping				SNP imputation				Association test
		Case	Control	Total	Platform	QC (SNP)	QC (Sample)	covariate	Method	Reference panel	QC1	QC2	
Stage-1, set-1 (BBJ)	Japanese	9,817	6,763	16,580	Omni express exome	Call rate > 0.99	Call rate > 0.98	none	MACH and Minimac	1000 Genomes Phase 1 (March 2012) (CHB+CHS+JPT)	$r^2 \geq 0.7$	$r^2 \geq 0.7$	mach2dat
Stage-1, set-2 (BBJ)	Japanese	5,646	19,420	25,066	Illumina humanhap 610Quad	Call rate > 0.99	Call rate > 0.98	none	MACH and Minimac	1000 Genomes Phase 1 (March 2012) (CHB+CHS+JPT)	$r^2 \geq 0.7$	$r^2 \geq 0.7$	mach2dat
Stage-2	Japanese	7,936	5,539	13,475	Multiplex PCR invader assay	Call rate > 0.95	NA	none	NA	NA	NA	NA	SAS-JMP
SDIID/SDS	Chinese	3,341	3,353	6,694	MassARRAY	Call rate > 0.95	NA	none	NA	NA	NA	NA	SAS
HK1 (HK GWAS)	Chinese	99	99	198	Illumina Hap550	Call rate > 0.99	Call rate > 0.95	none	MACH 1.0	1000 G(March 2010 release) 60 CHB+JPT	$r^2 \geq 0.5$	$r^2 \geq 0.7$	mach2dat
HK2 (HN GWAS)	Chinese	388	659	1,047	Illumina humanhap 610Quad	Call rate > 0.99	Call rate > 0.95	none	MACH 1.0	1000 G(March 2010 release) 60 CHB+JPT	$r^2 \geq 0.5$	$r^2 \geq 0.7$	mach2dat
SDGS	Chinese	1,425	3,764	5,189	Affymetrix 6.0	Call rate > 0.99	Call rate > 0.95	age, study	Minimac	1000G Phase I, 1092 samples	$r^2 \geq 0.3$	$r^2 \geq 0.7$	mach2dat
SCES	Chinese	302	1,090	1,392	Illumina humanhap 610Quad	Call rate > 0.99	Call rate > 0.95	none	IMPUTE ver 2	1000G Phase I v3 (March 2012), 1092 samples	info ≥ 0.5	info ≥ 0.7	SNPTEST
SIMES	Malay	794	1,240	2,034	Illumina humanhap 610Quad	Call rate > 0.99	Call rate > 0.95	none	IMPUTE ver 2	1000G Phase I v3 (March 2012), 1092 samples	info ≥ 0.5	info ≥ 0.7	SNPTEST
sp1M	Chinese	928	939	1,867	Illumina humanhap 1Mduov3	Call rate > 0.99	Call rate > 0.95	none	IMPUTE ver 2	1000G Phase I v3 (March 2012), 1092 samples	info ≥ 0.5	info ≥ 0.7	SNPTEST
sp610	Chinese	1,082	1,006	2,088	Illumina humanhap 610Quad	Call rate > 0.99	Call rate > 0.95	none	IMPUTE ver 2	1000G Phase I v3 (March 2012), 1092 samples	info ≥ 0.5	info ≥ 0.7	SNPTEST
KARE	Korean	1,042	2,943	3,985	Affymetrix 5.0	Call rate > 0.99	Call rate > 0.95	age, sex ,area	Minimac	1000G Phase I v3 Shapeit2, 1092 samples	-	$r^2 \geq 0.7$	mach2dat
H2T2DS	Korean	1,183	1,305	2,488	Affymetrix 6.0	Call rate > 0.99	Call rate > 0.95	age, sex	Minimac	1000G Phase I v3 Shapeit2, 1092 samples	-	$r^2 \geq 0.7$	mach2dat
SNUH	Korean	1,970	985	2,955	Affymetrix Axiom® Biobank Plus	Call rate > 0.99	Call rate > 0.95	none	NA	NA	NA	NA	PLINK
Danish studies 1*	Danish	1,859*	6,922*	8,781*	Metabochip	Call rate > 0.95	Call rate > 0.95	age, sex ,BMI	NA	NA	NA	NA	PLINK
Danish studies 2**	Danish	4,107**	5,627**	9,734**	LGC Genomics	Call rate > 0.95	Call rate > 0.95	age, sex ,BMI	NA	NA	NA	NA	R 3.1.1
DIAGRAM3	European	12,171	56,862	69,033	***	***	***	***	***	***	***	NA	***
DIAGRAM Metabochip	European #	22,669	58,119	80,788	***	***	***	***	***	***	***	NA	***
SINDI	Indian	977	1,169	2,146	Illumina humanhap 610Quad	Call rate > 0.99	Call rate > 0.95	none	IMPUTE ver 2	1000G Phase I v3 (March 2012), 1092 samples	info ≥ 0.5	info ≥ 0.7	SNPTEST
PROMIS1	Indian	2,718	6,326	9,044	Illumina Omniexpress	Call rate > 0.99	Call rate > 0.95	age,sex, PCs1-5	IMPUTE ver 2	1000 G(March 2010 release)	info ≥ 0.9	info ≥ 0.9	SNPTEST
PROMIS2	Indian	4,365	3,984	8,349	Illumina 660 Quad	Call rate > 0.99	Call rate > 0.95	age,sex, PCs1-5	IMPUTE ver 2	1000 G(March 2010 release)	info ≥ 0.9	info ≥ 0.9	SNPTEST
RACE1	Indian	602	598	1,200	Illumina 660 Quad	Call rate > 0.99	Call rate > 0.95	age,sex, PCs1-5	IMPUTE ver 2	1000 G(March 2010 release)	info ≥ 0.9	info ≥ 0.9	SNPTEST
RACE2	Indian	1,084	1,527	2,611	IlluminaOmniexpress	Call rate > 0.99	Call rate > 0.95	age,sex, PCs1-5	IMPUTE ver 2	1000 G(March 2010 release)	info ≥ 0.9	info ≥ 0.9	SNPTEST
AIDHS/SDS	Punjabi Sikh	841	774	1,615	Illumina 660W Quad Beadchip	Call rate > 0.95	Call rate > 0.95	age, sex, BMI, 5 PC	IMPUTE ver 2	1000G Phase 1, V3 Shapeit2, 1092 samples	info > 0.8	info > 0.8	SNPTEST V 2.3.0
SIGMA	Mexican	3,848	4,366	8,214	Ilumina OMNI 2.5 array	Call rate > 0.98	Call rate > 0.98	age and BMI as liability covariates; sex, PC1 adn Pc2, as fixed covariates	IMPUTE v2.2	1000 Genomes Phase I integrated variant set (build 37 and haplotype release date in August, 2012)	info ≥ 0.6	info ≥ 0.7	LTSOFT v1 and Plink v1.08p

* Information on analyses for rs1575972 and rs7107784 in Inter99, Health2006 and SDC
** Information on analyses for rs1116357, rs67156297, rs67839313, rs9309245 and rs147538848 in Inter99, Vejle Biobank, ADDITION and SDC
*** Please refer the original article (Morris, A.P. et al. Nat. Genet. 44, 981–990 , 2012)
QC1: Information on quality control criteria for imputation accuracy in each cohort is shown
QC2: Association data with lower imputation quality, r2 ＜0.7 or info ＜0.7, were not used for the further meta-analyses.
\# primarily from European descent, but including 1,178 T2D and 2,472 controls of Pakistani descent

Supplementary Table 10. Association of novel 7 SNP loci with type 2 diabetes risk in the population other than Japanese

	SNP	OR	(95%CI)	Direction	*p-value*	P het
**East Asian**	**SNP**	**OR**	**(95%CI)**	**Direction**	***p-value***	**P het**
	rs1116357	1.03	(0.99 - 1.07)	+−+++−−++−+	2.06.E-01	9.81.E-01
	rs147538848	1.13	(1.05 - 1.20)	???+++++++?	**3.58.E-04**	8.82.E-01
	rs1575972	1.14	(1.02 - 1.26)	+−?++−+−+++	1.62.E-02	2.77.E-02
	rs9309245	1.04	(0.99 - 1.09)	?−−+−+++++−	1.64.E-01	4.01.E-01
	rs67156297	1.05	(0.99 - 1.12)	+−−++++−−+?	1.22.E-01	2.60.E-01
	rs7107784	1.001	(0.91 - 1.10)	?−−?−−+−??−	9.83.E-01	9.74.E-03
	rs67839313	1.09	(1.04 - 1.15)	+??++−++−++	**1.93.E-04**	5.00.E-01
**European**	**SNP**	**OR**	**(95%CI)**	**Direction**	***p-value***	**P het**
	rs1116357	1.01	(0.98 - 1.04)	++?	5.45.E-01	9.84.E-01
	rs147538848	N/A		N/A	N/A	N/A
	rs1575972	1.14	(1.07 - 1.21)	+++	**5.58.E-05**	5.61.E-01
	rs9309245	1.01	(0.98 - 1.04)	++?	4.74.E-01	7.99.E-01
	rs67156297	1.04	(0.999 - 1.08)	++?	5.90.E-02	8.80.E-01
	rs7107784	1.04	(1.02 - 1.07)	+++	**4.39.E-04**	4.31.E-01
	rs67839313	1.02	(0.98 - 1.06)	++?	3.61.E-01	1.78.E-01
**South Asian**	**SNP**	**OR**	**(95%CI)**	**Direction**	***p-value***	**P het**
	rs1116357	1.02	(0.98 - 1.05)	+−++−+	4.18.E-01	3.45.E-01
	rs147538848	1.07	(0.97 - 1.17)	−++??+	1.59.E-01	3.45.E-01
	rs1575972	1.13	(1.03 - 1.24)	++++++	1.21.E-02	4.47.E-01
	rs9309245	0.996	(0.96 - 1.04)	+−+−−+	8.21.E-01	6.27.E-01
	rs67156297	0.98	(0.94 - 1.03)	−−−??−	4.79.E-01	9.94.E-01
	rs7107784	1.09	(1.03 - 1.15)	+++??−	**1.60.E-03**	2.04.E-01
	rs67839313	1.03	(0.99 - 1.08)	−++??+	1.68.E-01	7.65.E-01
**Mexican/Latino**	**SNP**	**OR**	**(95%CI)**	**Direction**	***p-value***	**P het**
	rs1116357	0.99	(0.93 - 1.05)	−	5.96.E-01	N/A
	rs147538848	1.01	(0.74 - 1.37)	+	8.98.E-01	N/A
	rs1575972	1.004	(0.83 - 1.21)	+	9.92.E-01	N/A
	rs9309245	0.96	(0.90 - 1.03)	−	2.31.E-01	N/A
	rs67156297	1.08	(0.995 - 1.18)	+	8.19.E-02	N/A
	rs7107784	1.15	(1.05 - 1.25)	+	**6.00.E-04**	N/A
	rs67839313	1.06	(0.97 - 1.14)	+	1.16.E-01	N/A
**All populations**	**SNP**	**OR**	**(95%CI)**	**Direction**	***p-value***	**P het**
	rs1116357	1.01	(0.99 - 1.03)	+−+++−−++−+++?+−++−+−	1.99.E-01	9.58.E-01
	rs147538848	1.10	(1.05 - 1.16)	???+++++++????−++??++	**2.25.E-04**	8.15.E-01
	rs1575972	1.13	(1.08 - 1.18)	+−?++−+−+++++++++++++	**2.26.E-07**	1.26.E-01
	rs9309245	1.01	(0.99 - 1.03)	?−−+−+++++−++?+−+−−+−	5.50.E-01	5.75.E-01
	rs67156297	1.03	(1.001 - 1.05)	+−−++++−−+?++?−−−??−+	4.08.E-02	3.83.E-01
	rs7107784	1.05	(1.03 - 1.08)	?−−?−−+−??−++++++??−+	**7.05.E-07**	7.92.E-03
	rs67839313	1.05	(1.02 - 1.07)	+??++−++−++++?−++??++	**2.91.E-04**	4.11.E-01
**All populations including Japanese**	**SNP**	**OR**	**(95%CI)**	**Direction**	***p-value***	**P het**
	rs1116357	1.04	(1.02 - 1.06)	++++−+++−−++−+++?+−++−+−	5.03.E-06	8.13.E-02
	rs147538848	1.11	(1.08 - 1.14)	+++???+++++++????−++??++	**7.65.E-13**	9.40.E-01
	rs1575972	1.15	(1.11 - 1.19)	++++−?++−+−+++++++++++++	**5.55.E-15**	1.35.E-01
	rs9309245	1.03	(1.01 - 1.05)	+++?−−+−+++++−++?+−+−−+−	4.42.E-04	9.64.E-03
	rs67156297	1.05	(1.03 - 1.08)	++++−−++++−−+?++?−−−??−+	4.53.E-06	9.74.E-03
	rs7107784	1.07	(1.05 - 1.09)	+++?−−?−−+−??−++++++??−+	**1.16.E-11**	**7.63.E-04**
	rs67839313	1.06	(1.04 - 1.08)	++++??++−++−++++?−++??++	**2.14.E-10**	3.26.E-01
**Meta-analysis for 4 ethnic groups**	**SNP**	**OR**	**(95%CI)**	**Direction**	***p-value***	**P het**
	rs1116357	1.01	(0.99 - 1.03)	+++−	1.99.E-01	7.60.E-01
**1.East Asian other than Japanese**	rs147538848	1.10	(1.05 - 1.16)	+?++	**2.29.E-04**	5.75.E-01
**2.European**	rs1575972	1.13	(1.08 - 1.18)	++++	**2.24.E-07**	6.75.E-01
**3.South Asian**	rs9309245	1.01	(0.99 - 1.03)	++−−	5.48.E-01	3.46.E-01
**4.Mexican/Latino**	rs67156297	1.03	(1.00 - 1.05)	++−+	4.07.E-02	1.27.E-01
	rs7107784	1.05	(1.03 - 1.08)	++++	**7.11.E-07**	8.10.E-02
	rs67839313	1.05	(1.02 - 1.07)	++++	**2.93.E-04**	1.53.E-01
**Meta-analysis for 4 ethnic groups**	**SNP**	**OR**	**(95%CI)**	**Direction**	***p-value***	**P het**
	rs1116357	1.04	(1.02 - 1.06)	+++−	5.54.E-06	**3.33.E-03**
**1.East Asian including Japanese**	rs147538848	1.11	(1.08 - 1.14)	+?++	**8.21.E-13**	5.94.E-01
**2.European**	rs1575972	1.15	(1.11 - 1.19)	++++	**5.47.E-15**	3.68.E-01
**3.South Asian**	rs9309245	1.03	(1.01 - 1.05)	++−−	4.25.E-04	**1.85.E-04**
**4.Mexican/Latino**	rs67156297	1.05	(1.03 - 1.08)	++−+	4.34.E-06	**7.92.E-04**
	rs7107784	1.07	(1.05 - 1.09)	++++	**1.11.E-11**	1.34.E-02
	rs67839313	1.06	(1.04 - 1.08)	++++	**1.82.E-10**	2.72.E-02

RAF; risk allele freqiency , R^2; r-square value for imputaion accuracy, OR; odds ratio , 95%CI; 95% confidence interval

P-het; P-value of Cochran's Q-test for heterogeneity

Best proxy SNPs used in the analysis

rs1575972-T ; rs7030811-T (HK1 and HK2 *CHB* $r^2 = 1$), rs11791293-C (UCPH, DIAGRAM3 and DIAGRAM Metabochip *CEU* $r^2 = 1$)

rs67156297-A; rs6687971-C (HK1 and HK2 *CHB* $r^2 = 1$), rs12025518-C (DIAGRAM3 *CEU* $r^2 = 1$)

rs7107784-G; rs7111341-T (HK1, HK2, UCPH, DIAGRAM3 and DIAGRAM Metabochip *CHB* $r^2 = 0.83$, *CEU* $r^2 = 0.84$)

rs67839313-C; rs11856877-G (HK1 and HK2 *CHB* $r^2 = 1$), rs4924456-G (DIAGRAM3 *JPT* $r^2 = 0.84$)

Supplementary Table 11. BMI-differentiated analysis for establised T2D loci; heterogeneity p-value < 0.05

						BMI < 25			BMI ≥ 25			BMI < 25, BMI ≥ 25 combined			
Nearby gene	**Chr**	**position (build 37)**	**SNP**	**risk allele**	**non-risk allele**	**OR**	**95%CI**	**P-value**	**OR**	**95%CI**	**P-value**	**OR**	**95%CI**	**P-value**	**P-hetero**
KCNQ1	11	2,858,546	rs2237897	**C**	T	1.43	(1.37 - 1.49)	1.28.E-63	1.22	(1.15 - 1.31)	9.94.E-10	1.37	(1.32 - 1.41)	2.07.E-68	**8.89.E-05**
KCNQ1	11	2,858,440	rs2237896	**G**	A	1.41	(1.36 - 1.47)	5.22.E-62	1.22	(1.14 - 1.30)	1.46.E-09	1.35	(1.31 - 1.40)	1.06.E-66	**1.07.E-04**
IGF2BP2	3	185,529,080	rs1470579	**C**	A	1.25	(1.20 - 1.30)	1.08.E-26	1.11	(1.04 - 1.18)	2.75.E-03	1.21	(1.17 - 1.25)	1.53.E-26	1.88.E-03
IGF2BP2	3	185,530,290	rs6769511	**C**	T	1.25	(1.20 - 1.30)	1.81.E-26	1.10	(1.03 - 1.18)	3.32.E-03	1.21	(1.17 - 1.25)	2.68.E-26	2.17.E-03
IGF2BP2	3	185,511,687	rs4402960	**T**	G	1.25	(1.20 - 1.30)	5.19.E-26	1.10	(1.03 - 1.18)	3.73.E-03	1.21	(1.16 - 1.25)	7.19.E-26	2.61.E-03
ADCY5	3	123,139,863	rs79223353	**G**	A	1.13	(1.08 - 1.18)	8.73.E-08	0.99	(0.93 - 1.07)	8.81.E-01	1.09	(1.05 - 1.13)	9.59.E-06	2.59.E-03
HMG20A	15	77,747,190	rs7178572	**A**	G	1.09	(1.05 - 1.13)	1.93.E-05	1.00	(0.94 - 1.06)	9.93.E-01	1.06	(1.03 - 1.10)	3.00.E-04	2.27.E-02
CDC123	10	12,307,894	rs11257655	**T**	C	1.16	(1.12 - 1.21)	1.60.E-14	1.07	(1.01 - 1.14)	3.42.E-02	1.14	(1.10 - 1.17)	2.23.E-14	2.35.E-02
CDC123	10	12,314,997	rs10906115	**A**	G	1.13	(1.09 - 1.18)	2.07.E-10	1.05	(0.99 - 1.12)	1.08.E-01	1.11	(1.07 - 1.15)	4.89.E-10	3.91.E-02
POU5F1-TCF19	6	31,127,037	rs115801775	**C**	T	1.18	(1.10 - 1.26)	1.26.E-06	1.02	(0.92 - 1.14)	6.51.E-01	1.13	(1.07 - 1.20)	1.41.E-05	2.80.E-02
POU5F1-TCF19	6	31,136,453	rs115164593	**G**	A	1.11	(1.06 - 1.17)	2.40.E-05	1.01	(0.94 - 1.09)	7.63.E-01	1.08	(1.04 - 1.13)	1.94.E-04	4.43.E-02
TCF7L2	10	114,758,349	rs7903146	**T**	C	1.58	(1.45 - 1.72)	2.54.E-24	1.30	(1.12 - 1.51)	7.22.E-04	1.50	(1.39 - 1.62)	8.64.E-26	2.94.E-02
TCF7L2	10	114,754,071	rs34872471	**C**	T	1.58	(1.45 - 1.73)	4.63.E-24	1.30	(1.12 - 1.51)	7.52.E-04	1.50	(1.39 - 1.62)	1.63.E-25	2.97.E-02
SLC16A11/13	17	6,945,483	rs75418188	**T**	C	1.29	(1.20 - 1.38)	7.45.E-13	1.12	(1.00 - 1.26)	5.21.E-02	1.24	(1.17 - 1.32)	8.57.E-13	4.42.E-02

Chr:chromosome
Risk allele: risk allele in the present Japanese study
non-risk allele: alternative allele in the present Japanese study
OR: odds ratio
95%CI: 95% confidence interval
P-value: p-value for association with type 2 diabetes
P-for hetero: p-values of Cochran's Q-test for heterogeneity
All association data are adjusted for age, sex and loge-transformed BMI
This analyis includes Stage-1(set-1 + set-2) participants whose clinical information for age, sex and BMI are complete (total n= 38,413)
BMI < 25 ; total Case n = 9,841, Control n = 18,818, Stage-1,set-1: Case n = 6,105, Control n = 4,476, Stage-1, set-2: Case n = 3,736, Control n = 14,342
BMI ≥ 25 ; total Case n = 4,793, Control n = 4,961, Stage-1,set-1: Case n = 2,947, Control n = 1,201, Stage-1, set-2: Case n = 1,846, Control n = 3,760

Supplementary Table 12. Sex-differentiated analysis for previously reported T2D loci

1) heterogeneity between male and female ;p < 0.05

								male			female			male, female combined			
Nearby Gene	Chr	position	SNP	risk allele	non-risk allele	R^2 set-1	R^2 set-2	OR	95%CI	P-value	OR	95%CI	P-value	OR	95%CI	P-value	P-hetero
GCKR	2	27,741,237	rs780094	**C**	T	1.00	1.00	1.12	(1.07 - 1.17)	9.70.E-08	1.02	(0.97 - 1.08)	4.18.E-01	1.08	(1.05 - 1.12)	1.99.E-06	1.09.E-02
ANK1	8	41,509,259	rs12549902	**A**	G	1.00	1.00	1.15	(1.11 - 1.20)	9.01.E-12	1.08	(1.02 - 1.14)	8.32.E-03	1.13	(1.09 - 1.16)	2.03.E-12	4.45.E-02
HNF4A	20	42,989,267	rs4812829	**A**	G	0.99	1.00	1.05	(1.01 - 1.09)	1.96.E-02	1.13	(1.07 - 1.19)	1.35.E-05	1.08	(1.04 - 1.11)	7.62.E-06	3.68.E-02
SLC30A8	8	118,185,025	rs3802177	**G**	A	1.00	1.00	1.08	(1.04 - 1.13)	2.45.E-04	1.20	(1.14 - 1.27)	1.21.E-10	1.12	(1.09 - 1.16)	1.27.E-11	2.65.E-03
UBE2E2	3	23,454,565	rs1845900	**A**	G	0.95	0.95	1.14	(1.08 - 1.21)	7.22.E-06	1.28	(1.19 - 1.39)	4.96.E-10	1.19	(1.13 - 1.24)	3.01.E-13	1.77.E-02

2) Known T2D loci whichheterogeneity between male and female have been reported*

								male			female			male, female combined			
Nearby Gene	Chr	position	SNP	risk allele	non-risk allele	R^2 set-1	R^2 set-2	OR	95%CI	P-value	OR	95%CI	P-value	OR	95%CI	P-value	P-hetero
CCND2	12	4,374,373	rs11063069	**G**	A	0.99	0.33	1.17	(0.98 - 1.39)	8.52.E-02	0.93	(0.74 - 1.16)	5.05.E-01	1.07	(0.93 - 1.23)	3.43.E-01	1.13.E-01
CCND2	12	4,363,420	rs4766228	**A**	G	0.97	0.98	1.07	(1.03 - 1.12)	1.61.E-03	1.01	(0.95 - 1.06)	8.18.E-01	1.05	(1.01 - 1.08)	7.97.E-03	8.52.E-02
GIPR	19	46,158,513	rs8108269	**G**	T	1.00	1.00	1.07	(1.03 - 1.12)	1.34.E-03	1.13	(1.07 - 1.20)	2.55.E-05	1.09	(1.06 - 1.13)	3.67.E-07	1.43.E-01
KCNQ1	11	2,847,069	rs163184	**G**	T	1.00	1.00	1.23	(1.18 - 1.29)	1.37.E-23	1.24	(1.18 - 1.32)	2.10.E-14	1.24	(1.20 - 1.28)	2.29.E-36	8.32.E-01
KCNQ1	11	2,858,546	rs2237897	**C**	T	0.96	0.94	1.37	(1.31 - 1.44)	3.76.E-45	1.34	(1.26 - 1.42)	1.25.E-22	1.36	(1.31 - 1.41)	5.84.E-66	5.30.E-01
DGKB	7	14,898,282	rs17168486	**T**	C	0.99	0.99	1.05	(1.01 - 1.10)	1.45.E-02	1.07	(1.01 - 1.13)	1.81.E-02	1.06	(1.02 - 1.10)	7.40.E-04	6.67.E-01
GRB14	2	165,501,849	rs3923113	**A**	C	0.67	0.99	1.08	(1.00 - 1.16)	3.74.E-02	1.18	(1.06 - 1.31)	1.96.E-03	1.11	(1.05 - 1.18)	5.03.E-04	1.77.E-01
GRB14	2	165,528,876	rs13389219	**C**	T	1.00	0.99	1.11	(1.03 - 1.20)	4.88.E-03	1.19	(1.07 - 1.33)	1.13.E-03	1.14	(1.07 - 1.21)	2.98.E-05	2.97.E-01
BCL11A	2	60,568,745	rs243088	**T**	A	1.00	1.00	1.02	(0.98 - 1.07)	2.96.E-01	1.05	(0.99 - 1.11)	1.31.E-01	1.03	(1.00 - 1.07)	8.16.E-02	5.62.E-01

Chr:chromosome

Risk allele: risk allele in the present Japanese study.

non-risk allele: alternative allele in the present Japanese study.

R^2: r-square value for imputation accuracy

OR: odds ratio

95%CI: 95% confidence interval

P-value: p-value for association with type 2 diabetes

P-for hetero: p-values of Cochran's Q-test for heterogeneity

All association data are adjusted for age, sex and loge-transformed BMI

This analysis includes participants whose clinical information for age, sex and BMI are complete (total n= 38,413)

male ; total Case n = 13,947, Control n = 15,634, Stage-1, set-1: Case n = 5,749, Control n = 3,302, Stage-1, set-2: Case n = 3,823, Control n = 9,164

female ; total Case n = 7,806, Control n = 14,315, Stage-1,set-1: Case n =3,303, Control n = 2,375, Stage-1, set-2: Case n = 1,759, Control n = 8,938

* Morris, A.P. et al. Nat. Genet. 44, 981–990 (2012).

Supplementary Table 13. BMI-differentiated analysis for novel 7 SNP loci

					BMI < 25 Stage1+2 meta-analysis			BMI ≥ 25 Stage1+2 meta-analysis			BMI < 25, BMI ≥25 combined			
Nearby Gene	Chr	SNP	risk allele	non-risk allele	OR	95%CI	P-value	OR	95%CI	P-value	OR	95%CI	P-value	P-hetero
CCDC85A	2	rs1116357	**G**	A	1.08	(1.04 - 1.12)	1.75.E-05	1.08	(1.02 - 1.14)	1.33.E-02	1.08	(1.05 - 1.12)	7.39.E-07	0.86
FAM60A	12	rs147538848	**A**	G	1.14	(1.09 - 1.19)	6.99.E-09	1.10	(1.03 - 1.18)	6.80.E-03	1.13	(1.09 - 1.17)	2.29.E-10	0.47
DMRTA1	9	rs1575972	**T**	A	1.26	(1.17 - 1.36)	1.48.E-09	1.11	(0.99 - 1.24)	7.47.E-02	1.21	(1.14 - 1.29)	1.55.E-09	0.07
ASB3	2	rs9309245	**G**	C	1.10	(1.05 - 1.15)	4.13.E-05	1.11	(1.04 - 1.19)	1.93.E-03	1.10	(1.06 - 1.14)	2.98.E-07	0.69
ATP8B2	1	rs67156297	**A**	G	1.15	(1.09 - 1.22)	1.94.E-06	1.15	(1.05 - 1.26)	3.81.E-03	1.15	(1.09 - 1.21)	2.46.E-08	0.96
MIR4686	11	rs7107784	**G**	A	1.16	(1.09 - 1.23)	2.38.E-06	1.12	(1.01 - 1.24)	2.70.E-02	1.15	(1.09 - 1.21)	2.29.E-07	0.56
INAFM2	15	rs67839313	**C**	T	1.11	(1.07 - 1.15)	8.48.E-08	1.04	(0.98 - 1.10)	2.30.E-01	1.09	(1.05 - 1.13)	2.02.E-07	0.07

Chr:chromosome

Risk allele: risk allele in the present Japanese study.

non-risk allele: alternative allele in the present Japanese study.

R^2 : r-square value for the imputation accuracy

OR: odds ratio

95%CI: 95% confidence interval

P-value: p-value for association with type 2 diabetes

P-for hetero: p-values of Cochran's Q-test for heterogeneity

All association data are adjusted for age, sex and loge-transformed BMI

This analysis includes participants whose clinical information for age, sex and BMI are complete (total n= 50,768)

BMI < 25 ; total Case n = 14,366, Control n = 22,992, Stage-1, set-1: Case n = 6,105, Control n = 4,476, Stage-1, set-2: Case n = 3,736, Control n = 14,342, Stage-2: Case n = 4,525, Control n = 4,174

BMI ≥ 25 ; total Case n = 7,370, Control n = 6,040, Stage-1, set-1: Case n = 2,947, Control n = 1,201, Stage-1, set-2: Case n = 1,846, Control n = 3,760, Stage-2: Case n = 2,577, Control n = 1,079

Supplementary Table 14. Sex-differentiated analysis for novel 7 SNP loci

					male Stage1+2 meta-analysis			female Stage1+2 meta-analysis			male, female combined			
Nearby Gene	Chr	SNP	risk allele	non-risk allele	OR	95%CI	P-value	OR	95%CI	P-value	OR	95%CI	P-value	P-hetero
CCDC85A	2	rs1116357	**G**	A	1.10	(1.06 - 1.14)	2.00.E-06	1.05	(0.997 - 1.10)	6.41.E-02	1.08	(1.05 - 1.12)	1.01.E-06	0.15
FAM60A	12	rs147538848	**A**	G	1.12	(1.07 - 1.18)	1.42.E-06	1.14	(1.08 - 1.21)	1.38.E-05	1.13	(1.09 - 1.17)	8.95.E-11	0.67
DMRTA1	9	rs1575972	**T**	A	1.19	(1.10 - 1.29)	1.19.E-05	1.21	(1.10 - 1.34)	1.88.E-04	1.20	(1.13 - 1.28)	8.49.E-09	0.84
ASB3	2	rs9309245	**G**	C	1.08	(1.03 - 1.13)	1.06.E-03	1.14	(1.07 - 1.21)	3.15.E-05	1.10	(1.06 - 1.14)	2.67.E-07	0.21
ATP8B2	1	rs67156297	**A**	G	1.19	(1.12 - 1.27)	3.98.E-08	1.08	(0.996 - 1.17)	6.13.E-02	1.15	(1.09 - 1.21)	4.13.E-08	0.06
MIR4686	11	rs7107784	**G**	A	1.11	(1.04 - 1.18)	2.29.E-03	1.21	(1.11 - 1.32)	9.65.E-06	1.14	(1.09 - 1.21)	3.21.E-07	0.10
INAFM2	15	rs67839313	**C**	T	1.10	(1.05 - 1.14)	8.41.E-06	1.07	(1.01 - 1.13)	1.43.E-02	1.09	(1.05 - 1.12)	5.33.E-07	0.42

Chr:chromosome
Risk allele: risk allele in the present Japanese study.
non-risk allele: alternative allele in the present Japanese study.
OR: odds ratio
95%CI: 95% confidence interval
P-value: p-value for association with type 2 diabetes
P-for hetero: p-values of Cochran's Q-test for heterogeneity
All association data are adjusted for age, sex and loge-transformed BMI
This analysis includes participants whose clinical information for age, sex and BMI are complete (total n= 50,768)
male ; total Case n = 13,937, Control n = 14,956 Stage-1, set-1: Case n = 5,749, Control n = 3,302, Stage-1, set-2: Case n = 3,823, Control n = 9,164, Stage-2 Case n = 4,365, Control n = 2,490
female ; total Case n = 7,799, Control n = 14,076 Stage-1, set-1: Case n = 3,303, Control n = 2,375, Stage-1, set-2: Case n = 1,759, Control n = 8,938, Stage-2 Case n = 2,737, Control n = 2,763

Supplementary Table15. Association of *ADCY5, PRC1* and *HNF1A* with type 2 diabetes in the Japanese populations

			Allele			Risk Allele Frequency			Association for type 2 diabetes			Meta analysis	
locus	Chr.	SNP	**Risk**	Non -risk	Study	Case	Control	R^2	OR	95%CI	p-value	p-for association	p-for hetero
PRC1	15	rs79548680	**C**	G	Stage-1, set-1	0.494	0.476	0.906	1.09	(1.04 - 1.14)	4.11.E-04		
					Stage-1, set-2	0.490	0.475	0.903	1.07	(1.02 - 1.11)	4.50.E-03		
					Stage-2	0.501	0.482		1.08	(1.03 - 1.13)	2.40.E-03		
					**combined**				**1.08**	**(1.05 - 1.11)**		**3.64E-08**	**0.83**
ADCY5	3	rs79223353	**G**	A	Stage-1, set-1	0.656	0.636	0.837	1.11	(1.05 - 1.16)	5.93.E-05		
					Stage-1, set-2	0.649	0.638	0.811	1.06	(1.01 - 1.11)	1.87.E-02		
					Stage-2	0.671	0.652		1.09	(1.03 - 1.15)	1.70.E-03		
					**combined**				**1.08**	**(1.05 - 1.12)**		**4.20E-08**	**0.47**
HNF1A	12	rs55783344	**T**	C	Stage-1, set-1	0.239	0.222	0.991	1.10	(1.04 - 1.16)	4.30.E-04		
					Stage-1, set-2	0.237	0.228	0.988	1.05	(1.00 - 1.10)	4.55.E-02		
					Stage-2	0.239	0.226		1.08	(1.02 - 1.14)	1.36.E-02		
					**combined**				**1.07**	**(1.04 - 1.11)**		**5.18E-06**	**0.49**

Chr:chromosome

Risk: risk allele in the present Japanese study.

non-risk: alternative allele in the present Japanese study.

R^2 : r-square value for the imputation accuracy

OR: odds ratio

95%CI: 95% confidence interval

P-for hetero: p-values of Cochran's Q-test for heteroegeneity

Supplementary Table 16. Conditional analysis for *IDE-HHEX* locus in the Japanese populations

SNP ID	Covariates	CHR	POS (Build37)	Risk	Non-risk		CASE RAF	CTRL RAF	R^2	OR	95%CI	P-value	P-het
rs78627331	-	10	94,812,254	**C**	A	Stage-1, set-1	0.042	0.030	0.89	1.43	(1.26 - 1.62)	2.10.E-08	
						Stage-1, set-2	0.039	0.032	0.84	1.25	(1.11 - 1.42)	3.08.E-04	
						Stage-2	0.043	0.032		1.33	(1.16 - 1.52)	3.19.E-05	
						**combined**				**1.33**	(**1.24 - 1.44**)	**1.65.E-14**	0.34
rs78627331		10	94,812,254	**C**	A	Stage-1, set-1	0.042	0.030	0.89	1.33	(1.16 - 1.51)	1.69.E-05	
	rs1111875	10	94,462,882	**C**	T	Stage-1, set-2	0.039	0.032	0.84	1.17	(1.03 - 1.32)	1.67.E-02	
						Stage-2	0.043	0.032		1.21	(1.07 - 1.38)	1.33.E-03	
						**combined**				**1.24**	(**1.15 - 1.34**)	**1.49.E-08**	0.37

SNP ID	Covariates	CHR	POS (Build37)	Risk	Non-risk		CASE RAF	CTRL RAF	R^2	OR	95%CI	P-value	P-het
rs34773007	-	10	94,658,207	**A**	G	Stage-1, set-1	0.052	0.039	0.99	1.36	(1.22 - 1.51)	1.59.E-08	
						Stage-1, set-2	0.046	0.040	0.98	1.18	(1.07 - 1.31)	1.50.E-03	
						Stage-2	0.049	0.037		1.35	(1.19 - 1.53)	3.89.E-06	
						**combined**				**1.28**	(**1.20 - 1.37**)	**1.72.E-14**	0.13
rs34773007		10	94,658,207	**A**	G	Stage-1, set-1	0.052	0.039	0.99	1.28	(1.14 - 1.43)	1.60.E-05	
	rs1111875	10	94,462,882	**C**	T	Stage-1, set-2	0.046	0.040	0.98	1.11	(1.00 - 1.23)	5.86.E-02	
						Stage-2	0.049	0.037		1.28	(1.12 - 1.46)	2.23.E-04	
						**combined**				**1.21**	(**1.13 - 1.29**)	**2.20.E-08**	0.13

RAF; risk allele freqiency , R^2; r-square value for imputaion accuracy, OR; odds ratio , 95%CI; 95% confidence interval

P-het; P-value of Cochran's Q-test for heterogeneity

Stage-1, set-1; 9,817 cases and 6,763 controls, imputed

Stage-1, set-2; 5,646 cases and 19,420 controls, imputed

Stage-2; 7,802 cases and 5,090 controls for rs78627331, 7,752 cases and 5,038 controls for rs34773007, *de novo* typed

Supplementary Table 17. Missense variant genes

T2D risk SNP		LD			
Lead SNP	population	r^2		*gene*	Missense variants
rs780094	European	0.906	*CEU*	*GCKR*	Leu446Pro
rs10203174	European	1	*CEU*	*THADA*	Thr1187Ala
rs1801282	European	1	*CEU*	*PPARG*	Pro12Ala
rs4458523	European	0.881	*CEU*	*WFS1*	Arg611His
rs35658696	European	1	*CEU*	*PAM*	Asp563Gly
		1	*CEU*	*PPIP5K2*	Ser1228Gly
rs115801775	Japanese	1	*JPT*	*CCHCR1*	Arg155Gln
		0.817	*JPT*	*PSORS1C1*	Pro133Leu
		0.95	*JPT*	*CDSN*	Ser453Asn
		0.95	*JPT*	*C6orf15*	Val5Met
rs3130501(rs115164593)	European	1	*CEU*	*TCF19*	Met211Val
rs1535500	East Asian	1	*JPT*	*KCNK16*	Ala277Glu, Pro254His
		1	*JPT*	*KCNK17*	Ser21Gly
rs13266634	Japanese	1	*JPT*	*SLC30A8*	Arg276Trp
rs28642252	Japanese	0.946	*JPT*	*GPSM1*	Ser391Leu
rs5215	European	1	*CEU*	*KCNJ11*	Val337Ile, Lys23Glu
		0.95	*CEU*	*ABCC8*	Ala1369Ser
rs391300	Chinese	1	*CHB*	*SMG6*	Ala972Thr
rs75418188	Japanese	1	*JPT*	*SLC16A11*	Gly340Ser, Pro443Thr, Val113Ile
rs10401969	European	1	*CEU*	*TM6SF2*	Glu167Lys

r^2; r-square value for Linkage Disequilibrium between T2D risk SNP and missense variants

Genes include missense variants, which were in linkage disequilibrium (r^2 ≥ 0.80) to T2D risk lead SNPs, were listed.

Supplementary Table 18. A list of monogenic diabetes genes

1) pancreatic β-cell function		2) Insulin resistance	
gene	**type**	***gene***	**type**
ABCC8	PNDM	*AGPAT2*	CGL
BLK	MODY11	*AKT2*	FPL, insulin signalling defects
CEL	MODY8	*ALMS1*	Astrom synd
CISD2	Wolfram syndrome (w/o insipidus)	*BLM*	Bloom synd
EIF2AK3	PNDM	*BSCL2*	CGL
GATA4	pancreatic agenesis	*CAV1*	CGL
GATA6	pancreatic agenesis	*CIDEC*	FPL
GCK	MODY2, PNDM	*INSR*	Type A insulin resistance
GLIS3	PNDM	***LEP***	SEO
HNF1A	MODY3, PNDM	*LMNA*	FPL
HNF1B	MODY5, PNDM	*MC4R*	SEO
HNF4A	MODY1, PNDM	*PCNT*	MOPDII
IER3IP1	PNDM	*PLIN1*	FPL
INS	MODY10, PNDM, TNDM	*POMC*	SEO
KCNJ11	PNDM	***PPARG***	FPL, insulin signalling defects
KLF11	MODY7, PNDM	*PPP1R3A*	insulin signalling defects
MNX1	PNDM	*PTRF*	CGL
NEUROD1	MODY6, PNDM	*SH2B1*	SEO
NGN3	PNDM	*TBC1D4*	insulin signalling defects
PAX4	MODY9, PNDM	*WRN*	Werner synd
PAX6	PNDM	*ZMPSTE24*	FPL
PDX1/IPF1	MODY4, PNDM	*DMPK*	Myotonic dystrophy
PTF1A	pancreatic agenesis		
RFX6	PNDM		
SLC19A2	PNDM		
SLC29A3	autoantibody negative insulin dependet diabetes		
SLC2A2	PNDM(fanconi Bickel sydrome), TNDM		
WFS1	Wolfram syndrome		
PLAGL1	TNDM		
ZFP57	TNDM		

PNDM；Permanent neonatal diabetes mellitus

TNDM；Transient neonatal diabetes mellitus

MODY; Maturity onset diabetes of the young

CGL；Generalized lipodystrophy

FPL ;Familial partial lipodystrophy

SEO;severe obesity

Eleven genes annotated as monogenic diabetes genes (see supplementary information) are indecated by bold letters

Supplementary Table 19. Overlap of T2D risk genes with mouse phenotypes

knockout mouse phenotype category	No. knockout mouse genes with human ortholog	No. overlap with T2D genes	P-value
**liver/biliary system phenotype**	**1004**	**21**	**0.00022**
**homeostasis/metabolism phenotype**	**3475**	**52**	**0.00052**
**endocrine/exocrine gland phenotype**	**1480**	**27**	**0.00055**
**embryogenesis phenotype**	**1518**	**27**	**0.00091**
**mortality/aging**	**4023**	**57**	**0.0013**
**growth/size/body phenotype**	**3163**	**47**	**0.0013**
renal/urinary system phenotype	1017	19	0.0030
cardiovascular system phenotype	2021	31	0.0069
digestive/alimentary phenotype	1155	19	0.017
adipose tissue phenotype	656	12	0.024
normal phenotype	1693	25	0.028
nervous system phenotype	2840	37	0.055
muscle phenotype	1234	18	0.075
integument phenotype	1522	21	0.10
taste/olfaction phenotype	125	3	0.11
behavior/neurological phenotype	2478	29	0.29
craniofacial phenotype	1010	13	0.30
vision/eye phenotype	1197	15	0.32
limbs/digits/tail phenotype	774	5	0.34
respiratory system phenotype	1158	14	0.41
pigmentation phenotype	362	5	0.43
tumorigenesis	805	10	0.44
cellular phenotype	2825	31	0.48
immune system phenotype	2667	28	0.68
other phenotype	274	3	0.84
hematopoietic system phenotype	2475	25	0.86
skeleton phenotype	1494	14	0.87
reproductive system phenotype	1581	15	0.90
hearing/vestibular/ear phenotype	529	5	0.94

Out of 286 T2D biological candidate genes, 169 mouse orthologs existed in the database. Overlap with 169 genes implicated in knockout mouse phenotypes (categorized in 29 phenotypes) was evaluated.Knockout mouse phenotypes that satisfied significant enrichment with T2D risk genes are indicated in bold (P for chi-square test < 0.05/29 = 0.0017)

Supplementary Table 20. Pubmed text-mining

GENE	GRAIL p-value
GCK	1.9.E-09
GCKR	4.4.E-08
HNF1A	8.2.E-08
PAX4	1.4.E-07
HNF1B	2.7.E-06
HNF4A	7.8.E-06
GIPR	1.6.E-05
NOTCH1	9.1.E-04
NOTCH2	1.3.E-03
TLE1	1.9.E-03
ABCC8	2.1.E-03
CCNE2	3.2.E-03
TCF7L2	5.1.E-03
FAH	7.6.E-03
WFS1	7.6.E-03
HHEX	1.2.E-02
KCNJ11	1.2.E-02
CDK2AP1	1.3.E-02
SBNO1	1.5.E-02
CCND2	1.5.E-02
POU5F1	1.6.E-02
GLIS3	2.2.E-02
ARL15	2.4.E-02
LEP	2.5.E-02
PPARG	3.2.E-02
SYN2	3.2.E-02
THADA	3.7.E-02
PAM	4.2.E-02
HKDC1	4.4.E-02
FBXW7	4.6.E-02

Keywords Describing Functional Connections

'diabetes‘, 'notch‘, 'glucose‘, 'cyclin‘, 'pancreatic‘, 'insulin‘, 'beta‘,
'hepatocyte‘, 'mutations‘, 'leptin‘, 'type‘, 'repressor‘, 'pancreas‘,
'laminin‘, 'mice‘, 'transcription‘, 'differentiation‘, 'development‘,

A list of genes prioritized by PubMed text mining using GRAIL with gene-based $p < 0.05$, to search for text similarity in PubMed abstracts (connectivity) across the 286 T2D risk genes

Supplementary Table 21. A list of direct interation between T2D biological genes and drug target genes

biological T2D genes	drug target genes which interact with T2D genes
ABCC8	*KCNJ8*
ABCC8	*KCNJ11*
GCK	*None*
KCNJ11	*KCNJ8*
KCNJ11	*ABCC8*
KIF11	*AURKA*
KIF11	*IKBKE*
PPARG	*HDAC3*
PPARG	*HDAC4*
PPARG	*MAPK1*
PPARG	*MAPK8*
PPARG	*RXRA*
PPARG	*RXRG*
NOTCH2	*GSK3B*
NOTCH1	*GSK3B*
CCND2	*GSK3B*
FBXW7	*JUN*
HHEX	*JUN*
CCND2	*JUN*
NOTCH2	*ADAM17*
NOTCH1	*ADAM17*
CCNE2	*CDK2*
CCND2	*CDK2*
TCF7L2	*CDK6*
CCND2	*CDK6*
SYN2	*HSPA8*
NOTCH1	*HSPA8*
LEP	*STAT3*
HNF1A	*STAT3*
TP53INP1	*TP53*
HNF4A	*TP53*
NOTCH1	*ABL2*
HNF1A	*ALB*
POU5F1	*AR*
HNF1A	*CACNA1A*
HNF1A	*CALM1*
HNF1A	*CALM2*
HNF1A	*CALM3*
CCND2	*CCND1*
CCND2	*CDK4*
LEP	*CLU*
LEP	*CNTFR*
LEP	*CRP*
NOTCH1	*CSF1*
LEP	*CSF2RA*
LEP	*CSF2RB*
LEP	*CSF3R*
LEP	*EPOR*
NOTCH1	*ERBB3*
HNF4A	*ESR1*
GCKR	*GCK*
LEP	*GHR*
HNF1A	*HDAC9*
NOTCH1	*HIF1A*

LEP	*IFNAR1*
LEP	*IFNAR2*
LEP	*IFNGR1*
LEP	*IFNGR2*
SSR1	*IKBKE*
LEP	*IL11RA*
LEP	*IL2RA*
LEP	*IL2RB*
LEP	*IL2RG*
LEP	*IL3RA*
LEP	*IL4R*
LEP	*IL5RA*
LEP	*IL6R*
LEP	*IL6ST*
LEP	*IL7R*
ANK1	*ITPR3*
CCND2	*KCNMA1*
NOTCH1	*LCK*
HNF4A	*MAPK14*
LEP	*MPL*
PPM1G	*MTNR1B*
FBXW7	*MYC*
NOTCH1	*NFKB1*
FBXW7	*NFKB2*
TCF7L2	*PARP1*
SYN2	*PDE1A*
SYN2	*PDE1B*
NOTCH1	*PIK3CG*
PAM	*PRKCA*
LEP	*PRLR*
NOTCH1	*PSMB1*
CCND2	*PYGM*
SSR1	*SERPINA1*
HNF1A	*SMO*
HNF1A	*SRC*
SYN2	*SV2A*
CCND2	*TGM2*
CCND2	*TNFRSF10B*
NOTCH1	*WEE1*
NOTCH1	*XIAP*

Red letters indicate drug targeted genes.

Supplementary Table 22 Target genes of approved T2D drug

Anti-Diabetic agents category	Target genes
insulin analog	*INSR*
human insulin	*INSR*
1st generation SU	*ABCC8, KCNJ1*
2nd generation SU	*ABCC8, KCNJ8*
3rd generation SU	*ABCC8, KCNJ1, KCNJ11*
rapid insulin secretagogue	*ABCC8*
Biguanide	*PRKAB1*
alpha-glucosidase inhibitor	*MGAM, AMY2A, SI, GAA*
Thiazolidines	*PPARG*
DPP-4 inhibitor	*DPP4*
GLP-1 analog	*GLP1R*
SGLT2 inhibitor	*SLC5A2*

Drug target genes manually extracted for genes with approved T2D drugs in Japan in June 2014 by searching DrugBank (http://www.drugbank.ca/)

Supplementary Table23. A list for biological T2D genes and their targeted drugs

Targeted genes	Targeted drugs*		Status*	Diseases*
GCK	activators	AMG 151	Phase II	Type 2 diabetes
		AZD1656	Phase II	Type 2 diabetes
		R7201	Phase II	Type 2 diabetes
		GK1-399	Phase I/II	Type 2 diabetes
		AZD6370	Phase I	Type 2 diabetes
		AZD5658	Phase I	Obesity, diabetes
		DS-7309	Phase I	Diabetes
		PSN-101	Phase I	Diabetes Mellitus Type 1 and 2
		TAK-329	Phase I	Diabetes/Type1 diabetes
		TTP355	Phase I	Type 2 diabetes
	inhibitors	Beta-D-Glucose		
		Lonidamine		
KIF11	activators	N/A		
	inhibitors	Ispinesib	Phase II	Lung Cancer
		Ispinesib	Phase II	Head and Neck Cancer, Renal Cell Carcinoma, Ovarian Cancer, Solid Tumors
		Ispinesib	Phase I	Pediatric
		SB-743921	Phase I/II	Non-Hodgkin's Lymphoma, Cancer/Tumors
		4SC-205	Phase I	Solid tumour and malignant lymphoma
		ARQ 621	Phase I	Hematological malignancies
		ARQ 621	Phase I	Late-stage solid tumors
		ARRY-520	Phase I	Cancer/Tumors
		BQS481	Phase I	Solid Tumors
GSK3B	activators	N/A		
	inhibitors	Enzastaurin	Phase III	Non Hodgkin Lymphoma
		Enzastaurin	Phase II	Glioblastoma Multiforme
		LY2090314	Phase II	AML APL
		Enzastaurin	Phase I	Brain and Central Nervous System Tumors
JUN	activators	N/A		
	inhibitors	T-5224	Phase I	Rheumatoid Arthritis

* Status in Aug 2014 by searching Therapeutic Target Database (http://bidd.nus.edu.sg/group/cjttd/)

Supplementary Table 24. Clinical Characteristics of the present study

	Stage1, set-1		Stage1, set-2		Stage2	
	T2D	Control	T2D	Control	T2D	Control
	n = 9,817	n = 6,763	n = 5,646	n = 19,420	n = 7,936	n = 5,539
male (%)	63.5	58.2	68.5	50.6	61.0	47.1
age[a]	65.7 ± 10.0	61.1 ± 11.3	66.1 ± 9.7	64.2 ± 10.7	64.1 ± 11.1	54.9 ± 16.8
BMI[a]	23.8 ± 3.8	22.4 ± 3.6	23.9 ± 3.8	22.5 ± 3.40	24.1 ± 3.9	22.4 ± 3.6

[a] Data are means ± S.D.

Supp Table 25. Direct comparison of effect sizes of novel and establised T2D loci between Japanese and Europeans

			Alleles		Japanese** 14,463 cases and 26, 183 controls				European*** 12,171 cases and 56,862 controls				Heterogeneity of the effect	
Locus	**Original GWAS**	**Lead SNPs**	**Risk**	**non-risk**	**RAF**	**OR**	**95%CI**	**p-value**	**RAF**	**OR**	**95%CI**	**p-value**	**Direction**	**Cochran's Q p-value**
MIR129-LEP	Japanese	rs4731420	C	G	0.09	1.16	(1.10 - 1.22)	7.90.E-08	0.17	0.99	(0.95 - 1.04)	7.60.E-01	+-	**1.14.E-05**
TSPAN8/LGR5	European	rs7955901	C	T	0.72	0.98	(0.95 - 1.01)	2.55.E-01	0.40	1.09	(1.05 - 1.13)	3.20.E-06	-+	**2.40.E-05**
BCAR1	European	rs7202877	T	G	0.79	0.99	(0.95 - 1.03)	5.68.E-01	0.90	1.15	(1.08 - 1.22)	2.30.E-05	-+	**3.39.E-05**
CCDC85A	Japanese novel	rs1116357	G	A	0.28	1.11	(1.07 - 1.15)	6.88.E-10	0.57	1.01	(0.98 - 1.05)	4.70.E-01	++	**1.09.E-04**
ASB3	Japanese novel	rs9309245	G	C	0.17	1.12	(1.08 - 1.17)	3.23.E-08	0.37	1.01	(0.98 - 1.05)	5.00.E-01	++	**1.33.E-04**
MIR4686	Japanese novel	rs7107784*	G	A	0.08	1.17	(1.10 - 1.24)	5.78.E-08	0.31	1.02	(0.97 - 1.07)	3.90.E-01	++	**3.71.E-04**
HMGA2	European	rs2261181	T	C	0.12	1.03	(0.98 - 1.07)	2.26.E-01	0.10	1.16	(1.10 - 1.23)	1.00.E-07	++	8.73.E-04
RBMS1	European	rs7569522	A	G	0.38	0.99	(0.96 - 1.02)	5.14.E-01	0.50	1.07	(1.04 - 1.11)	2.50.E-05	-+	9.06.E-04
ATP8B2	Japanese novel	rs67156297*	A	G	0.10	1.16	(1.10 - 1.23)	1.44.E-08	0.24	1.04	(0.99 - 1.08)	9.10.E-02	++	1.17.E-03
SSR1-RREB1	Trans ethnic	rs9505118	A	G	0.62	1.00	(0.97 - 1.03)	8.46.E-01	0.60	1.08	(1.05 - 1.12)	6.10.E-06	++	1.25.E-03
KCNQ1 int15	Japanese	rs2237896	G	A	0.60	1.33	(1.28 - 1.37)	2.81.E-70	0.94	1.14	(1.04 - 1.24)	3.70.E-03	++	1.54.E-03
GCC1	East Asian	rs6467136	G	A	0.80	1.07	(1.03 - 1.11)	1.90.E-04	0.52	0.99	(0.95 - 1.02)	5.30.E-01	+-	2.04.E-03
KLF14	European	rs13233731	G	A	0.70	1.02	(0.99 - 1.06)	1.55.E-01	0.54	1.10	(1.06 - 1.13)	4.30.E-08	++	2.07.E-03
INAFM2	Japanese novel	rs67839313*	C	T	0.27	1.09	(1.06 - 1.13)	4.79.E-07	0.11	1.01	(0.97 - 1.06)	5.30.E-01	++	5.94.E-03
MTNR1B	European	rs10830963	G	C	0.44	1.03	(1.00 - 1.07)	8.22.E-02	0.26	1.11	(1.07 - 1.16)	7.30.E-07	++	6.25.E-03
DGKB	European	rs17168486	T	C	0.39	1.05	(1.02 - 1.08)	2.29.E-03	0.17	1.13	(1.08 - 1.18)	6.90.E-07	++	7.61.E-03
CLIP2	European	rs10401969	C	T	0.10	1.01	(0.96 - 1.06)	8.12.E-01	0.10	1.13	(1.05 - 1.21)	5.40.E-04	++	8.99.E-03
ANK1	Japanese	rs12549902	A	G	0.52	1.11	(1.08 - 1.15)	1.28.E-12	0.63	1.05	(1.02 - 1.09)	3.00.E-03	++	9.48.E-03
KCNK16	East Asian	rs1535500*	T	G	0.37	1.07	(1.04 - 1.10)	1.68.E-05	0.49	1.01	(0.98 - 1.05)	4.70.E-01	++	1.37.E-02
ZBED3	European	rs6878122	G	A	0.02	0.98	(0.88 - 1.09)	6.80.E-01	0.30	1.13	(1.07 - 1.18)	1.20.E-06	-+	1.76.E-02
RASGRP1	Chinese	rs7403531	T	C	0.47	0.96	(0.93 - 0.99)	1.36.E-02	0.28	1.02	(0.98 - 1.06)	3.60.E-01	-+	2.26.E-02
ZFAND3	East Asian	rs9470794	C	T	0.22	1.08	(1.04 - 1.12)	8.11.E-05	0.12	0.99	(0.93 - 1.05)	8.00.E-01	+-	2.30.E-02
ZFAND6	European	rs11634397	G	A	0.09	1.02	(0.97 - 1.07)	5.05.E-01	0.66	1.09	(1.05 - 1.13)	7.30.E-06	++	2.48.E-02
BCL11A	European	rs243088	T	A	0.67	1.03	(1.00 - 1.07)	4.33.E-02	0.50	1.09	(1.05 - 1.13)	1.00.E-05	++	3.55.E-02
ANKRD55	European	rs459193	G	A	0.52	1.11	(1.07 - 1.14)	1.04.E-10	0.74	1.05	(1.01 - 1.09)	2.10.E-02	++	3.79.E-02
LAMA-1	European	rs8090011	G	C	0.71	1.02	(0.98 - 1.05)	3.04.E-01	0.39	1.07	(1.03 - 1.11)	1.20.E-03	++	5.30.E-02
ADAMTS9	European	rs6795735	C	T	0.18	1.02	(0.98 - 1.06)	3.40.E-01	0.52	1.07	(1.03 - 1.10)	2.30.E-04	++	7.42.E-02
DUSP8	European	rs2334499	T	C	0.80	1.02	(0.98 - 1.06)	2.70.E-01	0.46	1.07	(1.03 - 1.11)	7.30.E-04	++	8.37.E-02
GRB14	South Asian	rs3923113	A	C	0.91	1.10	(1.04 - 1.17)	7.59.E-04	0.59	1.04	(1.00 - 1.08)	3.10.E-02	++	9.50.E-02
PROX1	European	rs2075423	G	T	0.87	1.03	(0.98 - 1.08)	2.40.E-01	0.66	1.08	(1.04 - 1.12)	6.70.E-05	++	9.85.E-02
IGF2BP2	European	rs4402960	T	G	0.70	1.18	(1.14 - 1.21)	1.56.E-22	0.69	1.13	(1.09 - 1.17)	2.70.E-11	++	1.03.E-01
UBE2E2	Japanese	rs7612463	C	A	0.86	1.16	(1.12 - 1.21)	4.30.E-13	0.87	1.10	(1.04 - 1.16)	9.80.E-04	++	1.09.E-01
SLC30A8	European	rs3802177	G	A	0.56	1.11	(1.08 - 1.14)	6.96.E-11	0.76	1.16	(1.11 - 1.22)	2.10.E-11	++	1.21.E-01
GCK	European	rs10278336	A	G	0.62	1.01	(0.98 - 1.04)	5.25.E-01	0.57	1.05	(1.01 - 1.09)	2.00.E-02	++	1.35.E-01
PEPD	East Asian	rs3786897	A	G	0.54	1.06	(1.03 - 1.09)	2.48.E-04	0.61	1.02	(0.98 - 1.06)	3.10.E-01	++	1.42.E-01
MPHOSPH9	Trans ethnic	rs4275659	C	T	0.66	1.02	(0.99 - 1.06)	1.44.E-01	0.68	1.06	(1.03 - 1.10)	8.80.E-04	++	1.50.E-01
PRC1	European	rs12899811	G	A	0.99	0.98	(0.86 - 1.13)	7.98.E-01	0.27	1.09	(1.04 - 1.13)	3.30.E-05	-+	1.54.E-01
TMEM154	Trans ethnic	rs6813195	C	T	0.52	1.04	(1.01 - 1.08)	7.99.E-03	0.70	1.08	(1.04 - 1.12)	6.10.E-05	++	1.55.E-01
KCNJ11	European	rs5215	C	T	0.33	1.05	(1.01 - 1.08)	4.56.E-03	0.38	1.08	(1.05 - 1.12)	4.40.E-06	++	1.64.E-01
TP53INP1	European	rs7845219	T	C	0.31	1.04	(1.01 - 1.08)	1.70.E-02	0.45	1.08	(1.04 - 1.12)	1.40.E-05	++	1.64.E-01
ARAP1 (CENTD2)	European	rs1552224	A	C	0.96	1.21	(1.11 - 1.31)	5.12.E-06	0.88	1.13	(1.08 - 1.19)	4.90.E-07	++	1.76.E-01
GCKR	European	rs780094	C	T	0.42	1.07	(1.04 - 1.11)	2.15.E-06	0.59	1.04	(1.00 - 1.08)	2.50.E-02	++	1.87.E-01
FSCN3-PAX4	Chinese	rs10229583	G	A	0.87	1.10	(1.05 - 1.16)	7.72.E-05	0.75	1.06	(1.02 - 1.10)	2.90.E-03	++	2.20.E-01
IRS1	European	rs2943640	C	A	0.94	1.05	(0.99 - 1.11)	9.35.E-02	0.66	1.09	(1.05 - 1.12)	1.80.E-06	++	2.25.E-01
C2CD4A/B	Japanese	rs7163757	C	T	0.56	1.09	(1.06 - 1.13)	3.84.E-08	0.59	1.06	(1.02 - 1.10)	1.30.E-03	++	2.43.E-01
CDKN2A/B	European	rs10811661	T	C	0.54	1.22	(1.18 - 1.26)	2.86.E-31	0.80	1.18	(1.13 - 1.23)	1.50.E-13	++	2.54.E-01
AP3S2	South Asian	rs2028299	C	A	0.20	1.07	(1.04 - 1.11)	1.14.E-04	0.26	1.04	(1.00 - 1.09)	3.50.E-02	++	2.71.E-01
KLHDC5	European	rs10842994	C	T	0.80	1.06	(1.01 - 1.10)	1.01.E-02	0.78	1.09	(1.04 - 1.13)	1.50.E-04	++	2.73.E-01
PTPRD	Chinese	rs17584499	T	C	0.14	0.97	(0.93 - 1.01)	1.16.E-01	0.20	1.00	(0.95 - 1.06)	9.40.E-01	-0	3.33.E-01
GPSM1	Japanese	rs11787792*	A	G	0.90	1.14	(1.09 - 1.20)	2.21.E-08	0.61	1.10	(1.03 - 1.18)	3.60.E-03	++	3.53.E-01
MC4R	European	rs12970134	A	G	0.21	1.05	(1.01 - 1.10)	1.20.E-02	0.29	1.08	(1.04 - 1.12)	1.10.E-04	++	3.82.E-01
HNF1B (TCF2)	European	rs4430796	G	A	0.36	1.10	(1.07 - 1.14)	1.17.E-09	0.48	1.13	(1.07 - 1.19)	2.40.E-06	++	4.17.E-01
PSMD6	East Asian	rs831571	C	T	0.70	1.05	(1.02 - 1.09)	1.46.E-03	0.76	1.03	(0.99 - 1.08)	1.60.E-01	++	4.22.E-01
FTO	European	rs9936385	C	T	0.17	1.15	(1.11 - 1.20)	1.57.E-14	0.44	1.13	(1.09 - 1.18)	4.70.E-11	++	4.32.E-01
TLE4	European	rs17791513	A	G	0.93	1.16	(1.09 - 1.25)	1.12.E-05	0.93	1.21	(1.13 - 1.30)	1.00.E-07	++	4.39.E-01
GIPR	East Asian	rs8108269	G	T	0.62	1.08	(1.05 - 1.12)	8.43.E-07	0.24	1.06	(1.02 - 1.11)	3.10.E-03	++	4.55.E-01
HMG20A	South Asian	rs7178572	G	A	0.46	1.06	(1.03 - 1.10)	1.76.E-04	0.69	1.08	(1.04 - 1.12)	1.00.E-04	++	4.83.E-01
CCND2	European	rs11063069	G	A	0.01	1.05	(0.93 - 1.19)	4.44.E-01	0.21	1.10	(1.05 - 1.15)	1.50.E-04	++	4.85.E-01
NOTCH2	European	rs10923931	T	G	0.04	1.06	(0.94 - 1.19)	3.18.E-01	0.08	1.10	(1.05 - 1.16)	3.10.E-04	++	5.71.E-01
PPARG	European	rs1801282	C	G	0.97	1.19	(1.09 - 1.30)	8.76.E-05	0.90	1.16	(1.11 - 1.22)	5.00.E-09	++	5.74.E-01
SPRY2	Chinese	rs1359790	G	A	0.74	1.09	(1.05 - 1.12)	3.59.E-06	0.73	1.10	(1.06 - 1.14)	9.20.E-07	++	5.96.E-01
CDKAL1	European	rs7756992	G	A	0.47	1.18	(1.15 - 1.22)	6.81.E-29	0.28	1.20	(1.16 - 1.25)	1.30.E-22	++	5.97.E-01
TCF7L2	European	rs7903146	T	C	0.03	1.43	(1.33 - 1.53)	3.54.E-23	0.31	1.40	(1.35 - 1.46)	5.50.E-65	++	6.22.E-01
CDC123/CAMK1D	European	rs12779790	G	A	0.12	1.10	(1.05 - 1.15)	8.81.E-05	0.24	1.08	(1.03 - 1.13)	1.20.E-03	++	6.62.E-01
POU5F1-TCF19	Trans ethnic	rs115164593	G	A	0.54	1.08	(1.04 - 1.12)	1.15.E-04	0.75	1.09	(1.05 - 1.13)	2.00.E-05	++	6.77.E-01
ARL15	Trans ethnic	rs702634	A	G	0.87	1.07	(1.02 - 1.11)	2.02.E-03	0.71	1.08	(1.04 - 1.12)	1.80.E-05	++	6.87.E-01
GRK5	Chinese	rs10886471	C	T	0.73	1.00	(0.97 - 1.03)	9.88.E-01	0.46	0.99	(0.95 - 1.03)	5.90.E-01	--	7.18.E-01
ST6GAL1	South Asian	rs16861329	C	T	0.78	1.04	(1.00 - 1.08)	3.40.E-02	0.87	1.03	(0.97 - 1.09)	3.90.E-01	++	7.43.E-01
ZMIZ1	European	rs12571751	A	G	0.57	1.08	(1.05 - 1.12)	3.81.E-07	0.54	1.09	(1.06 - 1.13)	1.80.E-07	++	7.50.E-01
TLE1	European	rs2796441	G	A	0.36	1.08	(1.04 - 1.11)	4.00.E-06	0.60	1.07	(1.03 - 1.12)	2.50.E-04	++	7.85.E-01
KCNQ1 int11	European	rs231361	A	G	0.83	1.09	(1.05 - 1.14)	5.83.E-05	0.25	1.10	(1.05 - 1.15)	2.80.E-05	++	7.85.E-01
VPS26A	South Asian	rs1802295	T	C	0.12	1.01	(0.96 - 1.06)	6.24.E-01	0.34	1.02	(0.98 - 1.06)	2.80.E-01	++	8.15.E-01
GLIS3	East Asian	rs7041847	A	G	0.52	1.05	(1.01 - 1.08)	3.69.E-03	0.56	1.05	(1.01 - 1.09)	9.90.E-03	++	8.49.E-01
MAEA	East Asian	rs6815464*	C	G	0.64	1.11	(1.07 - 1.15)	4.93.E-10	0.99	1.12	(1.01 - 1.24)	3.30.E-02	++	8.55.E-01
HHEX/IDE	European	rs1111875	C	T	0.33	1.15	(1.11 - 1.18)	2.39.E-16	0.58	1.15	(1.11 - 1.18)	1.10.E-15	++	8.75.E-01
SRR	Chinese	rs391300	G	A	0.78	1.00	(0.97 - 1.04)	8.18.E-01	0.66	1.00	(0.96 - 1.04)	9.50.E-01	+0	8.78.E-01
HNF4A	South Asian	rs4812829	A	G	0.40	1.07	(1.03 - 1.10)	6.61.E-05	0.18	1.07	(1.02 - 1.12)	9.10.E-03	++	8.83.E-01
FAF1	Trans ethnic	rs17106184	G	A	0.94	1.10	(1.04 - 1.15)	5.25.E-04	0.91	1.10	(1.04 - 1.17)	1.10.E-03	++	9.33.E-01
WFS1	European	rs4458523	G	T	0.98	1.09	(0.97 - 1.23)	1.43.E-01	0.65	1.09	(1.06 - 1.13)	1.90.E-07	++	9.51.E-01
DMRTA1	Japanese novel	rs1575972	T	A	0.93	1.20	(1.13 - 1.29)	5.02.E-08	0.98	1.20	(1.06 - 1.35)	3.20.E-03	++	9.70.E-01
THADA	European	rs10203174	C	T	0.99		N/A		0.88	1.15	(1.08 - 1.21)	1.50.E-06	?+	
ADCY5	European	rs11717195	T	C	1.00		N/A		0.78	1.09	(1.05 - 1.14)	9.70.E-06	?+	
JAZF1	European	rs849135	G	A	1.00		N/A		0.51	1.12	(1.08 - 1.16)	3.40.E-10	?+	
HNF1A (TCF1)	European	rs12427353	G	C	1.00		N/A		0.84	1.12	(1.07 - 1.17)	1.00.E-06	?+	
LPP	Trans ethnic	rs6808574	C	T	1.00		N/A		0.61	1.08	(1.04 - 1.12)	1.30.E-04	?+	
RND3	African America	rs7560163	C	G	0.80	1.02	(0.98 - 1.07)	3.66.E-01	1.00		N/A		+?	
SLC16A13	Japanese	rs75418188	T	C	0.07	1.20	(1.13 - 1.27)	7.89.E-11	0.01		N/A		+?	
SGCG	South Asian	rs9552911	G	A	0.76	0.99	(0.96 - 1.03)	6.13.E-01	1.00		N/A		-?	
FAM60A	Japanese novel	rs147538848	A	G	0.23	1.11	(1.07 - 1.16)	1.88.E-07	0.00		N/A		+?	
PAM	European	rs35658696	G	A	0.00		N/A		0.04		N/A		N/A	

RAF; risk allele freqiency (1000 genomes project phase 3 JPT, CEU) , OR; odds ratio , 95%CI; 95% confidence interval

*Best proxy SNPs used in European populations

rs6815464-C ; rs11247991-A (r^2 = 1, CEU)

rs1535500-T; rs3734618-G (r^2 = 1, CEU)

rs11787792-G ; rs3829109-G (r^2 = 1, CEU)

rs67156297-A; rs12025518-C (r^2 = 1, CEU)

rs7107784-G; rs7111341-T (r^2 = 0.84, CEU)

rs67839313-C; rs4924456-G (r^2 = 0.84, JPT)

** Stage-1 sample in the present Japanese study

*** Publicly aveirable GWAS data (DIAGRAM3)

Supplementary Table26.12 loci categolised as Group 2 or 3 in systematic comparison of the effect sizes between Japanese and Europeans

original study	Lead SNPs	Japanese #				Cochran's Q	SNPs in LD	r^2 (JPT)	Japanese #				Cochran's Q	Group**
(population)	*Locus*	European ##						r^2 (CEU)	European ##					
		RAF	OR	95%CI	p-value	p-value§			RAF	OR	95%CI	p-value	p-value§	
European	rs7955901	0.72	0.98	(0.95 - 1.01)	2.55.E-01	**2.40.E-05**	rs7132840	0.27	0.89	1.03	(0.99 - 1.08)	1.77.E-01	1.13.E-01	2
	TSPAN8/LGR5	0.40	1.09	(1.05 - 1.13)	3.20.E-06			0.83	0.44	1.08	(1.05 - 1.12)	1.30.E-05		
	rs7202877	0.79	0.99	(0.95 - 1.03)	5.68.E-01	**3.39.E-05**	rs13337397	0.24	0.64	1.02	0.99 1.05	2.57.E-01	2.19.E-03	2
	BCAR1	0.90	1.15	(1.08 - 1.22)	2.30.E-05			0.94	0.91	1.15	1.07 1.23	7.60.E-05		
	rs10203174	0.99		N/A			No							3
	THADA	0.88	1.15	(1.08 - 1.21)	1.50.E-06									
	rs11717195	1.00		N/A			rs11707746	N/A	0.95	1.05	(0.98 - 1.13)	1.56.E-01	2.98.E-01	2
	ADCY5	0.78	1.09	(1.05 - 1.14)	9.70.E-06			0.84	0.76	* 1.10	(1.06 - 1.15)	4.30.E-06		
	rs849135	1.00		N/A			rs849134	N/A	0.81	1.04	(1.003 - 1.08)	3.27.E-02	5.87.E-03	2
	JAZF1	0.51	1.12	(1.08 - 1.16)	3.40.E-10			1.00	0.51	1.12	(1.08 - 1.16)	3.20.E-10		
	rs12427353	1.00		N/A			No							3
	HNF1A (TCF1)	0.84	1.12	(1.07 - 1.17)	1.00.E-06									
Japanese	rs4731420	0.09	1.16	(1.10 - 1.22)	7.90.E-08	**1.14.E-05**	rs791597	1.00	0.09	1.16	(1.10 - 1.22)	1.23.E-07	**2.60.E-04**	3
	MIR129-LEP	0.17	0.99	(0.95 - 1.04)	7.60.E-01			0.71	0.23	* 1.01	(0.96 - 1.06)	6.90.E-01		
	rs1116357	0.28	1.11	(1.07 - 1.15)	6.88.E-10	**1.09.E-04**	No							3
	CCDC85A	0.57	1.01	(0.98 - 1.05)	4.70.E-01									
	rs9309245	0.17	1.12	(1.08 - 1.17)	3.23.E-08	**1.33.E-04**	rs2113821	0.80	0.17	1.12	(1.07 - 1.17)	2.61.E-07	3.98.E-02	2
	ASB3	0.37	* 1.01	(0.98 - 1.05)	5.00.E-01			0.17	0.09	1.03	(0.96 - 1.10)	4.50.E-01		
	rs7107784	0.08	1.17	(1.10 - 1.24)	5.78.E-08	**3.71.E-04**	rs11564705	0.93	0.07	1.16	(1.09 - 1.22)	2.92.E-07	1.80.E-02	2
	MIR4686	0.31	* 1.02	(0.97 - 1.07)	3.90.E-01			0.76	0.26	1.05	(0.99 - 1.11)	7.90.E-02		
	rs75418188※	0.07	1.20	(1.13 - 1.27)	7.89.E-11		rs35489850	1.00	0.07	1.20	(1.13 - 1.27)	2.68.E-10	**3.91.E-08**	3
	SLC16A13	0.005		N/A				N/A	0.21	* 0.98	(0.94 - 1.03)	4.80.E-01		
	rs147538848	0.23	1.11	(1.07 - 1.16)	1.88.E-07		No							3
	FAM60A	0.00		N/A										

RAF; risk allele freqiency (1000 genomes project phase 3 JPT, CEU) , OR; odds ratio , 95%CI; 95% confidence interval

*Best proxy SNPs used in European populations

rs11707746-C ; rs11708067-G (r^2 = 0.891, CEU)

rs791597-A ; rs791595-A (r^2 = 0.705, CEU)

rs35489850-C; rs8078000-A (r^2 = 0.91, CEU)

rs67156297-A; rs12025518-C (r^2 = 1, CEU)

rs7107784-G; rs7111341-T (r^2 = 0.84, CEU)

** see Supplementary Figure8

Stage-1 sample in the present Japanese study ; 14,463 cases and 26, 183 controls

Publicly aveirable GWAS data (DIAGRAM3) ; 12,171 cases and 56,862 controls

§ significant P < 0.0071 =0.05/7

※rs312457(r2 = 0.82, JPT, r2 = 0.96, MXL) in *SLC16A13* is also present in Mexican and associated to T2D (P=8.85 x 10e-11), The SIGMA Type 2 Diabetes Consortium Nature 506 2014.

Supplementary Table27. Systematic evaluation for effect sizes and LD in 7 novel loci

SNP	Heterogeneity *			SNPs in LD				Group#
				**	***	r^2		
rs1116357	East Asian	1.21.E-02	No					1
	European	**2.56.E-04**	**Yes**	No				3
	South Asian	**1.80.E-03**	**Yes**	No				3
	Mexican	**3.30.E-03**	**Yes**	No				3
rs147538848	East Asian	7.38.E-01	No					1
	European	N/A	N/A		No			3
	South Asian	4.49.E-01	No					1
	Mexican	5.39.E-01	No					1
rs1575972	East Asian	4.21.E-01	No					1
	European	2.85.E-01	No					1
	South Asian	3.24.E-01	No					1
	Mexican	9.01.E-02	No					1
rs9309245	East Asian	4.82.E-02	No					1
	European	**2.51.E-04**	**Yes**	rs2113821		0.802 (JPT)	0.168 (CEU)	2##
	South Asian	**9.03.E-05**	**Yes**	rs2113821		0.802 (JPT)	0.27 (SA)	N/A
	Mexican	**5.28.E-04**	**Yes**	rs2113821		0.802 (JPT)	0.382 (MEX)	N/A
rs67156297	East Asian	5.59.E-02	No					1
	European	**2.88.E-03**	**Yes**	No				3
	South Asian	**1.14.E-05**	**Yes**	No				3
	Mexican	3.18.E-01	No					1
rs7107784	East Asian	1.40.E-02	No					1
	European	**5.79.E-04**	**Yes**	rs11564705		0.933 (JPT)	0.761 (CEU)	2##
	South Asian	1.85.E-01	No					1
	Mexican	9.92.E-01	No					1
rs67839313	East Asian	8.33.E-01	No					1
	European	1.43.E-02	No					1
	South Asian	5.37.E-02	No					1
	Mexican	5.02.E-01	No					1

* Heterogeneity of the effect size between Japanese (Stage-1+Stage-2) and each populations (replicaition set in the present study) significant $P < 0.0071 = 0.05/7$

** SNPs in LD ($r2 \geq 0.8$) in Japanese whereas not in LD ($r2 < 0.8$) or in the other population(s)

*** SNPs in LD ($r2 \geq 0.8$) in Japanese and not monoallelic in European

please note supplementary Figure 8 for the definition

please note supplementary Table 30

Supplementary Notes

The SIGMA (Slim Initiative in Genomic Medicine for the Americas) Type 2 Diabetes Genetics Consortium

Amy L. Williams[1,2], Suzanne B. R. Jacobs[1], Hortensia Moreno-Macías[3], Alicia Huerta-Chagoya[4,5], Claire Churchouse[1], Carla Márquez-Luna[6], Humberto García-Ortíz[6], María José Gómez-Vázquez[4,7], Stephan Ripke[1,8], Alisa K. Manning[1], Benjamin Neale[1,8], David Reich[1,2], Daniel O. Stram[9], Juan Carlos Fernández-López[6], Sandra Romero-Hidalgo[6], Nick Patterson[1], Suzanne B. R. Jacobs[1], Claire Churchhouse[1], Shuba Gopal[10], James A. Grammatikos[10], Ian C. Smith[11], Kevin H. Bullock[10], Amy A. Deik[10], Amanda L. Souza[10], Kerry A. Pierce[10], Clary B. Clish[10], Irma Aguilar-Delfín[6], Angélica Martínez-Hernández[6], Federico Centeno- Cruz[6], Elvia Mendoza-Caamal[6], Cristina Revilla-Monsalve[12], Sergio Islas-Andrade[12], Emilio Córdova[6], Eunice Rodríguez-Arellano[13], Xavier Soberón[6], María Elena González-Villalpando[22], Brian E. Henderson[9], Kristine Monroe[9], Lynne Wilkens[14], Laurence N. Kolonel[14], and Loic Le Marchand[14], Laura Riba[5], María Luisa Ordóñez-Sánchez[4], Rosario Rodríguez-Guillén[4], Ivette Cruz-Bautista[4], Maribel Rodríguez-Torres[4], Linda Liliana Muñoz-Hernández[4], Tamara Sáenz[4], Donají Gómez[4], Ulices Alvirde[4], Robert C. Onofrio[15], Wendy M. Brodeur[15], Diane Gage[15], Jacquelyn Murphy[1], Jennifer Franklin[15], Scott Mahan[15], Kristin Ardlie[15], Andrew T. Crenshaw[15], Wendy Winckler[15],

Maria L. Cortes[16], Noël P. Burtt[1], Carlos A. Aguilar-Salinas[4], Clicerio González-Villalpando[22], Jose C. Florez[1,17,18], Lorena Orozco[6], Christopher A. Haiman[9], Teresa Tusié-Luna[4,5], David Altshuler[1,2,17,18,19,20,21]

1 Program in Medical and Population Genetics, Broad Institute of Harvard and MIT, Cambridge, Massachusetts, USA
2 Department of Genetics, Harvard Medical School, Boston, Massachusetts, USA
3 Universidad Autonoma Metropolitana, Mexico City, Mexico
4 Instituto Nacional de Ciencias Médicas y Nutrición Salvador Zubirán, Mexico City, Mexico
5 Instituto de Investigaciones Biomédicas, UNAM. Unidad de Biología Molecular y Medicina Genómica, UNAM/INCMNSZ, Mexico City, Mexico
6 Instituto Nacional de Medicina Genómica, Mexico City, Mexico
7 Universidad Autónoma de Nuevo León, San Nicolás de los Garza, Nuevo León 66451, México
8 Analytic and Translational Genetics Unit, Massachusetts General Hospital, Boston, MA, USA 16 Instituto Mexicano del Seguro Social SXXI, Mexico City, Mexico
9 Department of Preventive Medicine, Keck School of Medicine, University of Southern California, Los Angeles, California,, USA
10 The Metabolite Profiling Platform, The Broad Institute of Harvard and MIT, Cambridge, Massachusetts, USA
11 Cancer Biology Program, The Broad Institute of Harvard and MIT, Cambridge, Massachusetts, USA 24 University of Minnesota, Minneapolis, Minnesota, USA 25 University of California San Francisco, San Francisco, California, USA 26 Duke-National University of Singapore Graduate Medical School, Singapore, Singapore
12 Instituto Mexicano del Seguro Social SXXI, Mexico City, Mexico
13 Instituto de Seguridad y Servicios Sociales para los Trabajadores del Estado, Mexico City, Mexico

14 Epidemiology Program, University of Hawaii Cancer Center, Honolulu, Hawaii, USA
15 The Genomics Platform, The Broad Institute of Harvard and MIT, Cambridge, Massachusetts, USA
16 Broad Institute of Harvard and MIT, Cambridge, Massachusetts, USA
17 Center for Human Genetic Research and Diabetes Research Center (Diabetes Unit), Massachusetts General Hospital, Boston, Massachusetts, USA
18 Department of Medicine, Harvard Medical School, Boston, Massachusetts, USA
19 Center for Human Genetic Research, Massachusetts General Hospital, Boston, Massachusetts, USA
20 Department of Molecular Biology, Harvard Medical School, Boston, Massachusetts, USA
21 Department of Biology, Massachusetts Institute of Technology, Cambridge, Massachusetts, USA
22 Centro de Estudios en Diabetes, Unidad de Investigacion en Diabetes y Riesgo Cardiovascular, Centro de Investigacion en Salud Poblacional, Instituto Nacional de Salud Publica, Mexico City, Mexico

Comparison of effect sizes of T2D SNP loci between Japanese and each of other populations

We have compared the effect sizes in our Japanese GWAS data (14,463 cases and 26,183 controls) and Europeans (DIAGRAM3 12,171 cases and 56,862 controls http://diagram-consortium.org/downloads.html) across novel and established T2D lead SNPs in the original reports or their proxies (Supplementary Table 25, Supplementary Figure 7). Out of 90 loci, we successfully obtained the association data of 80 lead SNPs in both populations. Of 80 SNPs examined, 6 SNP loci showed significant heterogeneity (significant p-value = 0.05/80 = 6×10^{-4}) of the effect between Japanese and Europeans (Supplementary Table 25). Overall there was no systematic difference in effect sizes between Japanese (J) and Europeans (E); effect sizes; J > E 35 SNPs, J < E 45 SNPs, binomial test, p = 0.31, although, as expected, the effect sizes of Japanese GWAS derived loci were greater in the Japanese (binomial test, p = 5.0×10^{-4}) and vice versa (binomial test, p = 2.9×10^{-3}, Supplementary Figure 7).

Next, we categorized 60 loci (14 Japanese GWAS and 46 out of 47 European GWAS derived loci; the association data for rs35658696 in *PAM* locus was not available in either Japanese GWAS data or DIAGRAM3 data) into 3 groups based on

heterogeneity of the effect sizes for the original lead SNPs or their proxies between the populations and on LD in each population as shown in Supplementary Figure 8. Group1: There is no significant heterogeneity of the effect size of the lead SNP between Japanese and European (significant p-value = 0.05/60 = 8×10^{-4}), suggesting it is likely that the causal SNPs are in LD in both of populations or the lead SNP is the causal SNP, Group2: the significant heterogeneity of the effect sizes is observed on lead SNPs, but not on SNPs which are in LD ($r^2 \geq 0.8$) with lead SNPs in the population of original study and not in LD ($r^2 < 0.8$) in the other population; the causal allele is likely to be in LD with the lead SNP in population-specific manner, Group3: The significant heterogeneity of the effect size of lead SNP between Japanese and European is observed. In addition, there is no SNP in LD ($r^2 \geq 0.8$) with lead SNPs in the population of original study and not in LD ($r^2 < 0.8$) in the other population; the effect of causal allele might be restricted to certain populations.

Of 60 loci, the lead SNPs of four European and two Japanese GWAS derived loci were not common (RAF < 0.01) in Japanese or European respectively. Then we searched variants not only in LD ($r^2 \geq 0.8$) with lead SNPs in the population of original studies but also common (RAF ≥ 0.01) in both of Japanese and Europeans to categorize them into either Group 2 or 3 (Supplementary Figure 8).

As shown in Supplementary Table 25, 26 and Supplementary Figure 8, 49 loci were categorized as Group1 and another 6 loci were categorized as Group3. Remaining 6 loci were categorized as Group 2, suggesting the causal allele of these loci are likely to be in LD with this SNP in population specific manner, however we could not identify potential causal SNPs in these loci which association for T2D were statistically significant in both of Japanese and Europeans (significant p-value = $0.05/60 = 8 \times 10^{-4}$)

We also evaluated the heterogeneity of the effect for seven novel loci identified in the present study between Japanese and each of East Asians, Europeans, South Asians and Mexicans (Supplementary Table 27).

Method for drug discovery

Prioritization of biological candidate genes from T2D susceptibility loci

We defined 286 T2D risk genes within the 90 known T2D susceptibility loci, which included SNPs with moderate linkage disequilibrium to the lead SNPs ($r^2 > 0.5$), according to data from 1000 genomes in European populations (1000 genomes CEU phase 2), in the Japanese population (1000 genomes JPT phase 2), in the population of original studies, or that were located within 25 kb of lead SNPs. Biological candidate genes were defined from the 286 T2D risk genes by adopting the following 6 selection criteria and calculating the number of satisfied criteria, as previously described [1].

1) Missense variant genes (Supplementary Table 17)

If the genes included missense variants, which were in linkage disequilibrium ($r^2 \geq 0.80$) to T2D risk lead SNPs in European, Japanese or population of original studies, they were annotated as missense variant genes.

2) eQTL genes (Supplementary Data 3)

We assessed cis-eQTL effects using 3 eQTL data sets: the study for lymphoblastoid cell lines (LCLs), adipose tissue obtained from 856 European subjects[2] (https://www.sanger.ac.uk/resources/software/genevar/), and liver tissues obtained from 427 European subjects(http://www.ncbi.nlm.nih.gov/projects/gap/eqtl/index.cgi)[3].

If data for the T2D risk lead SNPs were not available in eQTL data sets, we alternatively utilized the results of proxy SNPs in linkage disequilibrium ($r^2 \geq 0.80$) with the highest r^2 value. The significance threshold was defined as $p < 0.05$. For liver e-QTL, we searched for 3,694 eQTL SNPs with $p < 0.004$, because information of SNPs with p values ≥ 0.004 were not available in the NCBI eQTL database (http://www.ncbi.nlm.nih.gov/projects/gap/eqtl/index.cgi).

If gene expression was associated with the T2D risk lead SNPs or their proxy in European, Japanese or population of original studies, they were annotated as eQTL genes.

3) Monogenic diabetes genes (Supplementary Table 18)

We identified monogenic diabetes genes by querying PubMed using “monogenic diabetes” and “review” and selected the latest 6 articles that summarized monogenic diabetes[4-9]. If genes were shown to be causal for monogenic diabetes, they were annotated as monogenic diabetes genes.

4) Mouse phenotype genes (Supplementary Table 19, Supplementary Data 2)

We acquired knockout mouse phenotype and gene information from the Mouse Genome Informatics (MGI) database 16 on 11 August, 2014 (http://www.informatics.jax.org/)[10]. Of 286 T2D candidate genes, 169 mouse orthologs existed in the database. Overlap with the 169 genes implicated in knockout mouse phenotypes was evaluated. Among the 29

categories of phenotypes, we observed 6 categories that were significantly enriched with T2D risk genes ($p < 0.05/29 = 0.0017$, Supplementary Table 19). These included homeostasis/metabolism, liver/biliary system, endocrine/exocrine gland, growth/size/body, mortality/aging, and embryogenesis phenotypes. We defined genes for which at least 3 of the associated phenotype labels ($p < 9.2 \times 10^{-5}$) were observed in knockout mouse phenotypes as mouse phenotype genes.

5) PubMed text mining genes (Supplementary Table 20)

We used GRAIL (https://www.broadinstitute.org/mpg/grail/)[11] to search for text similarity in PubMed abstracts (connectivity) across the 286 T2D risk genes. To reduce confounding cause by already published T2D GWA analyses, we queried only abstracts that were published prior to December 2006. We entered the 90 T2D regions defined in the previous section (±25 kb or $r^2 > 0.50$) as query regions, and these were set equal to seed regions. Then, genes that were prioritized by PubMed text mining using GRAIL with gene-based $p < 0.05$ were annotated as PubMed text mining genes.

6) Protein-protein interaction (PPI) network genes (Supplementary Figure 4)

We used DAPPLE (http://www.broadinstitute.org/mpg/dapple/dappleTMP.php)[12] to identify genes prioritized by PPI network. We entered 90 T2D regions defined in the previous section (±25 kb or r^2>0.50) as inputs and defined the cutoff number for Common

Interactor binding degree as 2. We defined the genes with corrected $p < 0.05$ as PPI network genes.

Definition of biological T2D risk genes

Genes included in the T2D risk loci were scored by adopting the 6 selection criteria and calculating the number of the satisfied criteria (Supplementary Data 4). Since these criteria exhibited weak correlations with each other ($r^2 < 0.31$, Supplementary Figure 5), each gene was given a score based on the number of criteria that were met (scores ranged from 0 to 6). Genes with a score of at least 2 were defined as biological T2D risk genes (Figure 3, Supplementary Figure 5).

Drug target gene enrichment analysis

We obtained a list of 871 drug target genes from the previous report by Okada et al[1]. Drug target genes were manually extracted for genes with approved T2D drugs in Japan in June 2014 by searching DrugBank (http://www.drugbank.ca/) [13] or Therapeutic Target Database (http://bidd.nus.edu.sg/group/cjttd/)[14] (Supplementary Table 22). We also extracted genes in direct PPI with the biological T2D risk genes described above using the InWeb database (http://www.broadinstitute.org/mpg/dapple/dappleTMP.php) [12]. An

overlap between the biological T2D risk genes or genes in direct PPI with the biological T2D risk genes and the drug target genes was assessed using a permutation procedure, as previously described in Okada et al[1] (Supplementary Figure 6).

Supplementary References

1. Okada, Y. *et al.* Genetics of rheumatoid arthritis contributes to biology and drug discovery. *Nature* **20**, 376-381 (2014).
2. Grundberg, E. *et al.* Mapping cis- and trans-regulatory effects across multiple tissues in twins. *Nat. Genet.* **44**, 1084–1089 (2012)
3. Schadt, E.E.*et al.* Mapping the genetic architecture of gene expression in human liver. *PLoS Biol.* 6, e107. (2008)
4. Schwitzgebel, V.M. Many faces of monogenic diabetes. *J Diabetes Investig.***5**,121-133. (2014)
5. Bonnefond, A. *et al.* Highly sensitive diagnosis of 43 monogenic forms of diabetes or obesity through one-step PCR-based enrichment in combination with next-generation sequencing. *Diabetes Care.* **37**, 460-467.(2014)
6. Rubio-Cabezas, O *et al.* Diabetes mellitus in neonates and infants: genetic heterogeneity, clinical approach to diagnosis, and therapeutic options. *Horm. Res. Paediatr.* **80**,137-146. (2013)
7. Parker, V.E. *et al.* Genetics in endocrinology: genetic forms of severe insulin resistance: what endocrinologists should know. *Eur J Endocrinol.* **169**, R71-80. (2013)

8. Klupa, T. *et al.* Monogenic models: what have the single gene disorders taught us? *Curr Diab Rep.* **12**, 659-666. (2012)

9. Semple, R.K. *et al.* Genetic syndromes of severe insulin resistance. *Endocr Rev.* **32**,498-514. (2011)

10. Eppig, J.T. et al. The Mouse Genome Database (MGD): comprehensive resource for genetics and genomics of the laboratory mouse. *Nucleic Acids Res* **40**, D881-886 (2012)

11. Raychaudhuri, S. et al. Identifying relationships among genomic disease regions: predicting genes at pathogenic SNP associations and rare deletions. *PLoS Genet* **5**, e1000534 (2009)

12. Rossin, E.J. et al. Proteins encoded in genomic regions associated with immune-mediated disease physically interact and suggest underlying biology. *PLoS Genet* **7**, e1001273 (2011)

13. Wishart, D.S. et al. DrugBank: a comprehensive resource for in silico drug discovery and exploration. *Nucleic Acids Res*. 34:D668-672. (2006)

14. Zhu, F.et al. Therapeutic target database update 2012: a resource for facilitating target-oriented drug discovery. *Nucleic Acids Res*. 40: D1128-1136. (2012)
